# Transcriptome Analysis Reveals That Alfalfa Promotes Rumen Development Through Enhanced Metabolic Processes and Calcium Transduction in Hu Lambs

**DOI:** 10.3389/fgene.2019.00929

**Published:** 2019-10-03

**Authors:** Bin Yang, Hongwei Chen, Jiawen Cao, Bo He, Shanshan Wang, Yang Luo, Jiakun Wang

**Affiliations:** MoE Key Laboratory of Molecular Animal Nutrition, Institution of Dairy Science, College of Animal Sciences, Zhejiang University, Hangzhou, China

**Keywords:** alfalfa intervention, rumen development, transcriptome, calcium transduction, metabolism

## Abstract

A healthy gut is very important for young animal development. The rumen of ruminants expands in size with the colonization of microbiota by 2 months of age. This process is promoted by alfalfa intervention. To elucidate the mechanism of this promotion, we performed transcriptomic analyses using a cohort of 23 lambs to evaluate the effects of starter diets plus alfalfa on the development of the rumen wall from the pre- to the postweaning period. The quantitative PCR analyses were used to validate selected genes that were differentially expressed in the transcriptome mapping. We found that several metabolic processes associated with rumen tissue development were affected by solid feed intake, with genes linked to the calcium signaling transduction pathway and the metabolism of pteridine-containing compounds and homocysteine metabolic process being upregulated in the group with alfalfa intervention. The results suggest that the pteridine-containing compounds and calcium signaling are targets for precise regulation of rumen development.

## Introduction

The rumen is a unique organ that acts as a fermentation chamber containing a mixture of anaerobic bacteria, archaea, protozoa, anaerobic fungi, and flagellates, and it converts low-nutritional-value lignocellulose-rich plant materials into animal protein (Mackie, 2002). The muscular contractions of the rumen–reticulum wall propel the bolus of plant and saliva from the rumen into the mouth for rumination and increased plant degradation. It is also a metabolic organ that is responsible for the absorption and conversion of short-chain fatty acids into ketone bodies, which can support the requirements of animals for energy and tissue building (Baldwin et al., 2004; Reynolds and Kristensen, 2008). In adult animals, the rumen takes up a large proportion of the body cavity, while it remains undeveloped, with a small volume and thin wall, in the newborn ruminants. The rumen undergoes extensive morphological structure changes and a functional transition during a short time after solid feed intake (Baldwin et al., 2004), so the rumen has been of interest not only regarding fermentation efficiency but also as a unique model for investigating the nutrient–gene interactions and microbiome–host causal effects.

Roughage intervention in the neonatal digestive tract is a controversial operation. Although the mature rumen has to deal with roughage and has a unique fiber digestibility, and roughage feeding systems have been supported by some investigations (Castells et al., 2012; Terre et al., 2013), the delicate stomach after birth keeps breeders and especially feed companies from taking the risk of giving newborns roughage. Dehydrated alfalfa, as a tender forage containing protein and pectin, is suitable for use as roughage for the young rumen, as it provides nutrients and physical stimulation. Our previous study showed that early supplementation of starter pellets with alfalfa improved the performance of pre- and postweaning Hu lambs (Yang et al., 2015), and the alfalfa intervention stimulated the rumen microbial community development for better feed intake and animal performance before and after weaning (Yang et al., 2018). The rumen is a critical organ for the protective and metabolic functions of productive ruminants, but the nutrient–gene interactions underlying its function are still unknown. Therefore, this study compared the gene expression patterns, gene pathways, and gene networks of the rumen wall of Hu lambs fed a starter diet vs starter plus alfalfa from the pre- to the postweaning period. Understanding the critical regulatory pathways influencing the ontological responses of rumen development to alfalfa intervention will be helpful to manage animal health and identify potential new nutrient-derived approaches to promote the functioning of the rumen.

## Materials and Methods

### Animal Experiment and Sample Collection

The experimental protocols were approved by the Animal Care Committee of Zhejiang University (Hangzhou, China, ZJU2015-446-10), and our experimental procedures accorded with the guidelines for animal research of Zhejiang University.

Rumen samples used for the present study were collected from a previous animal experiment (Yang et al., 2015). Briefly, 6 out of 66 male 10-day-old Hu lambs were sacrificed as baseline (B-10), and another 60 lambs were randomly assigned into two types of feeding programs, with 2 lambs who had similar body weight within the same feeding programs sharing one 1.4×1.4 m^2^ pen, to characterize the effect of alfalfa intervention on rumen development. The difference between the two feeding programs was in the preweaning period, from d10 to d38. The lambs in the STA feeding group were fed with milk replacer and *ad libitum* starter pellets, and the lambs in the S-ALF feeding group were additionally fed with *ad libitum* chopped alfalfa. Six lambs (three pens) of each feeding group were sacrificed randomly on d17, d24, d38, d45, and d66. After slaughter, rumen tissue was collected, kept on ice, and then rinsed with precooled sterilized PBS (4°C, pH = 6.8). Before three 1.5×1.5 cm^2^ pieces of rumen tissue were obtained from the left side, right side, and ventral sac for morphological measurements of epithelial and muscular thickness (Yang et al., 2015), a 1×1 cm^2^ piece of rumen tissue sample was obtained from the ventral sac, further rinsed with precooled RNA inhibitor reagent (containing 2.8 mol/L (NH_4_)_2_SO_4_, 0.2 mol/L Tris, and 2×10^−3^ mol/L EDTA; pH = 8.15; 4°C) to prevent RNA degradation, and then snap-frozen in the liquid nitrogen until they were stored at −80°C for long-term preservation and transcriptome analysis.

### RNA Extraction and Sequencing

To reflect the changes of rumen tissue in response to weaning stress, the rumen tissues of the day before solid feed intake (B-10), the day before weaning (d38), and 7 days after weaning (d45) were chosen for RNA sequencing. Total RNA from the rumen tissues, including the epithelium and muscle layer, was extracted using a total RNA extraction kit (Aidllab, Beijing, China) according to the instructions. The concentration and purity of the extracted RNA was measured with a Nanodrop 2000 (Thermo Scientific, Wilmington, DE) and 1% agarose gel electrophoresis. RNA integrity was assessed using the RNA Nano 6000 Assay Kit of the Bioanalyzer 2100 system (Agilent Technologies, CA, USA). Only RNA samples with integrity greater than 7 were used for later RNA sequencing library construction. After screening for RNA quality, the total number of successfully processed samples was 23, with 5 samples in B-10, STA-38, and STA-45, and 4 samples in S-ALF-38 and S-ALF-45, respectively, less than the designed 30 samples.

For each of the 23 high-quality RNA samples, a total of 3 μg RNA per sample was used to generate a cDNA library using the NEBNext UltraTM RNA Library Prep Kit for Illumina® (NEB, USA) following the manufacturer’s recommendations. Library fragments were then purified and amplified, and the Agilent Bioanalyzer 2100 system was used to assess the library quality. The libraries were then sequenced on an Illumina HiSeq 4000 to generate 150 bp paired-end reads by Novogene Bioinformatics Technology Co., Ltd. (Tianjing, China).

### Transcriptome Mapping

Reads containing an adapter or poly-N and low-quality reads that did not pass the Illumina chastity filter with CASAVA (version 1.8, Illumina) were discarded. TopHat2 (v2.0.13) (Kim et al., 2013) with Bowtie2 (v2.3.3.1) (Langmead and Salzberg, 2012) was used to align the high-quality reads to the sheep reference genome (*Ovis aries* v3.1). The BAM alignment files that were generated from TopHat2 were sorted by name and converted into SAM files using Samtools (v1.1) (Li et al., 2009). HTSeq-count (v0.9.1) (Anders et al., 2015) was used to count the number of reads per gene with the SAM files based on the annotation files (http://www.ensembl.org/info/data/ftp/index.html).

### Differentially Expressed Gene Analysis

After normalization, the expression levels of mRNA in each library were calculated by counts per million reads (CPM). Genes with CPM ≥1 in at least three lambs within at least one group were considered expressed genes and were subjected to principal component analysis (PCA), differentially expressed (DE) gene analysis, and gene pattern analysis. The DE gene analysis was performed with pairwise comparisons between d38 and B-10 and between d45 and d38 in the STA and S-ALF groups using the Bioconductor (v3.6) package edgeR (v3.16.5) (Robinson et al., 2010; McCarthy et al., 2012) in R software (v3.3.3). Genes with *FDR* ≤0.05, |log_2_fold change| ≥1, and their expressions of all group members within one group consistently numerically higher or lower than the other group were considered DE genes.

### Functional Gene Annotation

The Ensembl gene ID list of the DE genes was entered into the Database for Annotation, Visualization, and Integrated Discovery (DAVID) (Huang et al., 2007; Huang da et al., 2009) for Gene Ontology (GO) term analysis and Kyoto Encyclopedia of Genes and Genomes (KEGG) pathway analysis. In the GO term analysis, GO_BP_FAT, GO_CC_FAT, and GO_MF_FAT categories were chosen to describe the subjected genes. A GO term or KEGG pathway was regarded as significant if the *P*-value was ≤0.05 and more than three DE genes were found under that term or pathway.

### Quantitative PCR Validation of Selected Genes

To validate the results of RNA sequencing, 10 genes, including glycoprotein NMB (*GPNMB*), pyruvate dehydrogenase kinase 4 (*PDK4*), mechanistic target of rapamycin kinase (*mTOR*), mitogen-activated protein kinase 6 (*MAPK6*), lipoprotein lipase (*LPL*), isocitrate dehydrogenase 2 (*IDH2*), NME/NM23 nucleoside diphosphate kinase 4 (*NME4*), activating transcription factor 4 (*ATF4*), calpastatin (*CAST*), and apolipoprotein D (*APOD*) were randomly chosen. Eight genes involved in membrane depolarization and muscle tissue development, including gap junction protein alpha 1 (*GJA1*), caveolin 3 (*CAV3*), nitric oxide synthase 2 (*NOS2*), RAR-related orphan receptor A (*RORA*), SH3 and cysteine-rich domain (*STAC*), PPARG coactivator 1 alpha (*PPARGC1A*), protein kinase C beta (*PRKCB*), and SET and MYND domain-containing 1 (*SMYD1*), were directionally selected for quantitative PCR (qPCR) analysis. The reverse transcriptome was used to generate cDNA from the total RNA of each sample using the ReverTra Ace qPCR RT Kit (Toyobo, Osaka, Japan). For each 20 µl reverse transcription volume, 1 µg total RNA were used according to the instruction. Primers used in the present study were obtained from published papers or the qPCR Primer Database (qPrimerDB; https://biodb.swu.edu.cn/qprimerdb/) as listed in **Table S1**, except for *PPARGC1A* and *PRKCB*, which were designed with the Primer-BLAST tool in the Basic Local Alignment Search Tool (BLAST, https://blast.ncbi.nlm.nih.gov/Blast.cgi) of the National Center for Biotechnology Information (NCBI). The amplification products were sequenced and searched with BLAST to validate the specificity of these primers. The qPCR (20 µl reaction mixture) with FastStart Universal SYBR Green Master (Roche, Basel, Switzerland) was performed on the ABI 7500 Real-Time PCR system (Applied Biosystems Inc., Foster City, CA, USA). The relative mRNA expression levels were normalized to the expression of *GAPDH*, a normal housekeeping gene in rumen epithelium (Liu et al., 2013) and smooth muscle cells (Pullmann et al., 2005), using 2^−(Ct^ *^target genes^* ^−Ct^ *^GAPDH^* ^)^.

### Statistical Analysis

For the rumen epithelial and muscular thickness analysis, the mean value of the left side, right side, and ventral sac was calculated and subjected to SPSS 20.0 (SPSS, Inc., Chicago, IL, United States). One-way ANOVA was performed to analyze the effects of age from d10 to d66 and the differences between the STA and S-ALF groups. Tukey’s multiple comparison test of the mean values was performed among ages from d10 to d66 in the STA and S-ALF groups. The relative mRNA expression levels obtained by qPCR validation were subjected to SPSS for the normal distribution test. The relative expression levels of *mTOR*, *MAPK6*, *IDH2*, *NME4*, *ATF4*, *CAV3*, *RORA*, *SMYD1*, *STAC*, and *PPARGC1A* were normally distributed, and one-way ANOVA was performed for pairwise comparisons between different ages in the STA and S-ALF groups. Other genes were non-normally distributed, and the Kruskal–Wallis signed rank test was performed for pairwise comparisons.

### Data Availability

The RNA sequencing data have been uploaded to the Sequence Read Archive, with the access number PRJNA540396.

## Results

### Morphological Change of Rumen Wall

Weaning transition decreased (*P* <0.05) rumen epithelial thickness in both feeding programs of STA and S-ALF. However, the S-ALF feeding program increased (*P* <0.05) muscular thickness before weaning and maintained this advantage 1 week after weaning (**Table S2**).

### Rumen Tissue Transcriptome Profiles During Pre- and Postweaning

Across all 23 rumen tissue RNA samples, 535.8 million high-quality paired reads were obtained by RNA sequencing, with an average of 23.3 ± 3.1 (mean ± SD) million reads per sample (**Table S3**). The alignment rate to the *Ovis aries* genome was 69.0 ± 2.6%. A total of 14,212 genes were identified as expressed genes (13,447 ± 158 genes per sample), which showed large variations among lambs within B-10 and between the lambs of B-10 and other groups by PCA analysis (**Figure S1**).

**Figure 1:**
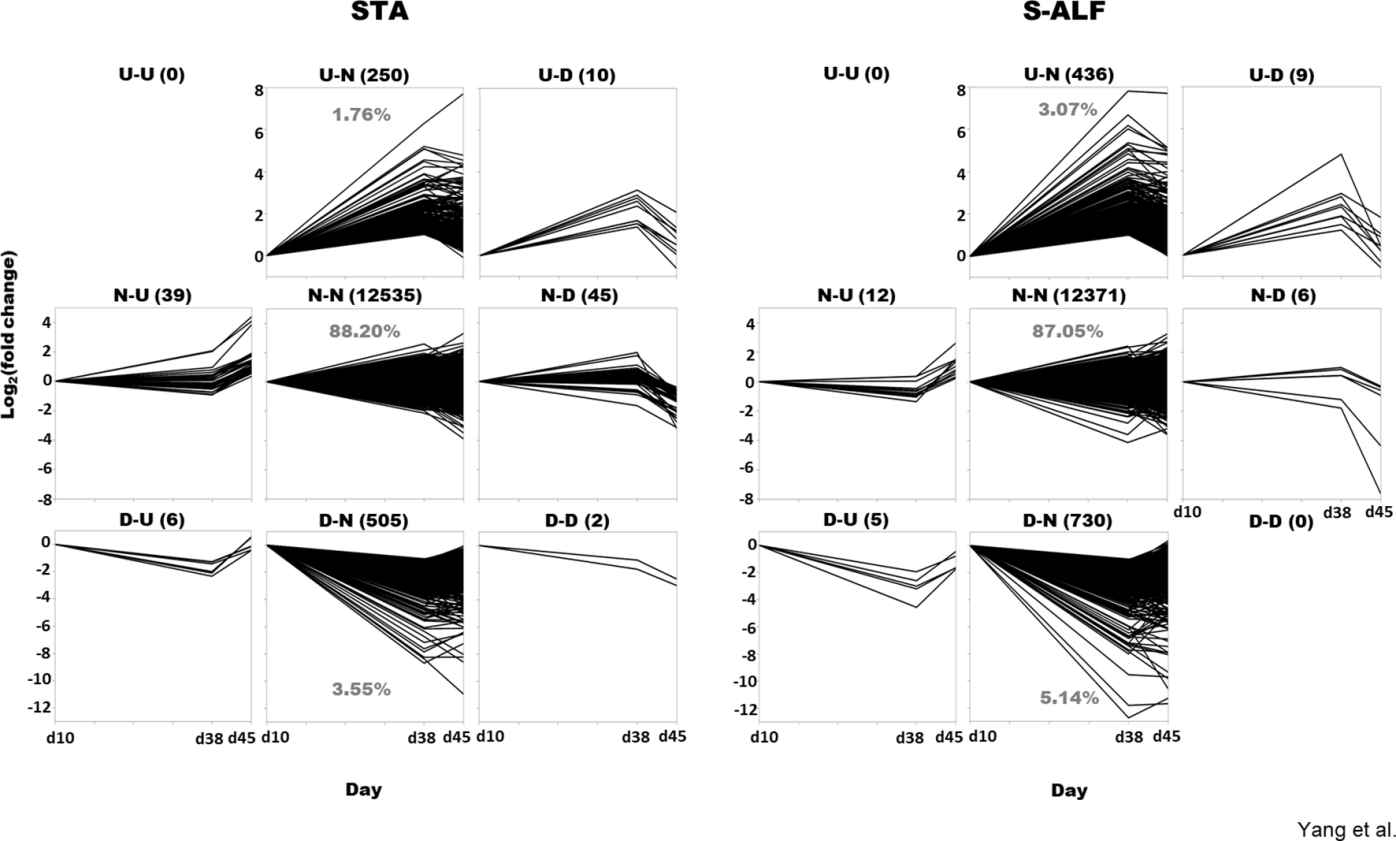
Gene pattern analysis in lambs fed a starter diet with (S-ALF) or without alfalfa intervention (STA) during early life. The comparisons of d38 vs d10 and d45 vs d38 were made within the STA and S-ALF groups to analyze the changes during the pre- and postweaning periods, respectively. “U” represents upregulated, “N” represents not changed, and “D” represents downregulated. For example, the U-N pattern is upregulated during the preweaning period but not changed during the postweaning period.

The fluctuation of gene expression before (d38 vs d10) and after weaning (d45 vs d38) was assembled into nine patterns: both upregulated (U-U), both downregulated (D-D), or not differentially expressed (N-N) before and after weaning; differentially expressed only during pre- or postweaning (U-N, D-N, N-U, and N-D); and oppositely expressed between before and after weaning (U-D, D-U, **Figure 1**). In fact, the U-U pattern was not observed in either the STA or the S-ALF group, and the D-D pattern was not observed in the S-ALF group (**Figure 1**). The N-N patterns represented 88.20% and 87.05% of the expressed genes, respectively, in the STA and S-ALF groups, followed by the D-N pattern (3.55% and 5.14% of expressed genes in STA and S-ALF, respectively) and the U-N pattern (1.76% and 3.07% of expressed genes in STA and S-ALF, respectively). The other patterns of N-U, N-D, U-D, D-U, and D-D contained fewer than 50 expressed genes in both groups (**Figure 1**).

### Differential Gene Expression in Rumen Tissues Between STA and S-ALF Groups Within N-N Patterns

Within the N-N pattern, 11,934 genes were shared between diet groups, and 601 and 437 genes were specific to the STA and S-ALF groups, respectively (**Figure S2A**). Although there were specific genes in the N-N pattern, the major enriched GO terms were similar between the STA and S-ALF groups (**Figure S2B** and **Table S4**). Gene expression, biosynthetic processes of cellular macromolecules, aromatic compounds, heterocycle- and nucleobase-containing compounds, RNA metabolic processes, protein modification processes, and the regulation of these processes were the major biological processes. Extracellular vesicle, exosome and organelle, cellular nucleoplasm, mitochondrion, cytosol, microtubule cytoskeleton, Golgi apparatus, and endoplasmic reticulum were the major cellular components. Binding of organic cyclic compounds, heterocyclic compounds, nucleic acids, ion small molecules, and carbohydrate derivatives were the top molecular functions.

### Differential Gene Expression in Rumen Tissues Between STA and S-ALF Groups Within the U-N Pattern

**Figure 2:**
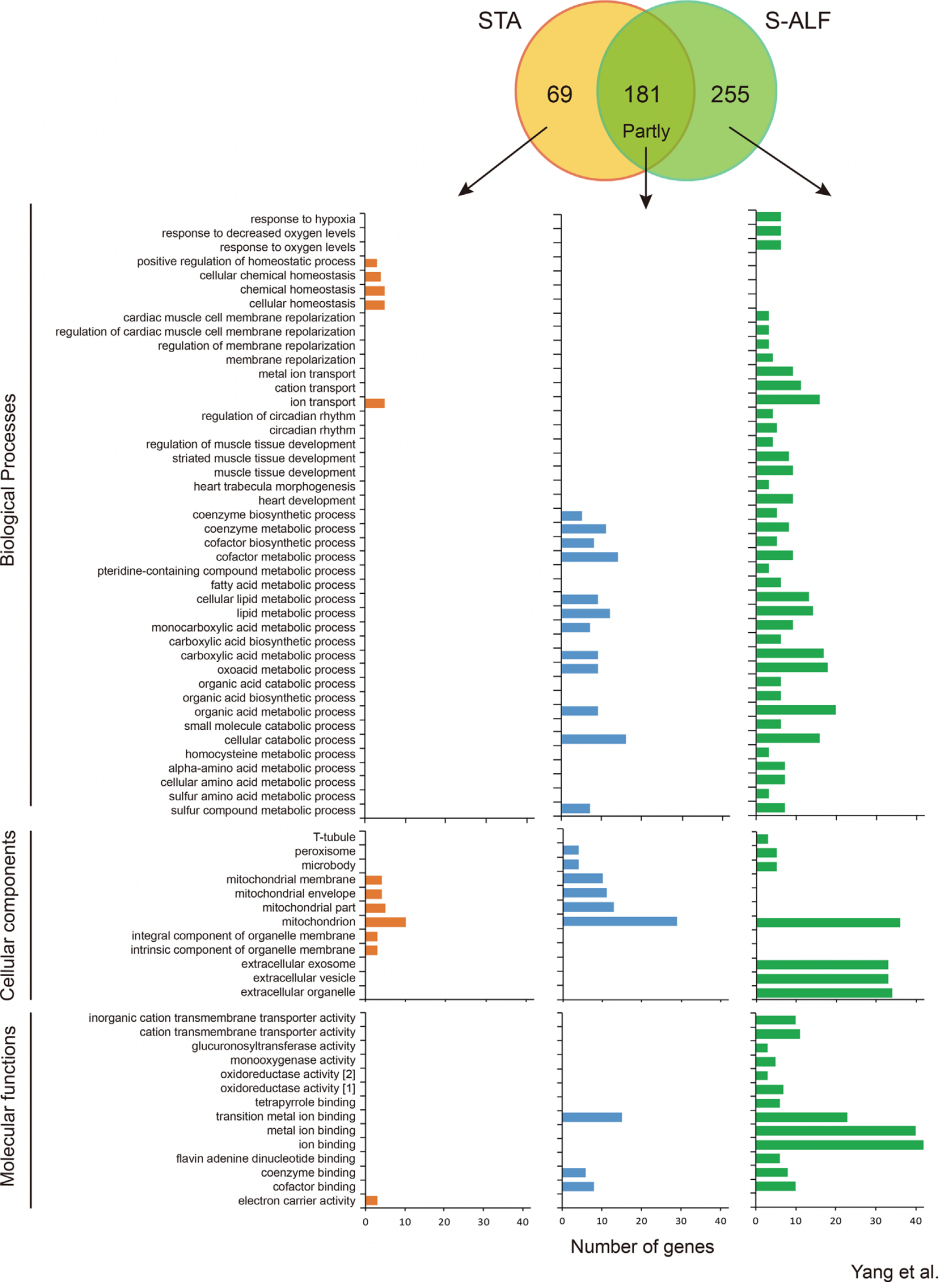
Number of genes and Gene Ontology term enrichment of the U-N pattern in lambs fed the starter diet with (S-ALF) or without alfalfa intervention (STA) during early life. [1] = oxidoreductase activity, acting on paired donors, with incorporation or reduction of molecular oxygen; [2] = oxidoreductase activity, acting on paired donors, with incorporation or reduction of molecular oxygen, NAD(P)H as one donor, and incorporation of one atom of oxygen.

Within the U-N pattern, 181 genes were shared, and 69 and 255 genes were specifically harbored by the STA and S-ALF groups, respectively (**Figure 2**). In total, 55 biological processes, nine cellular components, and seven molecular functions were upregulated in the preweaning period and remained stable during the postweaning period in both groups (**Figure 2** and **Table S5**). These biological processes mainly included some metabolic, catabolic, and biosynthetic processes, especially cofactor, organophosphate, nucleotide, nucleoside phosphate, lipid, coenzyme, ribonucleotide, ribose phosphate, carboxylic acid, oxoacid, organic acid, and cellular lipid metabolic processes and single-organism catabolic processes, which were different from the biological processes of the N-N pattern. The cellular components that predominated were the mitochondrion, organelle envelope, microbody, and peroxisome. The molecular functions that predominated were the binding of transition metal ions, cofactors, coenzymes, carbon–carbon lyase activity, and hydro- and oxidoreductase activity. The KEGG pathway analysis showed that 11 pathways were upregulated in the preweaning period and remained stable during the postweaning period in both groups, including the metabolism of sulfur, arachidonic acid, glycine, serine and threonine, cysteine and methionine, and butanoate; steroid hormone biosynthesis; synthesis and degradation of ketone bodies; valine, leucine, and isoleucine degradation; the sulfur relay system; and the peroxisome (**Figure S3**).

With the alfalfa intervention, the metabolic, catabolic, and biosynthetic processes were further increased before weaning, especially the metabolic processes of pteridine-containing compounds, fatty acid, homocysteine, alpha-amino acids, cellular amino acids, and sulfur amino acids and biosynthetic processes of carboxylic acid and organic acids, which were only enriched among the S-ALF–specific genes (**Figure 2**). Along with these processes, response to hypoxia, decreased oxygen levels, and oxygen levels; membrane repolarization and their regulation; metal ion and cation transport; circadian rhythm and its regulation; and muscle tissue development, heart development, and heart trabecula morphogenesis were also only enriched among the S-ALF–specific genes (**Figure 2**). The positive regulation of homeostatic processes, cellular homeostasis, and chemical homeostasis were only enriched among STA-specific genes. The t-tubule, extracellular exosome, vesicle, and organelle were only enriched among S-ALF–specific genes, while the integral and intrinsic components of the organelle membrane were only enriched among STA-specific genes (**Figure 2**). The activity of the cation transmembrane transporter, glucuronosyltransferase, monooxygenase, and oxidoreductase and the binding of tetrapyrroles, ions, and flavin adenine dinucleotides (FADs) were only enriched among S-ALF–specific genes, while the electron carrier activity was only enriched among STA-specific genes (**Figure 2**).

Despite the metabolic KEGG pathways that were further increased with alfalfa intervention, the metabolism of fatty acids, carbon, tryptophan, propanoate, glyoxylate and dicarboxylate, ascorbate and aldarate, retinol, drugs, and xenobiotics by cytochrome P450; biosynthesis and degradation of antibiotics; elongation of fatty acids; retrograde endocannabinoid signaling; glutamatergic synapses; and HIF1 signaling pathway were only enriched among S-ALF–specific genes, while the inflammatory mediator regulation of TRP channels was only enriched among STA-specific genes (**Figure S3**).

### Differential Gene Expression in Rumen Tissues Between the STA and S-ALF Groups Within the D-N Pattern

**Figure 3:**
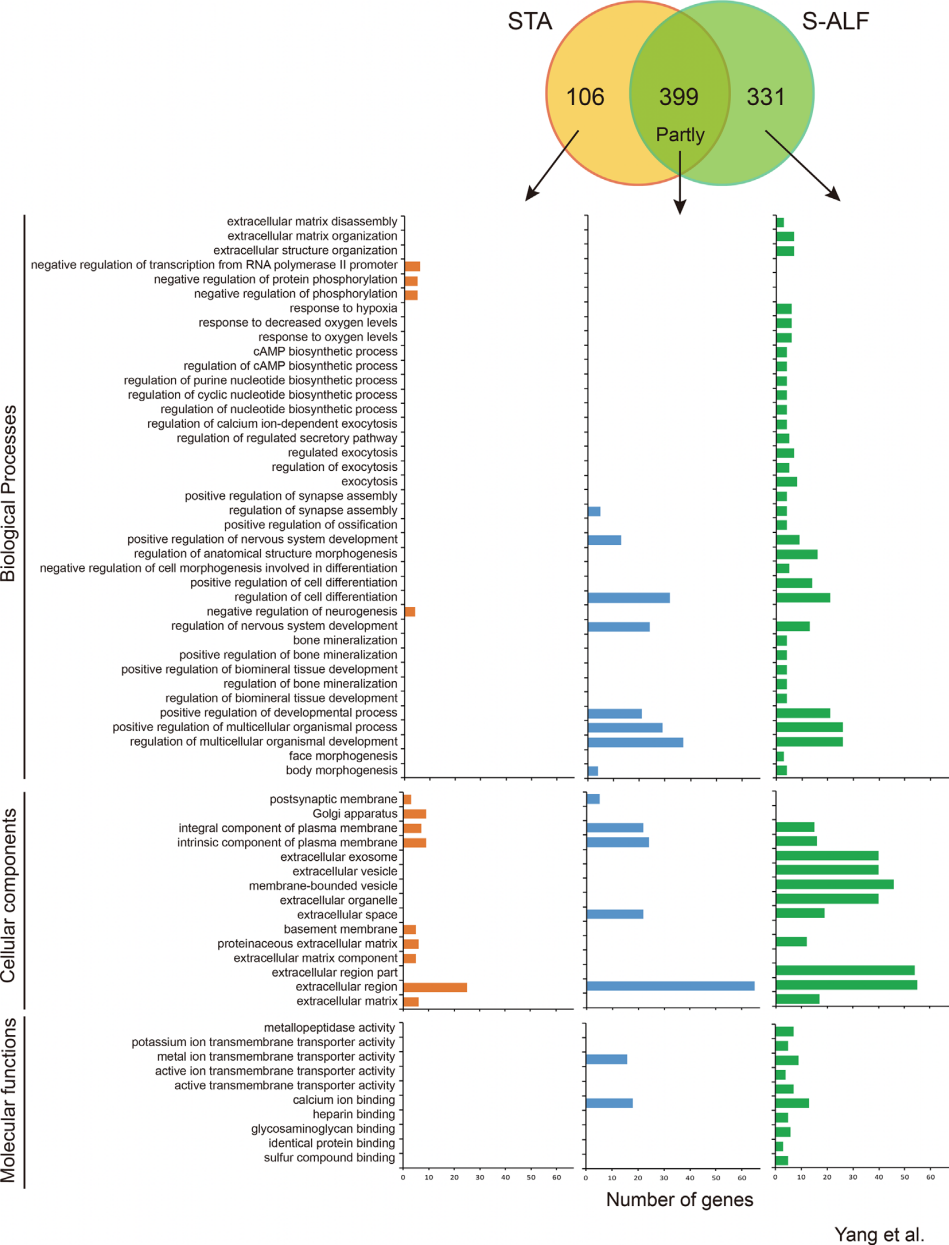
Number of genes and Gene Ontotlogy term enrichment of the D-N pattern in lambs fed the starter diet with (S-ALF) or without alfalfa intervention (STA) during early life.

Within the D-N pattern, there were 399 shared genes and 106 and 331 specific genes observed in the STA and S-ALF groups, respectively (**Figure 3**). In total, 143 biological processes, 36 cellular components, and 30 molecular functions were downregulated in the preweaning period and remained stable during the postweaning period in both groups (**Table S6**). The biological processes that predominated were the development of the nervous system and cell; regulation of signaling, cell communication, and multicellular organismal development; neurogenesis, generation of neurons, and neuron differentiation; system process; and cell projection organization (**Table S6**). The cellular components that predominated were mainly the extracellular and plasma membrane region, the neuron and synapse, the intrinsic and integral components of the plasma membrane, neuron projection, and the somatodendritic compartment. The molecular functions that predominated were the transmembrane transporter activity of cations (passive), inorganic cations, metal ions, and monovalent inorganic cations; channel activity of ions and cations (substrate-specific), gated; and calcium ion binding. The biological process “response to hypoxia and oxygen levels” was enriched among the specific genes of the S-ALF group in both the U-N and D-N patterns, but the other 28 biological processes enriched among the specific genes of the S-ALF group in the D-N pattern were completely different from those in the U-N pattern (**Figures 2** and **3**). They were enriched in extracellular matrix organization, the nucleotide biosynthetic process, exocytosis, cell differentiation, regulation of ossification, and so on. The negative regulation of phosphorylation, neurogenesis, and transcription from the RNA polymerase II promoter were only enriched among the specific genes of the STA group. The KEGG pathways “protein digestion and absorption,” “regulation of lipolysis in adipocytes,” and “gastric acid secretion” were only enriched among the specific genes of the S-ALF group, while the metabolism of beta-alanine and glycerolipid pathways were only enriched among the specific genes of the STA group (**Figure S4**).

### Differential Gene Expression in Rumen Tissues Between the STA and S-ALF Groups Within the N-U Pattern

**Figure 4:**
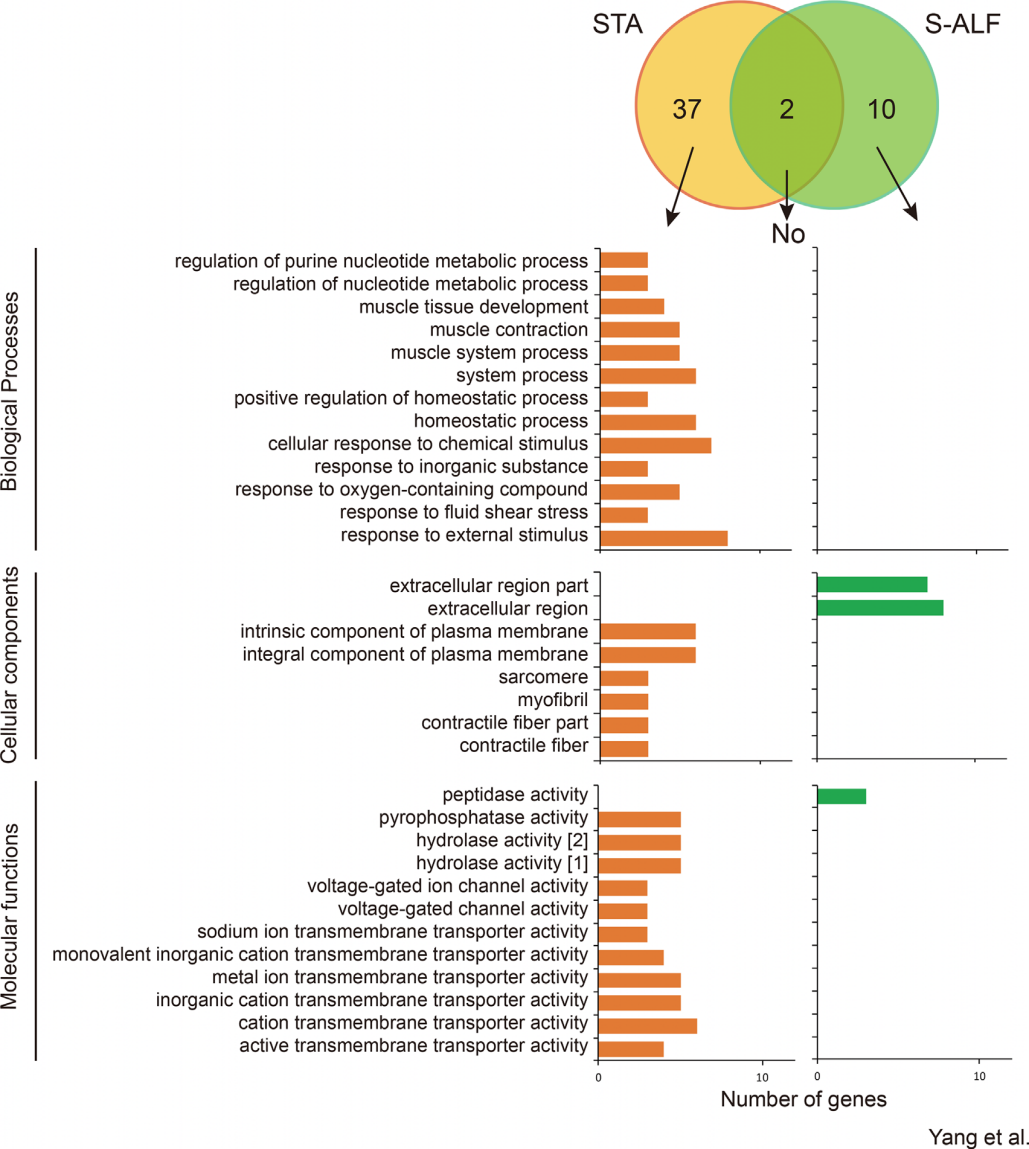
Number of genes and Gene Ontotlogy term enrichment of N-U pattern in lambs fed the starter diet with (S-ALF) or without alfalfa intervention (STA) during early life. [1] = hydrolase activity, acting on acid anhydrides; [2] = hydrolase activity, acting on acid anhydrides, in phosphorus-containing anhydrides.

Within the N-U pattern, only 2 genes were shared by the STA and S-ALF groups, while 37 and 10 specific genes were observed in the STA and S-ALF groups, respectively (**Figure 4**). No biological processes were enriched among the shared or S-ALF–specific genes. Muscle development, contraction, and system process; homeostatic process; and response to chemical stimulus, oxygen-containing compounds, fluid shear stress, and external stimulus were enriched among STA-specific genes. The KEGG pathways enriched among the STA-specific genes were associated with “arrhythmogenic right ventricular cardiomyopathy,” “hypertrophic cardiomyopathy,” “dilated cardiomyopathy,” “salivary secretion,” and “tight junction” (**Figure S5**). No pathway was enriched among the S-ALF–specific genes.

### Gene Coregulatory Network Related to Rumen Development Affected by Alfalfa

**Figure 5:**
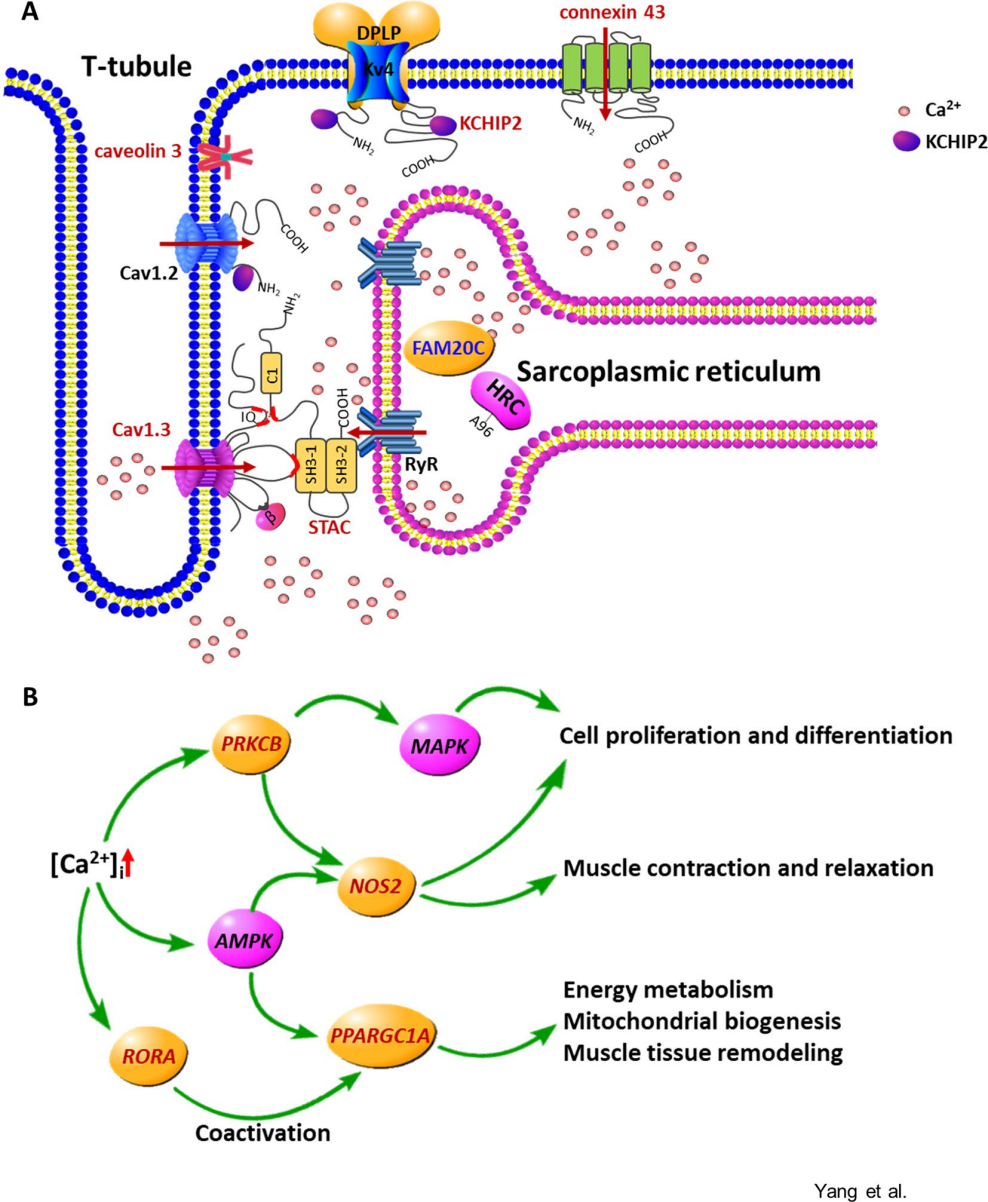
The calcium signaling pathway is overrepresented in the specific differentially expressed genes altered by alfalfa intervention during the preweaning period. **(A)** Molecules involved in the increase and removal of the intracellular calcium ([Ca^2+^]_i_). Cav1.3, L-type Ca^2+^ channel subunit alpha 1 D; Cav1.2, L-type Ca^2+^ channel subunit alpha 1 C; STAC, SH3 and cysteine-rich domain-containing protein; RyR, ryanodine receptor; HRC, histidine-rich calcium-binding protein; FAM20C, FAM20C Golgi associated secretory pathway kinase; KCHIP2, potassium voltage-gated channel-interacting protein 2; Kv4, voltage-gated potassium channel subunit Kv4; DPLP, dipeptidyl peptidase–like protein. The arrow indicates the Ca^2+^ flux. **(B)** Gene network regulated by the increased [Ca^2+^]_i_. Red represents the upregulated genes or the proteins encoded by the upregulated genes; blue represents the downregulated genes or the proteins encoded by the downregulated genes.

**Figure 6:**
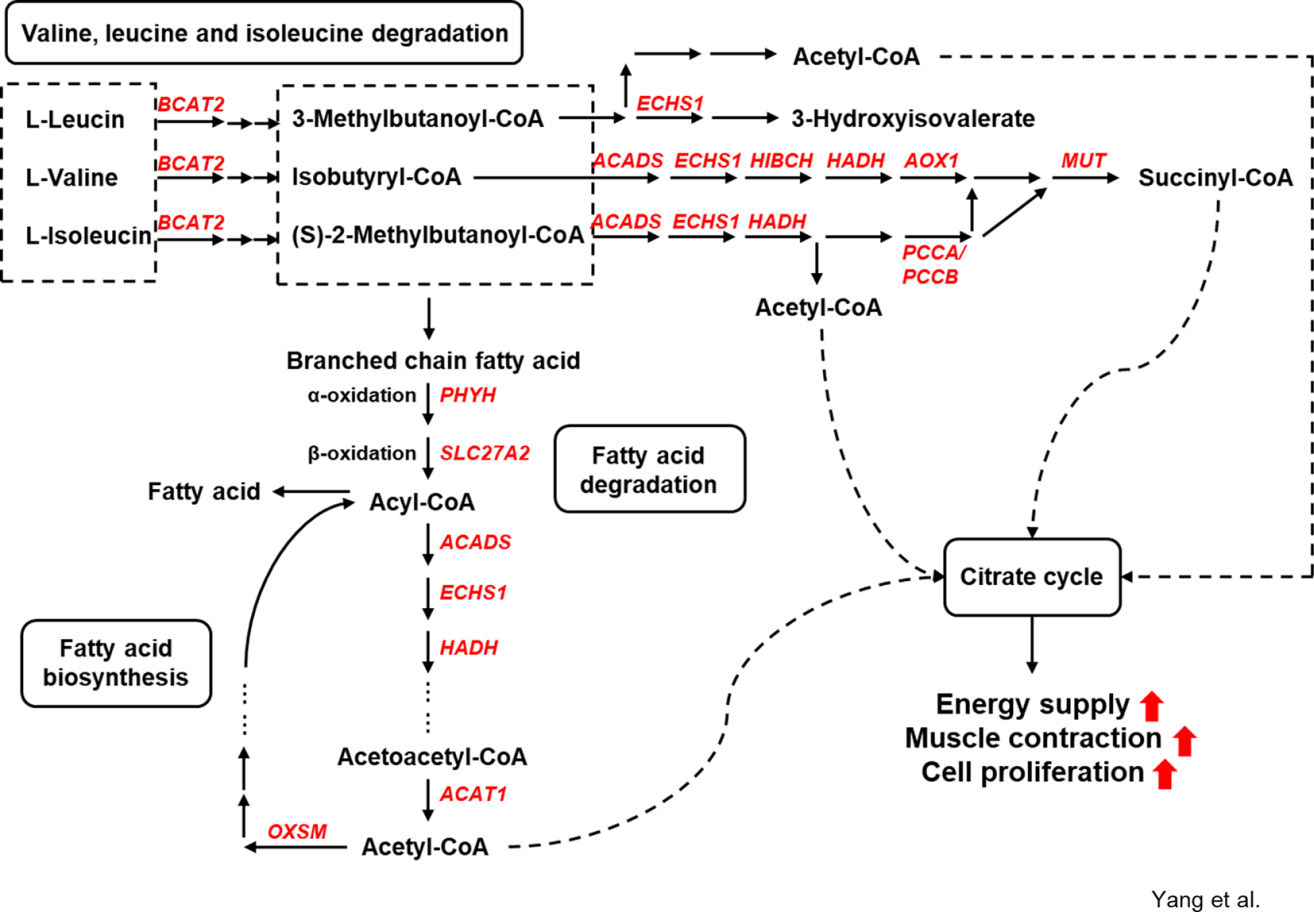
Genes involved in amino acid and fatty acid metabolism under alfalfa intervention during the preweaning period.

The relevant signaling pathways and possible functions of first neighbors in the KEGG annotation were checked to assess the coexpression network of the annotated DE genes. Two major types of regulatory networks were observed: the regulatory network of intracellular calcium concentration (**Figure 5**) and the metabolic network of amino acids and fatty acids (**Figure 6**). Other possible regulatory networks involved in muscle growth are listed in **Figure S6**.

#### The Calcium Signaling Pathway Is Differentially Expressed in Preweaning Rumen

The upregulated genes involved in membrane depolarization and muscle tissue development, including *CAV3*, L-type voltage-dependent calcium channel alpha 1D subunit (*CACNA1D*), *STAC*, *GJA1*, potassium voltage-gated channel-interacting protein 2 (*KCNIP2*), *RORA*, *PPARGC1A*, *NOS2*, and *PRKCB*, and the downregulated gene family with sequence similarity 20 member C (*FAM20C*) in the S-ALF group were coexpressed in the regulatory network of intracellular calcium concentration ([Ca^2+^]_i_). This coexpression in turn could lead to the regulation of cell proliferation and differentiation, muscle contraction and relaxation, energy metabolism, mitochondrial biogenesis, and muscle tissue remodeling.

#### Amino Acid and Fatty Acid Metabolism Are Upregulated in the Preweaning Rumen

The upregulated genes branched-chain amino acid transaminase 2 (*BCAT2*), enoyl-CoA hydratase (*ECHS1*), acyl-CoA dehydrogenase (*ACADS*), 3-hydroxyisobutyryl-CoA hydrolase (*HIBCH*), hydroxyacyl-CoA dehydrogenase (*HADH*), aldehyde oxidase 1 (*AOX1*), methylmalonyl-CoA mutase (*MUT*), and propionyl-CoA carboxylase α/β polypeptide (*PCCA*/*PCCB*) in the S-ALF group were coexpressed in the amino acid metabolic network. The upregulated genes phytanoyl-CoA 2-hydroxylase (*PHYH*), solute carrier family 27 member 2 (*SLC27A2*), acetyl-CoA acetyltransferase 1 (*ACAT1*), and 3-oxoacyl-ACP synthase (*OXSM*) in the S-ALF group were coexpressed in the metabolism network of fatty acid degradation and biosynthesis.

### qPCR Validation of Selected Genes

As shown in **Table 1**, the relative expression levels of *GPNMB*, *PDK4*, *mTOR*, *IDH2*, *NME4*, *ATF4*, *CAST*, and *APOD* in the STA group were highly consistent between RNA sequencing and qPCR. The relative expression of *GPNMB*, *MAPK6*, *LPL*, *IDH2*, *ATF4*, and *APOD* in the S-ALF group detected by qPCR was more sensitive than by RNA sequencing. The relative expression of *GJA1*, *CAV3*, *NOS2*, *STAC*, *PPARGC1A*, and *PRKCB* in the regulatory network of intracellular calcium concentration was consistent between RNA sequencing and qPCR, belonging to the U-N pattern in the S-ALF group, while *RORA* and *SMYD1*, which were part of the U-N pattern in the S-ALF group by RNA sequencing, did not significantly increase during the preweaning period by qPCR. A high correlation was observed between the mRNA expression level assessed by RNA sequencing and qPCR (*P* <0.001, *R* = 0.794; **Figure S7**).

**Table 1:** The relative gene expression of selected genes validated by quantitative PCR (qPCR) in the rumen tissue of lambs fed with milk only (B-10), milk and starter diet with alfalfa supplementation (S-ALF), or milk and starter diet without alfalfa supplementation (STA).

Gene	Groups					SEM	*P*-value^1^			
	B-10	STA-38	STA-45	S-ALF-38	S-ALF-45		S38 vs B	S45 vs S38	A38 vs B	A45 vs A38
*GPNMB* (E-3)	6.10	2.24	11.69	5.18	4.51	1.325	0.027^D^	0.043^U^	0.462^NS^	0.564^N^
*PDK4* (E-2)	8.62	2.80	8.47	6.23	5.26	1.041	0.050^D^	0.021^U^	1.000^N^	0.149^N^
*mTOR* (E-2)	2.47	2.74	2.62	2.63	3.17	0.223	0.704^N^	0.899^N^	0.825^N^	0.426^N^
*MAPK6* (E-2)	3.24	10.52	9.46	9.50	6.08	1.096	0.084^NS^	0.816^N^	0.024^N^	0.266^N^
*LPL* (E-2)	4.04	2.61	2.77	1.93	9.73	0.933	0.142^NS^	0.773^N^	0.027^NS^	0.043^NS^
*IDH2*	0.15	0.21	0.26	0.28	0.38	0.025	0.201^N^	0.485^N^	0.017^N^	0.142^N^
*NME4*	0.12	0.16	0.18	0.20	0.22	0.016	0.334^N^	0.798^N^	0.07^N^	0.761^N^
*ATF4*	0.84	1.47	1.58	1.70	1.99	0.147	0.118^N^	0.846^N^	0.023^N^	0.517^N^
*CAST*	0.28	0.32	0.35	0.30	0.48	0.037	1.000^N^	0.564^N^	0.806^N^	0.248^N^
*APOD*	0.23	0.18	0.23	0.22	0.38	0.026	0.462^N^	0.248^N^	0.462^N^	0.043^N^
*GJA1*	0.08	0.22	0.49	0.25	0.18	0.040	0.014^NS^	0.149^N^	0.050^U^	0.248^N^
*CAV3* (E-3)	0.24	1.22	2.55	2.92	4.80	0.417	0.058^NS^	0.151^N^	> 0.001^U^	0.118^N^
*NOS2* (E-2)	0.54	1.41	2.55	2.18	1.45	0.267	0.014^NS^	0.564^N^	0.014^U^	0.248^N^
*RORA* (E-2)	1.88	1.77	2.89	1.91	2.02	0.248	0.917^NS^	0.077^N^	0.980^U^	0.840^N^
*STAC* (E-2)	0.46	0.70	1.25	1.21	1.11	0.100	0.225^N^	0.141^N^	0.003^U^	0.692^N^
*PPARGC1A* (E-2)	1.20	1.87	5.69	2.66	5.43	0.560	0.360^N^	0.053^U^	0.035^U^	0.119^N^
*PRKCB*	0.05	0.18	0.35	0.29	0.38	0.043	0.086^N^	0.149^N^	0.014^U^	0.386^N^
*SMYD1* (E-2)	1.47	2.42	3.92	3.39	3.87	0.358	0.411^NS^	0.163^N^	0.144^U^	0.617^N^

^1^B = B-10, S38 = STA-38, S45 = STA-45, A38 = S-ALF-38, A45 = S-ALF-45.

^U,D,NS,and N^ Represent the upregulated (with FDR ≤0.05, |log_2_fold change| ≥1, and their expressions of all group members within one group consistently numerically higher than the other group), downregulated (with FDR ≤0.05, |log_2_fold change| ≥1, and their expressions of all group members within one group consistently numerically lower than the other group), uncertain (with FDR ≤0.05 and |log_2_fold change| ≥1 but their expressions of the group members within one group not consistently numerically higher or lower than the other group), and unchanged (with FDR >0.05 or |log_2_fold change| < 1) genes of each comparison tested by RNA sequencing. P-values without subscript represent nondifferentially expressed genes of each comparison tested by RNA sequencing.

## Discussion

Studies comparing milk only versus milk supplemented with grain or hay have demonstrated that concentrates promote the development of rumen papillae, such as by increasing their length (Connor et al., 2013), driven by the expression of key regulatory genes, such as *TFGB1*, *FBOX1*, and *PPARA* (Connor et al., 2014). Forages primarily stimulate rumen muscularization development and increase rumen volume (Zitnan et al., 1998), though the genes underlying these effects are undiscovered. The feeding strategy of milk supplemented only with hay does not exist in farming, so in contrast to these studies, we wanted to investigate what would happen in the rumen tissue, not just the rumen epithelium, when the chopped alfalfa was additionally fed to ruminants after short-chain fatty acid supplementation from the concentration starter. Our results will support roughage feeding systems with a physiological interpretation and help to identify potential commercial additives.

To our knowledge, this is the first study reporting the transcriptome profiles of rumen tissue development as affected by alfalfa. In contrast to the model of milk supplemented with hay, milk supplemented with alfalfa and starter had higher rumen papillar length and width (Yang et al., 2015), and the muscular thickness was also higher compared to the milk-supplemented starter feeding program (**Table 1**). Our transcriptomic results showed that with solid feed intake, metabolic processes, including catabolic and biosynthetic processes, were increased in rumen tissue, and these processes were further increased with alfalfa intervention, though these metabolic processes were required to be proved with additional experiments. Like fatty acid and amino acid metabolism, the pteridine-containing compound and homocysteine metabolic process were enriched by the specifically upregulated genes in the S-ALF group. Pteridine is an aromatic chemical compound composed of fused pyrimidine and pyrazine rings. It is an effective component of folic acid and lactoflavin, most often mentioned alongside NADPH, nitric oxide (NO), tyrosine, tryptophan, dopamine, phenylalanine, and glutamic acid in the literature. Folate metabolism produces one carbon unit (Morscher et al., 2018) and generates the reducing power to stimulate cell proliferation (Fan et al., 2014). Lactoflavin, which can be derived by flavinogenic microorganisms in the ruminant (Bacher et al., 2000), is a cofactor of flavin mononucleotide and of FAD that takes part in the biological oxidation processes, mitochondrial oxidative phosphorylation, and the synthesis of adenosine triphosphate (ATP) (Csapó et al., 2017). Homocysteine regulates endothelin type A receptor expression *via* the Sirtuin 1/extracellular signal–regulated kinase 1 and 2 signaling pathway for vasoconstriction in vascular smooth muscle cells (Chen et al., 2017). Future feeding trials are warranted to test the potential value of these pteridine-containing compounds and homocysteine in rumen development.

Energy and protein supply and turnover enable cell proliferation and differentiation. The skeletal muscle is the initial site of most branched-chain amino acid (BCAA, including Leu, Ile, and Val) catabolism (Holecek, 2018). Thirteen upregulated genes were annotated in Val, Leu, and Ile degradation and branched-chain fatty acid metabolism in the S-ALF group, starting with *BCAT2*, which transforms the BCAA amino group to α-ketoglutarate, and ending with *ACAT1* and *MUT*, which produce acetyl-CoA and succinyl-CoA. These products were the substrates of the citric acid cycle, which generates energy-containing compounds, including NADH, FADH, and ATP (Lieberman and Marks, 2009), that are responsible for the activation of some biological processes, such as stimulation of protein kinase C, Ca^2+^ flux, and phosphatidyl inositol metabolism, and might further promote muscle contraction (Davies, 1963) and proliferation (Wang et al., 1992).

Supplementary alfalfa not only upregulates genes involved in metabolic processes but also changes genes involved in membrane depolarization, muscle tissue development, and hypoxic response. These biological processes are all linked to calcium signaling transduction. Alfalfa is a forage with less physically effective neutral detergent fiber but higher crude protein and non-fiber carbohydrates than other forages, such as corn stover and Chinese wild rye grass (Zhu et al., 2013). In the lactating dairy cows fed with diets containing similar crude protein content, Wang et al. (2017) found that diets with alfalfa upregulated genes of rumen epithelium associated with ion-binding function, proliferation, and apoptotic processes, and complement activation compared to corn stover. Even the effects of diets containing rice straw or core stover, which were both low-nutritional-quality forage with different particle size, were different on rumen transcriptome (Wang et al., 2017). Their results suggested that both nutrients and particle sizes could alter gene expression of rumen epithelium. The promotion effects in our study are only triggered by alfalfa, or they could also be mediated by other forages fed in a similar way to the lambs, should be tested with additional experiments. In our study, supplementary alfalfa increased the *CACNA1D* gene, encoding the L-type Ca^2+^ channel subunit (Cav1.3) (Samak et al., 2011); the *GJA1* gene, encoding connexin 43 (Toyofuku et al., 1998); and the *KCNIP2* gene, encoding voltage-gated potassium (Kv) channel-interacting calcium-binding protein (Thomsen et al., 2009), changes that could lead to the influx of extracellular Ca^2+^ by opening the channel or binding to the channel to increase Ca^2+^ current. Consistent with those findings, the upregulated *STAC* gene, encoding the SH3 and cysteine-rich domain-containing protein, can suppress the Ca^2+^-dependent inactivation of L-type Ca^2+^ channels and lead to the increase of [Ca^2+^]_i_ (Polster et al., 2018). In skeletal muscle, synchronous Ca^2+^ channel opening is achieved through membrane depolarization, which leads the ryanodine receptor in the sarcoplasmic reticulum to sense the conformational change of the dihydropyridine receptors (normally the voltage-dependent calcium channels) located on the t-tubule, allowing Ca^2+^ in the sarcoplasmic reticulum to be released into the cytoplasm (Reddish et al., 2017). Cav1.3 is located on the t-tubule. Although the differential expression of the ryanodine receptor was not observed in our alfalfa-supplemented lambs, the downregulation of the *FAM20C* gene, encoding Golgi serine/threonine protein kinase, can result in Ca^2+^ release from sarcoplasmic reticulum (Pollak et al., 2017), which is consistent with these upregulated channel proteins and channel-binding proteins, suggesting that supplementary alfalfa increased [Ca^2+^]_i_. The proteins encoded by the upregulated *CAV3* gene and *KCNIP2* gene can maintain calcium rhythms (Kamishima et al., 2007; Neant et al., 2018) to keep Ca^2+^ transient. The increased [Ca^2+^]_i_ would be a signal for downstream muscle contraction (Ebashi and Endo, 1968), remodeling of muscle tissue (Trian et al., 2007), cell differentiation (Linta et al., 2013), and skin repair (Zhang and Cui, 2017). In our experiment, the downstream *PRKCB*, *NOS2*, and *PPARGC1A* genes were confirmed to be upregulated in alfalfa-supplemented lambs by qPCR. *PRKCB* encodes a Ca^2+^-activated and phospholipid-dependent protein kinase C (PKCβ). It was reported to mediate endothelial NO synthesis, promote vasodilation in the blood vessel (Wang et al., 2015), and promote cell proliferation and differentiation in smooth muscle by regulating downstream mitogen-activated protein kinase (MAPK) signaling, such as extracellular signal–regulated kinase activation (Hershenson et al., 1997). PKCα/β has also been reported to play roles in Ca^2+^-induced differentiation in the normal human epidermal keratinocyte (Matsui et al., 1992). The *PPARGC1A* gene encodes a transcriptional coactivator that regulates the genes involved in energy metabolism (Liang and Ward, 2006), and this protein is the master regulator of mitochondrial biogenesis and can promote the remodeling of muscle tissue (Handschin and Spiegelman, 2008). The expression of *eNOS* and the synthesis of NO can promote cell proliferation in vascular endothelial cells (Ou et al., 2013) or regulate contractile function and energy production in myocardial and skeletal muscle (Kobzik et al., 1994; Buchwalow et al., 2001). It was reported that AMP-activated protein kinase (AMPK) may play a role in Ca^2+^-mediated signal transduction pathways (Woods et al., 2005), and the activation of AMPK enhances the expression of *PPARGC1A* and *NOS2* in vascular smooth muscle cells (Kim et al., 2007; Ou et al., 2013). The expression of MAPK and AMPK signaling pathway members needs to be determined in the future.

Our results are transcriptomic data of rumen tissue, with information on both rumen muscle cells and epithelium cells. The epidermis of the rumen is a stratified squamous avascular tissue composed of stratum basale, stratum spinosum, stratum granulosum, and stratum corneum. Keratinocytes in the stratum basale are critically involved in maintaining epidermal thickness against environmental stresses by microbial, chemical, and physical factors. Keratinocyte differentiation progresses across the epidermis toward the stratum corneum in the presence of an extracellular calcium gradient (Eckert, 1989; Menon et al., 1992), while the life cycle of proliferative keratinocytes is associated with oxygen level (Ruthenborg et al., 2014). “Response to hypoxia” was enriched by alfalfa intervention, specifically the upregulation of the gene family with sequence similarity 162 member A (*FAM162A*), *PRKCB*, *NOS2*, *RORA*, pyruvate dehydrogenase kinase 3 (*PDK3*), and egl-9 family hypoxia-inducible factor 1 (*EGLN1*), while the genes metallothionein 3 (*MT3*), matrix metallopeptidase 2 (*MMP2*), aryl hydrocarbon receptor nuclear translocator 2 (*ARNT2*), neuron derived neurotrophic factor (*NDNF*), angiopoietin 4 (*ANGPT4*), and transforming growth factor β3 (*TGFB3*) were downregulated. The *NOS2* and *RORA* genes were both annotated in the Ca^2+^ network and “response to hypoxia.” Transcription of the *RORA* gene was upregulated by hypoxia in a panel of cell lines (Chauvet et al., 2004). It has been confirmed that *RORA* is a node in the keratinocyte differentiation network mediated partly by *FOXN1*, which is a member of the forkhead transcription factor family (Dai et al., 2013). With the inconsistent *RORA* expression between our transcriptomic and qPCR results, the rumen muscle and epithelial layer need to be further assayed with western blots separately. The transforming growth factor β (TGFβ) superfamily is critical in wound healing and repair, but it must be activated by release from the extracellular matrix, where it is bound by latent TGFβ-binding proteins, by active proteases such as the matrix metalloproteinases (Tatti et al., 2008). TGFβ has been shown to be mitogenic for fibroblasts but inhibits the proliferation of keratinocytes (Werner and Grose, 2003), and enhanced proliferation of keratinocytes is associated with reduced TGFβ activity (Kalucka et al., 2013). The downregulation of the genes *TGFB3* and *MMP2* was consistent in the S-ALF rumen tissue and, together with the increased *GJA1* gene expression, showed evidence for the proliferation and migration of keratinocytes and fibroblasts (Zhang and Cui, 2017). *EGLN1*, also known as hypoxia-inducible factor prolyl hydroxylase 2 (*PHD2*), serves as a crucial oxygen sensor and plays an important role during reepithelialization (Kalucka et al., 2013). It was found that the deletion of *PHD2* in keratinocytes enhanced migration of the hyperproliferating epithelium, which was directly related to their attenuated TGFβ and the regulation by the induction of β3-integrin in an HIF1α-dependent manner. The gene encoding HIF1α was not increased in S-ALF rumen tissue, which might be the reason for the inconsistent expression of *EGLN1* and *TGFB3*.

Calcium signaling pathways are known to be involved in the regulation of human skin development, particularly in keratinocyte development. Nevertheless, calcium signaling pathways have not yet been shown to be differentially regulated during early rumen muscle and epithelium development, or under exogenous fiber intervention. Our transcriptomic data suggest that milk supplementation with alfalfa and concentrate starter is better than milk supplemented with concentrate for promoting early rumen development, with more enhanced metabolic processes, especially calcium transduction in rumen tissue. A better understanding of the calcium signal transduction and decoding response to the changes in dietary metabolites and microorganisms may facilitate the study of the interaction between rumen microbiota and rumen development. Pteridine-containing compounds, such as folate and lactoflavin, and homocysteine might be promising additives to promote rumen development precisely.

## Data Availability Statement

The datasets generated for this study can be found in the Sequence Read Archive, access number PRJNA540396.

## Ethics Statement

The animal study was reviewed and approved by Animal Care Committee of Zhejiang University.

## Author Contributions

JW and BY designed the study and drafted the manuscript. BY, BH, SW, and YL carried out the feeding trial and collected rumen tissue samples. BY extracted the rumen tissue RNA and contributed to the data analysis. BY, HC, and JC contributed to the qPCR validation. All of the authors read and approved the final manuscript.

## Funding

This experiment was funded by the National Natural Science Foundation of China (Grant No. 31572431 and 31622056) and the National Key Research and Development Program of China (Grant No. 2017YFD0500502). The animal experiment was funded by 31572431. All the funding provided the costs of RNA sequencing, data analysis, qPCR validation, and open access publication.

## Conflict of Interest

The authors declare that the research was conducted in the absence of any commercial or financial relationships that could be construed as a potential conflict of interest.
