# Supplementary material for: Transcriptome Analysis Reveals That Alfalfa Promotes Rumen Development Through Enhanced Metabolic Processes and Calcium Transduction in Hu Lambs
Source: Front Genet. 2019 Oct 3;10:929. doi: 10.3389/fgene.2019.00929 (PMC6785638; doi:10.3389/fgene.2019.00929)
Supplement: Supplementary file 1 [file Table_1.docx]

**TABLE S1|** Information on the primers used for gene expression validation**.**

| Target genes | Primer sequence | | Amplicon length (bp) | Source^1^ |
| --- | --- | --- | --- | --- |
| *GPNMB* | *F* | AGATCACCCAGAACCCAGTC | 97 | (Zhang et al., 2011) |
|  | *R* | TATGTCCCAGACCCACTGAA |  |  |
| *PDK4* | *F* | TGGTGTTCCCCTGAGAGTCA | 109 | (Xerxa et al., 2016) |
|  | *R* | GTAACCAAAACCAGCCAGCG |  |  |
| *mTOR* | *F* | TGACCATCCTCTGCCAACAGTTCA | 122 | (Lie et al., 2013) |
|  | *R* | GCTGCATGGTCTGAACAAAGTGCT |  |  |
| *MAPK6* | *F* | TCAAAGTCAGTAAGCCGAGAA | 115 | (Fleming-Waddell et al., 2009) |
|  | *R* | AACAGTCCTCCCCACCAC |  |  |
| *LPL* | *F* | TCACGTATGAAGCCCCACAT | 119 | (Fleming-Waddell et al., 2009) |
|  | *R* | AAGGAGTGTTCCGGCACCA |  |  |
| *IDH2* | *F* | CTGGACGCGTGGCCTAGAACA | 150 | (Fleming-Waddell et al., 2009) |
|  | *R* | TTGCTGAGGCCGTGGATGC |  |  |
| *NME4* | *F* | CACCTCGAGGGCCATGATAG | 98 | (Fleming-Waddell et al., 2009) |
|  | *R* | TGGATGACATTCCTGCTGACG |  |  |
| *ATF4* | *F* | AGAGAGGAGTCAGGGCTCATA | 178 | (Fleming-Waddell et al., 2009) |
|  | *R* | TGTCTTCCACTCCAGATCATTC |  |  |
| *CAST* | *F* | AGAGACCCACCAGGACGTG | 197 | (Fleming-Waddell et al., 2009) |
|  | *R* | CAGGGGCTCTAGGCTAACA |  |  |
| *APOD* | *F* | TCTTGCTTTGCTTTTCCCCTATACC | 128 | (Fleming-Waddell et al., 2009) |
|  | *R* | AGCTTGCCTTGGGTTCTTCTCC |  |  |
| *GJA1* | *F* | CAAACAAGCAAGTGAGCAAAAC | 116 | qPrimerDB (O.aries.018781v1) |
|  | *R* | GGTCATCTGGGAAATCAAAAGG |  |  |
| *CAV3* | *F* | GTCTCCAAGTACTGGTGCTAC | 144 | qPrimerDB (O.aries.007012v1) |
|  | *R* | GATCTCGATCAGGTAGCTCTTG |  |  |
| *NOS2* | *F* | TCAGAGCCACGATCCTCTTT | 475 | (Kim et al., 2011) |
|  | *R* | GGGATCTCAATGTGGTGCTT |  |  |
| *RORA* | *F* | TGTTTGATTGATCGGACCAGTA | 120 | qPrimerDB (O.aries.019586v1) |
|  | *R* | GTCTCTCTGCTTTTTCGACATG |  |  |
| *SMYD1* | *F* | CTTATTCCGCTGTGGTTTTTGA | 176 | qPrimerDB (O.aries.019523v1) |
|  | *R* | ATATCTCTTGATGGCCGAACAT |  |  |
| *STAC* | *F* | CAAGAGCACATCTTCAAGAAGC | 117 | qPrimerDB (O.aries.015484v1) |
|  | *R* | CTTGTGGTGGATGCTCATCTTA |  |  |
| *PPARGC1A* | *F* | CCCACAGAGAACCGGAACAG | 285 | This study |
|  | *R* | AGAGGGCTTCAGCTTTGGAG |  |  |
| *PRKCB* | *F* | CATACTCCAGCCCCACGTTT | 307 | This study |
|  | *R* | TGCTTGCTCTCACTCTTGGG |  |  |
| *GAPDH* | *F* | GGGTCATCATCTCTGCACCT | 176 | (Wang et al., 2009) |
|  | *R* | GGTCATAAGTCCCTCCACGA |  |  |

^1^qPrimerDB = qPCR Primer Database (https://biodb.swu.edu.cn/qprimerdb/); the primer ID in the qPrimerDB was provide in the bracket.

**References**

Fleming-Waddell, J.N., Olbricht, G.R., Taxis, T.M., White, J.D., Vuocolo, T., Craig, B.A., et al. (2009). Effect of DLK1 and RTL1 but not MEG3 or MEG8 on muscle gene expression in Callipyge lambs. *PLoS One* 4(10)**,** e7399. doi: 10.1371/journal.pone.0007399.

Kim, J., Burghardt, R.C., Wu, G., Johnson, G.A., Spencer, T.E., and Bazer, F.W. (2011). Select nutrients in the ovine uterine lumen. IX. Differential effects of arginine, leucine, glutamine, and glucose on interferon tau, ornithine decarboxylase, and nitric oxide synthase in the ovine conceptus. *Biol Reprod* 84(6)**,** 1139-1147. doi: 10.1095/biolreprod.110.088153.

Lie, S., Sim, S.M., McMillen, I.C., Williams-Wyss, O., MacLaughlin, S.M., Kleemann, D.O., et al. (2013). Maternal undernutrition around the time of conception and embryo number each impact on the abundance of key regulators of cardiac growth and metabolism in the fetal sheep heart. *J Dev Orig Health Dis* 4(5)**,** 377-390. doi: 10.1017/s2040174413000354.

Wang, A., Gu, Z., Heid, B., Akers, R.M., and Jiang, H. (2009). Identification and characterization of the bovine G protein-coupled receptor GPR41 and GPR43 genes. *J Dairy Sci* 92(6)**,** 2696-2705. doi: 10.3168/jds.2009-2037.

Xerxa, E., Barbisin, M., Chieppa, M.N., Krmac, H., Costassa, E.V., Vatta, P., et al. (2016). Whole Blood Gene Expression Profiling in Preclinical and Clinical Cattle Infected with Atypical Bovine Spongiform Encephalopathy. *PLos One* 11(4)**,** e0153425. doi: 10.1371/journal.pone.0153425.

Zhang, Y.Y., Zan, L.S., Wang, H.B., Qing, L., Wu, K.X., Quan, S.A., et al. (2011). Differentially expressed genes in skeletal muscle tissues from castrated Qinchuan cattle males compared with those from intact males. *Livest Sci* 135(1)**,** 76-83. doi: 10.1016/j.livsci.2010.06.070.
