# Supplementary material for: Transcriptome Analysis Reveals That Alfalfa Promotes Rumen Development Through Enhanced Metabolic Processes and Calcium Transduction in Hu Lambs
Source: Front Genet. 2019 Oct 3;10:929. doi: 10.3389/fgene.2019.00929 (PMC6785638; doi:10.3389/fgene.2019.00929)
Supplement: Supplementary file 2 [file Table_2.docx]

**TABLE S2|** Rumen epithelial and muscular thickness (mean ± SD, μm) of Hu lambs fed with milk or the starter diet with (S-ALF) or without alfalfa intervention (STA) at different ages.

|  | STA | |  | S-ALF | |  | STA vs. S-ALF^1^ | |
| --- | --- | --- | --- | --- | --- | --- | --- | --- |
| Age (d) | epithelium | muscle |  | epithelium | muscle |  | epithelium | muscle |
| 10 | 79.37±9.28^ab^ | 326±48 |  | 79.37±9.28^ab^ | 326±48^a^ |  | - | - |
| 17 | 90.24±20.97^ab^ | 473±172 |  | 70.85±6.10^a^ | 404±120^ab^ |  | 0.079 | 0.441 |
| 24 | 80.45±10.46^ab^ | 533±56 |  | 95.19±12.14^b^ | 570±103^abc^ |  | 0.154 | 0.609 |
| 38 | 95.50±15.32^b^ | 414±88 |  | 98.26±12.54^b^ | 772±154^c^ |  | 0.755 | 0.001 |
| 45 | 63.97±8.48^a^ | 588±275 |  | 68.07±10.96^a^ | 699±248^c^ |  | 0.485 | 0.478 |
| 66 | 69.69±13.46^ab^ | 659±292 |  | 71.03±7.81^a^ | 636±135^bc^ |  | 0.850 | 0.878 |
| Age effect |  |  |  |  |  |  |  |  |
| From d10 to 66 | 0.005 | 0.073 |  | <0.001 | <0.001 |  | - | - |

^a-c^Means within a column with different superscripts differ (*P*-value ≤0.05). ^1^*P*-value of the comparison between STA and S-ALF group.
