# Supplementary material for: Transcriptome Analysis Reveals That Alfalfa Promotes Rumen Development Through Enhanced Metabolic Processes and Calcium Transduction in Hu Lambs
Source: Front Genet. 2019 Oct 3;10:929. doi: 10.3389/fgene.2019.00929 (PMC6785638; doi:10.3389/fgene.2019.00929)
Supplement: Supplementary file 3 [file Table_3.docx]

**TABLE S3|** Number of reads generated for 23 Hu lambs by RNA-sequencing.

| Groups | Mean | Minimum | Maximum | Standard deviation |
| --- | --- | --- | --- | --- |
| B-10 | 24446581 | 21549608 | 26415115 | 2109984 |
| STA-38 | 24078161 | 20575226 | 30538714 | 4154177 |
| STA-45 | 23654425 | 20090214 | 29997039 | 4104558 |
| S-ALF-38 | 20322255 | 19458605 | 22324906 | 1342454 |
| S-ALF-45 | 23394093 | 22114122 | 24738115 | 1267492 |
| All | 23293966 | 19458605 | 30538714 | 3090926 |
