# Supplementary material for: Transcriptome Analysis Reveals That Alfalfa Promotes Rumen Development Through Enhanced Metabolic Processes and Calcium Transduction in Hu Lambs
Source: Front Genet. 2019 Oct 3;10:929. doi: 10.3389/fgene.2019.00929 (PMC6785638; doi:10.3389/fgene.2019.00929)
Supplement: Supplementary file 4 [file Table_4.docx]

**TABLE S4|** The GO terms enriched by the genes of the N-N pattern of Hu lambs fed with milk or the starter diet with (S-ALF) or without alfalfa supplementation (STA).

|  |  | STA | |  | S-ALF | |
| --- | --- | --- | --- | --- | --- | --- |
| Category | Term | Count | *P*-value |  | Count | *P*-value |
| GOTERM_BP_FAT | GO:0010467~gene expression | 1894 | 2.6301E-15 |  | 1879 | 6.2529E-15 |
| GOTERM_BP_FAT | GO:0034645~cellular macromolecule biosynthetic process | 1753 | 2.3855E-12 |  | 1737 | 9.0126E-12 |
| GOTERM_BP_FAT | GO:0016070~RNA metabolic process | 1555 | 3.7977E-15 |  | 1544 | 4.9807E-15 |
| GOTERM_BP_FAT | GO:0051171~regulation of nitrogen compound metabolic process | 1407 | 1.3641E-06 |  | 1401 | 5.092E-07 |
| GOTERM_BP_FAT | GO:0010468~regulation of gene expression | 1390 | 9.4172E-06 |  | 1379 | 1.2266E-05 |
| GOTERM_BP_FAT | GO:0019438~aromatic compound biosynthetic process | 1341 | 0.00635846 |  | 1331 | 0.00644637 |
| GOTERM_BP_FAT | GO:0018130~heterocycle biosynthetic process | 1339 | 0.00588195 |  | 1329 | 0.00600086 |
| GOTERM_BP_FAT | GO:0034654~nucleobase-containing compound biosynthetic process | 1318 | 0.00332756 |  | 1308 | 0.0035492 |
| GOTERM_BP_FAT | GO:0036211~protein modification process | 1305 | 9.3874E-19 |  | 1295 | 2.106E-18 |
| GOTERM_BP_FAT | GO:0006464~cellular protein modification process | 1305 | 9.3874E-19 |  | 1295 | 2.106E-18 |
| GOTERM_BP_FAT | GO:0019219~regulation of nucleobase-containing compound metabolic process | 1303 | 2.2156E-05 |  | 1295 | 1.6781E-05 |
| GOTERM_BP_FAT | GO:0010556~regulation of macromolecule biosynthetic process | 1299 | 3.1976E-06 |  | 1291 | 2.3374E-06 |
| GOTERM_BP_FAT | GO:2000112~regulation of cellular macromolecule biosynthetic process | 1260 | 9.4239E-07 |  | 1253 | 5.8663E-07 |
| GOTERM_BP_FAT | GO:0032774~RNA biosynthetic process | 1191 | 0.00033925 |  | 1180 | 0.00054808 |
| GOTERM_BP_FAT | GO:0097659~nucleic acid-templated transcription | 1182 | 0.00056344 |  | 1172 | 0.00074435 |
| GOTERM_BP_FAT | GO:0051252~regulation of RNA metabolic process | 1149 | 8.4433E-05 |  | 1141 | 8.0745E-05 |
| GOTERM_BP_FAT | GO:0009893~positive regulation of metabolic process | 1137 | 0.00109209 |  | 1125 | 0.00213365 |
| GOTERM_BP_FAT | GO:0035556~intracellular signal transduction | 1090 | 2.3573E-07 |  | 1084 | 1.4864E-07 |
| GOTERM_BP_FAT | GO:0010604~positive regulation of macromolecule metabolic process | 1082 | 5.0302E-05 |  | 1071 | 0.00010842 |
| GOTERM_BP_FAT | GO:2001141~regulation of RNA biosynthetic process | 1082 | 0.00123495 |  | 1076 | 0.00089806 |
| GOTERM_BP_FAT | GO:1903506~regulation of nucleic acid-templated transcription | 1079 | 0.00121527 |  | 1073 | 0.00086905 |
| GOTERM_BP_FAT | GO:0006355~regulation of transcription, DNA-templated | 1077 | 0.00141936 |  | 1071 | 0.00100098 |
| GOTERM_BP_FAT | GO:0009966~regulation of signal transduction | 1063 | 0.00309796 |  | 1063 | 0.00074601 |
| GOTERM_BP_FAT | GO:0033036~macromolecule localization | 1062 | 1.7639E-09 |  | 1053 | 3.4362E-09 |
| GOTERM_BP_FAT | GO:0044085~cellular component biogenesis | 1059 | 2.431E-11 |  | 1045 | 3.6059E-10 |
| GOTERM_BP_FAT | GO:0031325~positive regulation of cellular metabolic process | 1054 | 0.00110466 |  | 1045 | 0.00147297 |
| GOTERM_BP_FAT | GO:0006351~transcription, DNA-templated | 1032 | 3.6532E-05 |  | 1022 | 6.8666E-05 |
| GOTERM_BP_FAT | GO:0051641~cellular localization | 1026 | 7.2264E-20 |  | 1016 | 5.6066E-19 |
| GOTERM_BP_FAT | GO:0006793~phosphorus metabolic process | 997 | 0.03838166 |  | 997 | 0.0140075 |
| GOTERM_BP_FAT | GO:0006796~phosphate-containing compound metabolic process | 994 | 0.03461395 |  | 995 | 0.01081983 |
| GOTERM_BP_FAT | GO:0022607~cellular component assembly | 989 | 7.8039E-10 |  | 975 | 1.1489E-08 |
| GOTERM_BP_FAT | GO:0051246~regulation of protein metabolic process | 985 | 2.2826E-10 |  | 988 | 4.9722E-12 |
| GOTERM_BP_FAT | GO:0051128~regulation of cellular component organization | 959 | 0.00151732 |  | 941 | 0.01037257 |
| GOTERM_BP_FAT | GO:0008104~protein localization | 953 | 7.3415E-11 |  | 945 | 1.5256E-10 |
| GOTERM_BP_FAT | GO:0032268~regulation of cellular protein metabolic process | 899 | 7.9206E-10 |  | 902 | 2.1053E-11 |
| GOTERM_BP_FAT | GO:0009892~negative regulation of metabolic process | 864 | 4.0006E-07 |  | 870 | 6.5509E-09 |
| GOTERM_BP_FAT | GO:0070887~cellular response to chemical stimulus | 858 | 0.02804009 |  | 857 | 0.0128278 |
| GOTERM_BP_FAT | GO:0043933~macromolecular complex subunit organization | 843 | 1.1261E-11 |  | 833 | 8.7216E-11 |
| GOTERM_BP_FAT | GO:0031324~negative regulation of cellular metabolic process | 806 | 1.3965E-06 |  | 814 | 1.319E-08 |
| GOTERM_BP_FAT | GO:0065009~regulation of molecular function | 804 | 0.00019785 |  | 805 | 3.6447E-05 |
| GOTERM_BP_FAT | GO:0033554~cellular response to stress | 795 | 4.424E-20 |  | 791 | 2.9297E-20 |
| GOTERM_BP_FAT | GO:1902589~single-organism organelle organization | 790 | 1.095E-12 |  | 778 | 3.1231E-11 |
| GOTERM_BP_FAT | GO:0010605~negative regulation of macromolecule metabolic process | 789 | 1.0633E-08 |  | 793 | 2.5736E-10 |
| GOTERM_BP_FAT | GO:0051649~establishment of localization in cell | 750 | 1.1841E-17 |  | 748 | 2.6621E-18 |
| GOTERM_BP_FAT | GO:0045184~establishment of protein localization | 730 | 7.4793E-10 |  | 726 | 5.6354E-10 |
| GOTERM_BP_FAT | GO:0070727~cellular macromolecule localization | 722 | 4.4916E-16 |  | 717 | 7.8753E-16 |
| GOTERM_BP_FAT | GO:0034613~cellular protein localization | 718 | 7.6954E-16 |  | 713 | 1.1636E-15 |
| GOTERM_BP_FAT | GO:0016310~phosphorylation | 711 | 0.00019636 |  | 713 | 2.9627E-05 |
| GOTERM_BP_FAT | GO:0008219~cell death | 699 | 0.0153045 |  | 698 | 0.00749273 |
| GOTERM_BP_FAT | GO:0071310~cellular response to organic substance | 672 | 0.02678218 |  | 671 | 0.01413192 |
| GOTERM_BP_FAT | GO:0031399~regulation of protein modification process | 670 | 3.313E-05 |  | 672 | 4.2636E-06 |
| GOTERM_BP_FAT | GO:0012501~programmed cell death | 660 | 0.01232233 |  | 658 | 0.00744725 |
| GOTERM_BP_FAT | GO:1902531~regulation of intracellular signal transduction | 658 | 0.00026933 |  | 660 | 4.3862E-05 |
| GOTERM_BP_FAT | GO:0051173~positive regulation of nitrogen compound metabolic process | 653 | 0.00128312 |  | 649 | 0.00112874 |
| GOTERM_BP_FAT | GO:0015031~protein transport | 635 | 4.1561E-08 |  | 633 | 1.7465E-08 |
| GOTERM_BP_FAT | GO:0009891~positive regulation of biosynthetic process | 634 | 0.02319421 |  | 628 | 0.0293348 |
| GOTERM_BP_FAT | GO:0010628~positive regulation of gene expression | 623 | 0.00289461 |  | 614 | 0.00760342 |
| GOTERM_BP_FAT | GO:0045935~positive regulation of nucleobase-containing compound metabolic process | 620 | 0.00059224 |  | 614 | 0.00091196 |
| GOTERM_BP_FAT | GO:0031328~positive regulation of cellular biosynthetic process | 620 | 0.02564323 |  | 616 | 0.02375242 |
| GOTERM_BP_FAT | GO:0006915~apoptotic process | 618 | 0.02338448 |  | 617 | 0.01277085 |
| GOTERM_BP_FAT | GO:0046907~intracellular transport | 616 | 1.3587E-21 |  | 611 | 4.7629E-21 |
| GOTERM_BP_FAT | GO:0006468~protein phosphorylation | 616 | 0.00061023 |  | 613 | 0.0004427 |
| GOTERM_BP_FAT | GO:0010557~positive regulation of macromolecule biosynthetic process | 603 | 0.00061873 |  | 597 | 0.00098628 |
| GOTERM_BP_FAT | GO:0007049~cell cycle | 601 | 1.0832E-12 |  | 601 | 1.3474E-13 |
| GOTERM_BP_FAT | GO:0006366~transcription from RNA polymerase II promoter | 596 | 0.00852056 |  | 598 | 0.00226123 |
| GOTERM_BP_FAT | GO:0006357~regulation of transcription from RNA polymerase II promoter | 594 | 0.00864984 |  | 595 | 0.00288795 |
| GOTERM_BP_FAT | GO:0065003~macromolecular complex assembly | 581 | 6.1269E-08 |  | 572 | 5.3469E-07 |
| GOTERM_BP_FAT | GO:0010941~regulation of cell death | 578 | 0.03459031 |  | 580 | 0.01163601 |
| GOTERM_BP_FAT | GO:0051247~positive regulation of protein metabolic process | 571 | 0.00964829 |  | 568 | 0.00782346 |
| GOTERM_BP_FAT | GO:0044248~cellular catabolic process | 565 | 5.6331E-09 |  | 561 | 7.0053E-09 |
| GOTERM_BP_FAT | GO:0048585~negative regulation of response to stimulus | 561 | 0.01336721 |  | 564 | 0.00309019 |
| GOTERM_BP_FAT | GO:0050790~regulation of catalytic activity | 555 | 0.00088125 |  | 561 | 5.2457E-05 |
| GOTERM_BP_FAT | GO:0042325~regulation of phosphorylation | 547 | 0.01483832 |  | 551 | 0.00279012 |
| GOTERM_BP_FAT | GO:0051254~positive regulation of RNA metabolic process | 528 | 0.00058143 |  | 520 | 0.00184694 |
| GOTERM_BP_FAT | GO:0071822~protein complex subunit organization | 527 | 0.0001716 |  | 517 | 0.00106498 |
| GOTERM_BP_FAT | GO:0032270~positive regulation of cellular protein metabolic process | 525 | 0.0154501 |  | 521 | 0.01629281 |
| GOTERM_BP_FAT | GO:0051172~negative regulation of nitrogen compound metabolic process | 524 | 1.4713E-05 |  | 521 | 1.2556E-05 |
| GOTERM_BP_FAT | GO:0080134~regulation of response to stress | 523 | 0.00055015 |  | 527 | 5.6451E-05 |
| GOTERM_BP_FAT | GO:0033043~regulation of organelle organization | 513 | 1.4412E-06 |  | 509 | 1.9007E-06 |
| GOTERM_BP_FAT | GO:0007010~cytoskeleton organization | 513 | 2.4353E-06 |  | 503 | 2.8452E-05 |
| GOTERM_BP_FAT | GO:0010629~negative regulation of gene expression | 509 | 1.7748E-05 |  | 504 | 3.1599E-05 |
| GOTERM_BP_FAT | GO:0001932~regulation of protein phosphorylation | 506 | 0.01893612 |  | 508 | 0.00594988 |
| GOTERM_BP_FAT | GO:0009890~negative regulation of biosynthetic process | 504 | 0.00098259 |  | 500 | 0.00114 |
| GOTERM_BP_FAT | GO:1902680~positive regulation of RNA biosynthetic process | 504 | 0.00116977 |  | 498 | 0.00225674 |
| GOTERM_BP_FAT | GO:0006508~proteolysis | 503 | 2.802E-11 |  | 503 | 4.7938E-12 |
| GOTERM_BP_FAT | GO:0022402~cell cycle process | 503 | 6.1252E-11 |  | 504 | 5.9335E-12 |
| GOTERM_BP_FAT | GO:1903508~positive regulation of nucleic acid-templated transcription | 501 | 0.0013027 |  | 496 | 0.00195413 |
| GOTERM_BP_FAT | GO:0045893~positive regulation of transcription, DNA-templated | 501 | 0.0013027 |  | 496 | 0.00195413 |
| GOTERM_BP_FAT | GO:0031327~negative regulation of cellular biosynthetic process | 497 | 0.00078998 |  | 492 | 0.00122504 |
| GOTERM_BP_FAT | GO:0009057~macromolecule catabolic process | 488 | 1.8833E-18 |  | 487 | 5.0292E-19 |
| GOTERM_BP_FAT | GO:0016192~vesicle-mediated transport | 482 | 3.7312E-08 |  | 479 | 3.7412E-08 |
| GOTERM_BP_FAT | GO:0010558~negative regulation of macromolecule biosynthetic process | 480 | 0.00020816 |  | 476 | 0.00026549 |
| GOTERM_BP_FAT | GO:0070271~protein complex biogenesis | 465 | 0.000678 |  | 455 | 0.00423272 |
| GOTERM_BP_FAT | GO:0006461~protein complex assembly | 465 | 0.000678 |  | 455 | 0.00423272 |
| GOTERM_BP_FAT | GO:0045934~negative regulation of nucleobase-containing compound metabolic process | 464 | 0.00062048 |  | 461 | 0.00059593 |
| GOTERM_BP_FAT | GO:2000113~negative regulation of cellular macromolecule biosynthetic process | 462 | 4.9868E-05 |  | 458 | 6.9584E-05 |
| GOTERM_BP_FAT | GO:0044093~positive regulation of molecular function | 462 | 0.0262609 |  | 459 | 0.0248693 |
| GOTERM_BP_FAT | GO:0009968~negative regulation of signal transduction | 442 | 0.04327568 |  | 450 | 0.00409271 |
| GOTERM_BP_FAT | GO:0051276~chromosome organization | 433 | 1.9966E-09 |  | 434 | 2.5377E-10 |
| GOTERM_BP_FAT | GO:0006259~DNA metabolic process | 429 | 1.3417E-12 |  | 429 | 2.4814E-13 |
| GOTERM_BP_FAT | GO:0006886~intracellular protein transport | 428 | 1.0312E-12 |  | 427 | 3.8458E-13 |
| GOTERM_BP_FAT | GO:0072359~circulatory system development | 426 | 0.01374323 |  | 423 | 0.0137825 |
| GOTERM_BP_FAT | GO:0072358~cardiovascular system development | 426 | 0.01374323 |  | 423 | 0.0137825 |
| GOTERM_BP_FAT | GO:0044265~cellular macromolecule catabolic process | 408 | 3.169E-19 |  | 406 | 2.9351E-19 |
| GOTERM_BP_FAT | GO:0051253~negative regulation of RNA metabolic process | 404 | 0.00110719 |  | 401 | 0.00120207 |
| GOTERM_BP_FAT | GO:1902580~single-organism cellular localization | 395 | 3.4768E-05 |  | 391 | 6.0513E-05 |
| GOTERM_BP_FAT | GO:0033365~protein localization to organelle | 392 | 4.3586E-12 |  | 391 | 1.8645E-12 |
| GOTERM_BP_FAT | GO:0051726~regulation of cell cycle | 390 | 7.8849E-06 |  | 393 | 6.7137E-07 |
| GOTERM_BP_FAT | GO:0006974~cellular response to DNA damage stimulus | 382 | 9.6553E-20 |  | 381 | 3.9592E-20 |
| GOTERM_BP_FAT | GO:0043603~cellular amide metabolic process | 381 | 0.00025698 |  | 379 | 0.0002151 |
| GOTERM_BP_FAT | GO:1902679~negative regulation of RNA biosynthetic process | 380 | 0.00662564 |  | 377 | 0.00730125 |
| GOTERM_BP_FAT | GO:0030163~protein catabolic process | 378 | 1.2665E-15 |  | 380 | 2.2128E-17 |
| GOTERM_BP_FAT | GO:0018193~peptidyl-amino acid modification | 373 | 4.758E-05 |  | 373 | 1.8014E-05 |
| GOTERM_BP_FAT | GO:0051248~negative regulation of protein metabolic process | 372 | 3.8618E-08 |  | 381 | 4.1545E-11 |
| GOTERM_BP_FAT | GO:1903507~negative regulation of nucleic acid-templated transcription | 372 | 0.00827269 |  | 370 | 0.00716695 |
| GOTERM_BP_FAT | GO:0070647~protein modification by small protein conjugation or removal | 371 | 1.4855E-17 |  | 371 | 2.5406E-18 |
| GOTERM_BP_FAT | GO:0045892~negative regulation of transcription, DNA-templated | 371 | 0.00903547 |  | 369 | 0.0078463 |
| GOTERM_BP_FAT | GO:0060341~regulation of cellular localization | 358 | 0.00078329 |  | 359 | 0.00024608 |
| GOTERM_BP_FAT | GO:0032269~negative regulation of cellular protein metabolic process | 347 | 1.053E-08 |  | 357 | 3.3586E-12 |
| GOTERM_BP_FAT | GO:0045944~positive regulation of transcription from RNA polymerase II promoter | 345 | 0.04619385 |  | 346 | 0.0223888 |
| GOTERM_BP_FAT | GO:0000278~mitotic cell cycle | 339 | 8.8585E-14 |  | 344 | 2.4489E-16 |
| GOTERM_BP_FAT | GO:0006518~peptide metabolic process | 337 | 2.878E-05 |  | 334 | 4.1488E-05 |
| GOTERM_BP_FAT | GO:0061024~membrane organization | 336 | 0.00109182 |  | 326 | 0.01063694 |
| GOTERM_BP_FAT | GO:0034622~cellular macromolecular complex assembly | 335 | 6.1767E-07 |  | 328 | 7.1981E-06 |
| GOTERM_BP_FAT | GO:0051270~regulation of cellular component movement | 335 | 0.01884243 |  | 334 | 0.01368224 |
| GOTERM_BP_FAT | GO:0043604~amide biosynthetic process | 322 | 4.4213E-05 |  | 320 | 4.3432E-05 |
| GOTERM_BP_FAT | GO:0044257~cellular protein catabolic process | 320 | 1.1876E-14 |  | 320 | 2.5937E-15 |
| GOTERM_BP_FAT | GO:0007017~microtubule-based process | 316 | 3.2993E-05 |  | 312 | 7.7993E-05 |
| GOTERM_BP_FAT | GO:0005975~carbohydrate metabolic process | 312 | 0.01769193 |  | 308 | 0.02737982 |
| GOTERM_BP_FAT | GO:0006396~RNA processing | 311 | 5.9274E-23 |  | 312 | 2.7084E-24 |
| GOTERM_BP_FAT | GO:2000145~regulation of cell motility | 308 | 0.02133103 |  | 307 | 0.0161331 |
| GOTERM_BP_FAT | GO:0051338~regulation of transferase activity | 305 | 0.00044011 |  | 312 | 9.8705E-06 |
| GOTERM_BP_FAT | GO:0051603~proteolysis involved in cellular protein catabolic process | 302 | 2.5804E-14 |  | 302 | 6.1044E-15 |
| GOTERM_BP_FAT | GO:0043043~peptide biosynthetic process | 302 | 6.6043E-06 |  | 300 | 7.0867E-06 |
| GOTERM_BP_FAT | GO:1901701~cellular response to oxygen-containing compound | 300 | 0.02263801 |  | 302 | 0.00782682 |
| GOTERM_BP_FAT | GO:0001944~vasculature development | 296 | 0.00065577 |  | 292 | 0.00137101 |
| GOTERM_BP_FAT | GO:0032446~protein modification by small protein conjugation | 295 | 5.6005E-15 |  | 295 | 1.2762E-15 |
| GOTERM_BP_FAT | GO:0006412~translation | 295 | 5.3582E-06 |  | 293 | 5.9154E-06 |
| GOTERM_BP_FAT | GO:1903047~mitotic cell cycle process | 293 | 2.6987E-13 |  | 298 | 5.8139E-16 |
| GOTERM_BP_FAT | GO:0044802~single-organism membrane organization | 292 | 0.00718274 |  | 285 | 0.02698985 |
| GOTERM_BP_FAT | GO:0070925~organelle assembly | 289 | 3.626E-06 |  | 286 | 6.875E-06 |
| GOTERM_BP_FAT | GO:0030334~regulation of cell migration | 289 | 0.04763307 |  | 288 | 0.03799638 |
| GOTERM_BP_FAT | GO:0097190~apoptotic signaling pathway | 285 | 0.00027574 |  | 289 | 2.1645E-05 |
| GOTERM_BP_FAT | GO:0010638~positive regulation of organelle organization | 281 | 6.6557E-05 |  | 275 | 0.00039848 |
| GOTERM_BP_FAT | GO:0007005~mitochondrion organization | 279 | 0.00027808 |  | 278 | 0.00020127 |
| GOTERM_BP_FAT | GO:0030029~actin filament-based process | 279 | 0.00583304 |  | 272 | 0.02402933 |
| GOTERM_BP_FAT | GO:0001568~blood vessel development | 276 | 0.00089668 |  | 273 | 0.00138972 |
| GOTERM_BP_FAT | GO:0007264~small GTPase mediated signal transduction | 275 | 0.01063849 |  | 281 | 0.00082119 |
| GOTERM_BP_FAT | GO:0016567~protein ubiquitination | 270 | 3.7395E-14 |  | 270 | 9.8456E-15 |
| GOTERM_BP_FAT | GO:0043549~regulation of kinase activity | 266 | 0.00300889 |  | 272 | 0.00015515 |
| GOTERM_BP_FAT | GO:0051336~regulation of hydrolase activity | 262 | 0.03514607 |  | 263 | 0.01718911 |
| GOTERM_BP_FAT | GO:0080135~regulation of cellular response to stress | 260 | 1.0186E-06 |  | 259 | 7.4429E-07 |
| GOTERM_BP_FAT | GO:0006325~chromatin organization | 260 | 2.0018E-05 |  | 259 | 1.4834E-05 |
| GOTERM_BP_FAT | GO:1903827~regulation of cellular protein localization | 260 | 0.00018345 |  | 262 | 3.4377E-05 |
| GOTERM_BP_FAT | GO:0010256~endomembrane system organization | 255 | 2.1208E-07 |  | 252 | 5.5374E-07 |
| GOTERM_BP_FAT | GO:0006605~protein targeting | 255 | 7.9546E-06 |  | 254 | 5.9536E-06 |
| GOTERM_BP_FAT | GO:0043632~modification-dependent macromolecule catabolic process | 254 | 5.3848E-12 |  | 256 | 2.4281E-13 |
| GOTERM_BP_FAT | GO:0072594~establishment of protein localization to organelle | 253 | 5.1334E-08 |  | 252 | 3.8302E-08 |
| GOTERM_BP_FAT | GO:0019941~modification-dependent protein catabolic process | 250 | 1.5138E-11 |  | 252 | 7.2754E-13 |
| GOTERM_BP_FAT | GO:0006511~ubiquitin-dependent protein catabolic process | 247 | 9.0268E-12 |  | 249 | 4.159E-13 |
| GOTERM_BP_FAT | GO:0044723~single-organism carbohydrate metabolic process | 246 | 0.00330962 |  | 241 | 0.0099357 |
| GOTERM_BP_FAT | GO:0030036~actin cytoskeleton organization | 246 | 0.00516603 |  | 238 | 0.0328157 |
| GOTERM_BP_FAT | GO:1902582~single-organism intracellular transport | 240 | 8.1936E-06 |  | 239 | 6.4329E-06 |
| GOTERM_BP_FAT | GO:0045859~regulation of protein kinase activity | 240 | 0.00211366 |  | 244 | 0.00022117 |
| GOTERM_BP_FAT | GO:0006914~autophagy | 235 | 1.9685E-05 |  | 232 | 4.4951E-05 |
| GOTERM_BP_FAT | GO:0000122~negative regulation of transcription from RNA polymerase II promoter | 232 | 0.03761039 |  | 231 | 0.03224382 |
| GOTERM_BP_FAT | GO:0010564~regulation of cell cycle process | 231 | 0.00049884 |  | 232 | 0.00016558 |
| GOTERM_BP_FAT | GO:0031400~negative regulation of protein modification process | 230 | 3.2332E-05 |  | 239 | 5.7534E-08 |
| GOTERM_BP_FAT | GO:0048514~blood vessel morphogenesis | 227 | 0.00115225 |  | 223 | 0.00306677 |
| GOTERM_BP_FAT | GO:0014070~response to organic cyclic compound | 227 | 0.01769081 |  | 233 | 0.00137612 |
| GOTERM_BP_FAT | GO:0006281~DNA repair | 223 | 2.425E-11 |  | 224 | 3.0952E-12 |
| GOTERM_BP_FAT | GO:0016071~mRNA metabolic process | 221 | 6.5821E-16 |  | 220 | 6.6433E-16 |
| GOTERM_BP_FAT | GO:0010942~positive regulation of cell death | 221 | 0.03174251 |  | 223 | 0.01153017 |
| GOTERM_BP_FAT | GO:0032386~regulation of intracellular transport | 218 | 0.00022846 |  | 218 | 0.00011883 |
| GOTERM_BP_FAT | GO:0009894~regulation of catabolic process | 216 | 6.5472E-07 |  | 217 | 1.4091E-07 |
| GOTERM_BP_FAT | GO:0000226~microtubule cytoskeleton organization | 216 | 0.00016389 |  | 214 | 0.00022613 |
| GOTERM_BP_FAT | GO:0043068~positive regulation of programmed cell death | 215 | 0.02779211 |  | 217 | 0.00985372 |
| GOTERM_BP_FAT | GO:0043065~positive regulation of apoptotic process | 212 | 0.03695254 |  | 214 | 0.01373364 |
| GOTERM_BP_FAT | GO:1902532~negative regulation of intracellular signal transduction | 208 | 0.00030312 |  | 216 | 1.7644E-06 |
| GOTERM_BP_FAT | GO:0030162~regulation of proteolysis | 208 | 0.00101795 |  | 211 | 0.00013821 |
| GOTERM_BP_FAT | GO:0048285~organelle fission | 201 | 0.03068815 |  | 206 | 0.00383854 |
| GOTERM_BP_FAT | GO:0007346~regulation of mitotic cell cycle | 196 | 4.8041E-05 |  | 200 | 2.0555E-06 |
| GOTERM_BP_FAT | GO:0051347~positive regulation of transferase activity | 196 | 0.04933942 |  | 199 | 0.01401766 |
| GOTERM_BP_FAT | GO:0051169~nuclear transport | 194 | 3.7873E-06 |  | 195 | 9.115E-07 |
| GOTERM_BP_FAT | GO:0051052~regulation of DNA metabolic process | 191 | 2.3063E-07 |  | 190 | 2.2349E-07 |
| GOTERM_BP_FAT | GO:0006913~nucleocytoplasmic transport | 190 | 8.4541E-06 |  | 191 | 2.1421E-06 |
| GOTERM_BP_FAT | GO:2001233~regulation of apoptotic signaling pathway | 189 | 0.00463471 |  | 194 | 0.00030563 |
| GOTERM_BP_FAT | GO:0001701~in utero embryonic development | 188 | 0.01351593 |  | 188 | 0.00880257 |
| GOTERM_BP_FAT | GO:0001525~angiogenesis | 187 | 0.00045126 |  | 182 | 0.00256985 |
| GOTERM_BP_FAT | GO:0030031~cell projection assembly | 183 | 0.01549224 |  | 184 | 0.00714118 |
| GOTERM_BP_FAT | GO:0033157~regulation of intracellular protein transport | 179 | 0.00154894 |  | 181 | 0.0003507 |
| GOTERM_BP_FAT | GO:0016569~covalent chromatin modification | 178 | 0.00012194 |  | 177 | 0.00011776 |
| GOTERM_BP_FAT | GO:0034660~ncRNA metabolic process | 176 | 3.6402E-12 |  | 175 | 4.578E-12 |
| GOTERM_BP_FAT | GO:0071396~cellular response to lipid | 176 | 0.01439154 |  | 180 | 0.00195241 |
| GOTERM_BP_FAT | GO:0071407~cellular response to organic cyclic compound | 175 | 4.3381E-05 |  | 178 | 3.2192E-06 |
| GOTERM_BP_FAT | GO:0051640~organelle localization | 175 | 0.00113095 |  | 172 | 0.00265742 |
| GOTERM_BP_FAT | GO:0051272~positive regulation of cellular component movement | 175 | 0.0161776 |  | 172 | 0.02920853 |
| GOTERM_BP_FAT | GO:0040017~positive regulation of locomotion | 175 | 0.02480057 |  | 173 | 0.03193924 |
| GOTERM_BP_FAT | GO:0006897~endocytosis | 175 | 0.04437951 |  | 177 | 0.0166961 |
| GOTERM_BP_FAT | GO:0045786~negative regulation of cell cycle | 171 | 0.00152761 |  | 171 | 0.00092574 |
| GOTERM_BP_FAT | GO:2000147~positive regulation of cell motility | 171 | 0.01315217 |  | 168 | 0.02467052 |
| GOTERM_BP_FAT | GO:0016236~macroautophagy | 170 | 3.2051E-05 |  | 169 | 3.2389E-05 |
| GOTERM_BP_FAT | GO:0016570~histone modification | 170 | 0.0002366 |  | 169 | 0.00023387 |
| GOTERM_BP_FAT | GO:0051259~protein oligomerization | 170 | 0.04193613 |  | 170 | 0.02971924 |
| GOTERM_BP_FAT | GO:0030335~positive regulation of cell migration | 169 | 0.00632538 |  | 166 | 0.01293394 |
| GOTERM_BP_FAT | GO:1901699~cellular response to nitrogen compound | 169 | 0.03840556 |  | 170 | 0.0196513 |
| GOTERM_BP_FAT | GO:0042326~negative regulation of phosphorylation | 167 | 0.047035 |  | 179 | 0.0002466 |
| GOTERM_BP_FAT | GO:0034504~protein localization to nucleus | 165 | 0.0048793 |  | 165 | 0.00312852 |
| GOTERM_BP_FAT | GO:0044770~cell cycle phase transition | 164 | 4.9884E-07 |  | 168 | 7.1646E-09 |
| GOTERM_BP_FAT | GO:0006397~mRNA processing | 162 | 4.5717E-13 |  | 162 | 1.8006E-13 |
| GOTERM_BP_FAT | GO:0022613~ribonucleoprotein complex biogenesis | 161 | 2.3102E-13 |  | 160 | 3.5359E-13 |
| GOTERM_BP_FAT | GO:0042176~regulation of protein catabolic process | 158 | 6.6419E-07 |  | 163 | 3.5489E-09 |
| GOTERM_BP_FAT | GO:0010498~proteasomal protein catabolic process | 158 | 1.1693E-06 |  | 161 | 4.5721E-08 |
| GOTERM_BP_FAT | GO:0001933~negative regulation of protein phosphorylation | 157 | 0.00648572 |  | 167 | 2.0283E-05 |
| GOTERM_BP_FAT | GO:0010608~posttranscriptional regulation of gene expression | 155 | 4.5194E-05 |  | 156 | 1.2783E-05 |
| GOTERM_BP_FAT | GO:0007265~Ras protein signal transduction | 155 | 0.00103766 |  | 160 | 3.4734E-05 |
| GOTERM_BP_FAT | GO:1903829~positive regulation of cellular protein localization | 155 | 0.0345804 |  | 155 | 0.02467277 |
| GOTERM_BP_FAT | GO:0033044~regulation of chromosome organization | 154 | 3.5231E-05 |  | 155 | 9.7874E-06 |
| GOTERM_BP_FAT | GO:0044772~mitotic cell cycle phase transition | 152 | 4.8888E-07 |  | 156 | 6.0067E-09 |
| GOTERM_BP_FAT | GO:0051098~regulation of binding | 152 | 0.00232362 |  | 151 | 0.00238259 |
| GOTERM_BP_FAT | GO:0097193~intrinsic apoptotic signaling pathway | 151 | 9.9745E-05 |  | 147 | 0.00061175 |
| GOTERM_BP_FAT | GO:0007059~chromosome segregation | 149 | 0.00048266 |  | 150 | 0.00016288 |
| GOTERM_BP_FAT | GO:0051656~establishment of organelle localization | 145 | 0.00192569 |  | 143 | 0.00326605 |
| GOTERM_BP_FAT | GO:0017038~protein import | 144 | 0.00161998 |  | 143 | 0.00171787 |
| GOTERM_BP_FAT | GO:0031329~regulation of cellular catabolic process | 141 | 0.00029378 |  | 142 | 9.4999E-05 |
| GOTERM_BP_FAT | GO:0018205~peptidyl-lysine modification | 140 | 1.5451E-06 |  | 141 | 3.4378E-07 |
| GOTERM_BP_FAT | GO:0043161~proteasome-mediated ubiquitin-dependent protein catabolic process | 139 | 1.9725E-06 |  | 141 | 1.8017E-07 |
| GOTERM_BP_FAT | GO:0007034~vacuolar transport | 138 | 7.4774E-07 |  | 135 | 5.0784E-06 |
| GOTERM_BP_FAT | GO:0034248~regulation of cellular amide metabolic process | 138 | 0.00014752 |  | 141 | 1.08E-05 |
| GOTERM_BP_FAT | GO:0006979~response to oxidative stress | 138 | 0.01703697 |  | 139 | 0.00796298 |
| GOTERM_BP_FAT | GO:1903533~regulation of protein targeting | 138 | 0.03453153 |  | 138 | 0.02516569 |
| GOTERM_BP_FAT | GO:0008380~RNA splicing | 135 | 7.9545E-12 |  | 135 | 3.5935E-12 |
| GOTERM_BP_FAT | GO:0051301~cell division | 134 | 0.00415346 |  | 133 | 0.00448842 |
| GOTERM_BP_FAT | GO:0006417~regulation of translation | 131 | 1.3174E-05 |  | 131 | 7.5569E-06 |
| GOTERM_BP_FAT | GO:0007067~mitotic nuclear division | 131 | 0.00015814 |  | 136 | 2.2454E-06 |
| GOTERM_BP_FAT | GO:0006260~DNA replication | 129 | 1.4234E-07 |  | 129 | 7.5246E-08 |
| GOTERM_BP_FAT | GO:0009101~glycoprotein biosynthetic process | 128 | 0.02272883 |  | 125 | 0.04866281 |
| GOTERM_BP_FAT | GO:0060271~cilium morphogenesis | 127 | 0.00881757 |  | 127 | 0.0061478 |
| GOTERM_BP_FAT | GO:0007249~I-kappaB kinase/NF-kappaB signaling | 124 | 0.0075763 |  | 122 | 0.01294265 |
| GOTERM_BP_FAT | GO:0051170~nuclear import | 122 | 0.0232502 |  | 122 | 0.01699947 |
| GOTERM_BP_FAT | GO:0044744~protein targeting to nucleus | 121 | 0.02049491 |  | 121 | 0.01492546 |
| GOTERM_BP_FAT | GO:0006606~protein import into nucleus | 121 | 0.02049491 |  | 121 | 0.01492546 |
| GOTERM_BP_FAT | GO:1902593~single-organism nuclear import | 121 | 0.02049491 |  | 121 | 0.01492546 |
| GOTERM_BP_FAT | GO:0051056~regulation of small GTPase mediated signal transduction | 118 | 0.04853781 |  | 123 | 0.00449563 |
| GOTERM_BP_FAT | GO:0016197~endosomal transport | 117 | 6.6009E-06 |  | 115 | 2.1186E-05 |
| GOTERM_BP_FAT | GO:0016311~dephosphorylation | 117 | 0.00010781 |  | 114 | 0.00052938 |
| GOTERM_BP_FAT | GO:0043122~regulation of I-kappaB kinase/NF-kappaB signaling | 117 | 0.01576241 |  | 116 | 0.01751652 |
| GOTERM_BP_FAT | GO:0031647~regulation of protein stability | 116 | 0.00013404 |  | 113 | 0.00064688 |
| GOTERM_BP_FAT | GO:0090150~establishment of protein localization to membrane | 116 | 0.01368422 |  | 114 | 0.02312785 |
| GOTERM_BP_FAT | GO:1903320~regulation of protein modification by small protein conjugation or removal | 115 | 1.9662E-05 |  | 115 | 1.1859E-05 |
| GOTERM_BP_FAT | GO:0046700~heterocycle catabolic process | 115 | 0.01181984 |  | 112 | 0.03025813 |
| GOTERM_BP_FAT | GO:0044270~cellular nitrogen compound catabolic process | 115 | 0.01181984 |  | 112 | 0.03025813 |
| GOTERM_BP_FAT | GO:0046777~protein autophosphorylation | 114 | 7.508E-05 |  | 109 | 0.00141223 |
| GOTERM_BP_FAT | GO:0010506~regulation of autophagy | 113 | 0.00097214 |  | 115 | 0.00017196 |
| GOTERM_BP_FAT | GO:0018209~peptidyl-serine modification | 113 | 0.00313414 |  | 109 | 0.01567577 |
| GOTERM_BP_FAT | GO:0045862~positive regulation of proteolysis | 113 | 0.01180945 |  | 114 | 0.00525233 |
| GOTERM_BP_FAT | GO:0098813~nuclear chromosome segregation | 113 | 0.02729608 |  | 114 | 0.0133367 |
| GOTERM_BP_FAT | GO:0034976~response to endoplasmic reticulum stress | 112 | 8.6469E-07 |  | 111 | 1.3493E-06 |
| GOTERM_BP_FAT | GO:0009896~positive regulation of catabolic process | 110 | 0.00522222 |  | 110 | 0.00367905 |
| GOTERM_BP_FAT | GO:0019058~viral life cycle | 109 | 0.00616379 |  | 108 | 0.00726585 |
| GOTERM_BP_FAT | GO:0071826~ribonucleoprotein complex subunit organization | 108 | 1.1731E-10 |  | 107 | 2.7492E-10 |
| GOTERM_BP_FAT | GO:0034470~ncRNA processing | 108 | 3.0696E-09 |  | 108 | 1.6489E-09 |
| GOTERM_BP_FAT | GO:1901987~regulation of cell cycle phase transition | 108 | 0.0002813 |  | 110 | 4.0171E-05 |
| GOTERM_BP_FAT | GO:0034655~nucleobase-containing compound catabolic process | 108 | 0.00071318 |  | 104 | 0.0051242 |
| GOTERM_BP_FAT | GO:0046822~regulation of nucleocytoplasmic transport | 108 | 0.00247194 |  | 108 | 0.00170738 |
| GOTERM_BP_FAT | GO:0007018~microtubule-based movement | 108 | 0.04063065 |  | 107 | 0.04519166 |
| GOTERM_BP_FAT | GO:0000375~RNA splicing, via transesterification reactions | 107 | 2.8848E-08 |  | 107 | 1.6002E-08 |
| GOTERM_BP_FAT | GO:0000377~RNA splicing, via transesterification reactions with bulged adenosine as nucleophile | 107 | 2.8848E-08 |  | 107 | 1.6002E-08 |
| GOTERM_BP_FAT | GO:0000398~mRNA splicing, via spliceosome | 107 | 2.8848E-08 |  | 107 | 1.6002E-08 |
| GOTERM_BP_FAT | GO:0051054~positive regulation of DNA metabolic process | 107 | 1.2677E-05 |  | 107 | 7.7469E-06 |
| GOTERM_BP_FAT | GO:0046578~regulation of Ras protein signal transduction | 107 | 0.02118843 |  | 111 | 0.00212865 |
| GOTERM_BP_FAT | GO:0031396~regulation of protein ubiquitination | 106 | 1.6332E-05 |  | 107 | 4.0157E-06 |
| GOTERM_BP_FAT | GO:0043543~protein acylation | 106 | 0.0002649 |  | 107 | 8.1648E-05 |
| GOTERM_BP_FAT | GO:1900180~regulation of protein localization to nucleus | 106 | 0.04102119 |  | 106 | 0.03146001 |
| GOTERM_BP_FAT | GO:0018105~peptidyl-serine phosphorylation | 105 | 0.00602616 |  | 102 | 0.01856983 |
| GOTERM_BP_FAT | GO:0022618~ribonucleoprotein complex assembly | 103 | 6.2713E-11 |  | 102 | 1.6398E-10 |
| GOTERM_BP_FAT | GO:1901990~regulation of mitotic cell cycle phase transition | 102 | 7.8865E-05 |  | 104 | 8.9319E-06 |
| GOTERM_BP_FAT | GO:0034330~cell junction organization | 102 | 0.00151268 |  | 98 | 0.01003325 |
| GOTERM_BP_FAT | GO:0006310~DNA recombination | 102 | 0.02459095 |  | 102 | 0.01856983 |
| GOTERM_BP_FAT | GO:0048193~Golgi vesicle transport | 101 | 4.6976E-13 |  | 102 | 2.9812E-14 |
| GOTERM_BP_FAT | GO:0006302~double-strand break repair | 101 | 5.6349E-05 |  | 101 | 3.6047E-05 |
| GOTERM_BP_FAT | GO:0007033~vacuole organization | 100 | 5.7828E-07 |  | 99 | 1.0292E-06 |
| GOTERM_BP_FAT | GO:0000075~cell cycle checkpoint | 99 | 2.7475E-05 |  | 99 | 1.7425E-05 |
| GOTERM_BP_FAT | GO:1903008~organelle disassembly | 99 | 0.02843995 |  | 97 | 0.04878588 |
| GOTERM_BP_FAT | GO:1903362~regulation of cellular protein catabolic process | 95 | 2.1227E-05 |  | 97 | 1.8453E-06 |
| GOTERM_BP_FAT | GO:0030522~intracellular receptor signaling pathway | 95 | 0.00039483 |  | 97 | 5.3973E-05 |
| GOTERM_BP_FAT | GO:0032259~methylation | 94 | 0.00665115 |  | 92 | 0.01391223 |
| GOTERM_BP_FAT | GO:0043414~macromolecule methylation | 92 | 0.00939764 |  | 90 | 0.01917689 |
| GOTERM_BP_FAT | GO:0045930~negative regulation of mitotic cell cycle | 91 | 0.00034124 |  | 93 | 4.396E-05 |
| GOTERM_BP_FAT | GO:0071496~cellular response to external stimulus | 91 | 0.03822204 |  | 95 | 0.00404795 |
| GOTERM_BP_FAT | GO:2001020~regulation of response to DNA damage stimulus | 90 | 0.00024915 |  | 91 | 7.3285E-05 |
| GOTERM_BP_FAT | GO:0045216~cell-cell junction organization | 90 | 0.00640804 |  | 86 | 0.03524149 |
| GOTERM_BP_FAT | GO:0007160~cell-matrix adhesion | 90 | 0.02490049 |  | 88 | 0.04577855 |
| GOTERM_BP_FAT | GO:0043393~regulation of protein binding | 90 | 0.03334341 |  | 91 | 0.01635602 |
| GOTERM_BP_FAT | GO:2001252~positive regulation of chromosome organization | 89 | 5.2675E-05 |  | 89 | 3.4784E-05 |
| GOTERM_BP_FAT | GO:0006457~protein folding | 89 | 9.8747E-05 |  | 84 | 0.00290831 |
| GOTERM_BP_FAT | GO:0000819~sister chromatid segregation | 89 | 0.00017911 |  | 91 | 2.0344E-05 |
| GOTERM_BP_FAT | GO:0048545~response to steroid hormone | 89 | 0.00522155 |  | 92 | 0.00052792 |
| GOTERM_BP_FAT | GO:0000209~protein polyubiquitination | 88 | 0.00022776 |  | 88 | 0.00015517 |
| GOTERM_BP_FAT | GO:2001242~regulation of intrinsic apoptotic signaling pathway | 88 | 0.00067195 |  | 86 | 0.00192762 |
| GOTERM_BP_FAT | GO:0051348~negative regulation of transferase activity | 88 | 0.0441867 |  | 94 | 0.00141866 |
| GOTERM_BP_FAT | GO:2001235~positive regulation of apoptotic signaling pathway | 86 | 0.00898458 |  | 87 | 0.0037453 |
| GOTERM_BP_FAT | GO:1904589~regulation of protein import | 86 | 0.03357129 |  | 86 | 0.02631269 |
| GOTERM_BP_FAT | GO:0006473~protein acetylation | 85 | 0.00078471 |  | 87 | 0.00010994 |
| GOTERM_BP_FAT | GO:0042306~regulation of protein import into nucleus | 85 | 0.03881704 |  | 85 | 0.03064056 |
| GOTERM_BP_FAT | GO:1902275~regulation of chromatin organization | 84 | 0.00395484 |  | 84 | 0.00290831 |
| GOTERM_BP_FAT | GO:0034599~cellular response to oxidative stress | 84 | 0.02483432 |  | 84 | 0.01931687 |
| GOTERM_BP_FAT | GO:0010948~negative regulation of cell cycle process | 83 | 0.01508469 |  | 84 | 0.00663709 |
| GOTERM_BP_FAT | GO:1903050~regulation of proteolysis involved in cellular protein catabolic process | 82 | 0.00016715 |  | 84 | 1.7275E-05 |
| GOTERM_BP_FAT | GO:0034329~cell junction assembly | 82 | 0.00381606 |  | 80 | 0.00947418 |
| GOTERM_BP_FAT | GO:0006399~tRNA metabolic process | 81 | 5.8135E-11 |  | 81 | 3.3661E-11 |
| GOTERM_BP_FAT | GO:0040029~regulation of gene expression, epigenetic | 81 | 5.8971E-05 |  | 81 | 3.9937E-05 |
| GOTERM_BP_FAT | GO:0045732~positive regulation of protein catabolic process | 81 | 0.00187975 |  | 84 | 0.00012803 |
| GOTERM_BP_FAT | GO:0016050~vesicle organization | 80 | 0.00564556 |  | 81 | 0.00220203 |
| GOTERM_BP_FAT | GO:0031331~positive regulation of cellular catabolic process | 80 | 0.03375734 |  | 79 | 0.04204011 |
| GOTERM_BP_FAT | GO:0006650~glycerophospholipid metabolic process | 79 | 0.01473096 |  | 82 | 0.00177919 |
| GOTERM_BP_FAT | GO:0071383~cellular response to steroid hormone stimulus | 78 | 0.00044913 |  | 80 | 5.2575E-05 |
| GOTERM_BP_FAT | GO:0070646~protein modification by small protein removal | 78 | 0.00133966 |  | 77 | 0.00201753 |
| GOTERM_BP_FAT | GO:0007163~establishment or maintenance of cell polarity | 78 | 0.01216521 |  | 77 | 0.01620381 |
| GOTERM_BP_FAT | GO:0000070~mitotic sister chromatid segregation | 77 | 8.7013E-05 |  | 79 | 7.4482E-06 |
| GOTERM_BP_FAT | GO:1903828~negative regulation of cellular protein localization | 77 | 0.0066395 |  | 78 | 0.00261513 |
| GOTERM_BP_FAT | GO:0042254~ribosome biogenesis | 76 | 5.6541E-05 |  | 76 | 3.8843E-05 |
| GOTERM_BP_FAT | GO:0044843~cell cycle G1/S phase transition | 76 | 0.00072527 |  | 78 | 9.0491E-05 |
| GOTERM_BP_FAT | GO:0043087~regulation of GTPase activity | 76 | 0.00336401 |  | 74 | 0.00904921 |
| GOTERM_BP_FAT | GO:1902115~regulation of organelle assembly | 76 | 0.00336401 |  | 75 | 0.00488252 |
| GOTERM_BP_FAT | GO:0006469~negative regulation of protein kinase activity | 75 | 0.02874339 |  | 80 | 0.00103377 |
| GOTERM_BP_FAT | GO:0009755~hormone-mediated signaling pathway | 74 | 0.01705785 |  | 77 | 0.00201753 |
| GOTERM_BP_FAT | GO:0009411~response to UV | 73 | 0.00145907 |  | 74 | 0.00047851 |
| GOTERM_BP_FAT | GO:0018394~peptidyl-lysine acetylation | 73 | 0.00145907 |  | 74 | 0.00047851 |
| GOTERM_BP_FAT | GO:0035303~regulation of dephosphorylation | 73 | 0.00946503 |  | 72 | 0.01318935 |
| GOTERM_BP_FAT | GO:0031056~regulation of histone modification | 72 | 0.00756745 |  | 72 | 0.00580898 |
| GOTERM_BP_FAT | GO:0071103~DNA conformation change | 72 | 0.01680269 |  | 72 | 0.01318935 |
| GOTERM_BP_FAT | GO:0070585~protein localization to mitochondrion | 72 | 0.02406457 |  | 71 | 0.03183416 |
| GOTERM_BP_FAT | GO:0006401~RNA catabolic process | 71 | 5.2439E-05 |  | 70 | 0.00010277 |
| GOTERM_BP_FAT | GO:0051100~negative regulation of binding | 71 | 0.00376113 |  | 71 | 0.00284336 |
| GOTERM_BP_FAT | GO:0061136~regulation of proteasomal protein catabolic process | 70 | 0.00097753 |  | 72 | 0.00011905 |
| GOTERM_BP_FAT | GO:0072331~signal transduction by p53 class mediator | 70 | 0.00464765 |  | 68 | 0.01280825 |
| GOTERM_BP_FAT | GO:0009895~negative regulation of catabolic process | 70 | 0.04599995 |  | 73 | 0.00729414 |
| GOTERM_BP_FAT | GO:0034249~negative regulation of cellular amide metabolic process | 69 | 9.2666E-05 |  | 70 | 2.2192E-05 |
| GOTERM_BP_FAT | GO:0072655~establishment of protein localization to mitochondrion | 69 | 0.01976189 |  | 68 | 0.02694551 |
| GOTERM_BP_FAT | GO:0006261~DNA-dependent DNA replication | 68 | 1.0642E-05 |  | 68 | 7.3234E-06 |
| GOTERM_BP_FAT | GO:1903311~regulation of mRNA metabolic process | 68 | 0.00024866 |  | 66 | 0.00108223 |
| GOTERM_BP_FAT | GO:0016579~protein deubiquitination | 68 | 0.00268566 |  | 67 | 0.00420878 |
| GOTERM_BP_FAT | GO:0071824~protein-DNA complex subunit organization | 68 | 0.01620642 |  | 68 | 0.01280825 |
| GOTERM_BP_FAT | GO:0002218~activation of innate immune response | 68 | 0.02356258 |  | 68 | 0.01882789 |
| GOTERM_BP_FAT | GO:0051168~nuclear export | 67 | 7.1493E-07 |  | 67 | 4.7832E-07 |
| GOTERM_BP_FAT | GO:0016482~cytosolic transport | 67 | 1.4569E-05 |  | 64 | 0.00027059 |
| GOTERM_BP_FAT | GO:0000082~G1/S transition of mitotic cell cycle | 67 | 0.00198868 |  | 69 | 0.00026867 |
| GOTERM_BP_FAT | GO:0006475~internal protein amino acid acetylation | 67 | 0.00860827 |  | 69 | 0.00160647 |
| GOTERM_BP_FAT | GO:0031570~DNA integrity checkpoint | 66 | 1.0142E-06 |  | 65 | 2.8442E-06 |
| GOTERM_BP_FAT | GO:0017148~negative regulation of translation | 66 | 1.9902E-05 |  | 66 | 1.3907E-05 |
| GOTERM_BP_FAT | GO:0006275~regulation of DNA replication | 65 | 6.2695E-05 |  | 64 | 0.00013147 |
| GOTERM_BP_FAT | GO:0043401~steroid hormone mediated signaling pathway | 65 | 0.00102606 |  | 67 | 0.0001165 |
| GOTERM_BP_FAT | GO:0010212~response to ionizing radiation | 65 | 0.00183353 |  | 68 | 8.751E-05 |
| GOTERM_BP_FAT | GO:1903322~positive regulation of protein modification by small protein conjugation or removal | 65 | 0.00314588 |  | 66 | 0.00108223 |
| GOTERM_BP_FAT | GO:0018393~internal peptidyl-lysine acetylation | 65 | 0.00828443 |  | 66 | 0.00320783 |
| GOTERM_BP_FAT | GO:0006403~RNA localization | 64 | 3.689E-05 |  | 64 | 2.6173E-05 |
| GOTERM_BP_FAT | GO:0031023~microtubule organizing center organization | 64 | 0.00071204 |  | 64 | 0.00052937 |
| GOTERM_BP_FAT | GO:0071478~cellular response to radiation | 64 | 0.01548457 |  | 66 | 0.00320783 |
| GOTERM_BP_FAT | GO:0016573~histone acetylation | 63 | 0.01238882 |  | 64 | 0.0050073 |
| GOTERM_BP_FAT | GO:0050821~protein stabilization | 63 | 0.01238882 |  | 61 | 0.03181493 |
| GOTERM_BP_FAT | GO:0000077~DNA damage checkpoint | 62 | 3.9647E-07 |  | 61 | 1.313E-06 |
| GOTERM_BP_FAT | GO:0030518~intracellular steroid hormone receptor signaling pathway | 62 | 0.00062749 |  | 63 | 0.00017529 |
| GOTERM_BP_FAT | GO:0051092~positive regulation of NF-kappaB transcription factor activity | 62 | 0.00611274 |  | 59 | 0.03167825 |
| GOTERM_BP_FAT | GO:0046488~phosphatidylinositol metabolic process | 62 | 0.03261498 |  | 63 | 0.0149919 |
| GOTERM_BP_FAT | GO:0002221~pattern recognition receptor signaling pathway | 62 | 0.04591543 |  | 62 | 0.03787798 |
| GOTERM_BP_FAT | GO:0007051~spindle organization | 61 | 3.9003E-05 |  | 61 | 2.7979E-05 |
| GOTERM_BP_FAT | GO:0000910~cytokinesis | 61 | 0.00270879 |  | 60 | 0.00452633 |
| GOTERM_BP_FAT | GO:0006402~mRNA catabolic process | 60 | 5.3229E-05 |  | 59 | 0.00012193 |
| GOTERM_BP_FAT | GO:0032387~negative regulation of intracellular transport | 60 | 0.00194718 |  | 61 | 0.00061306 |
| GOTERM_BP_FAT | GO:0051297~centrosome organization | 60 | 0.00194718 |  | 60 | 0.00149162 |
| GOTERM_BP_FAT | GO:0008630~intrinsic apoptotic signaling pathway in response to DNA damage | 60 | 0.00342584 |  | 61 | 0.00115658 |
| GOTERM_BP_FAT | GO:0051053~negative regulation of DNA metabolic process | 60 | 0.01462352 |  | 60 | 0.0117177 |
| GOTERM_BP_FAT | GO:0031669~cellular response to nutrient levels | 60 | 0.01462352 |  | 62 | 0.00284572 |
| GOTERM_BP_FAT | GO:0006338~chromatin remodeling | 60 | 0.04576203 |  | 60 | 0.03788195 |
| GOTERM_BP_FAT | GO:0016241~regulation of macroautophagy | 59 | 0.00016545 |  | 59 | 0.00012193 |
| GOTERM_BP_FAT | GO:0031398~positive regulation of protein ubiquitination | 59 | 0.00248467 |  | 60 | 0.00080049 |
| GOTERM_BP_FAT | GO:0016458~gene silencing | 58 | 0.0031602 |  | 58 | 0.00245713 |
| GOTERM_BP_FAT | GO:0007093~mitotic cell cycle checkpoint | 56 | 0.00157172 |  | 57 | 0.0004669 |
| GOTERM_BP_FAT | GO:0000723~telomere maintenance | 55 | 0.0002426 |  | 55 | 0.00018214 |
| GOTERM_BP_FAT | GO:0032200~telomere organization | 55 | 0.0002426 |  | 55 | 0.00018214 |
| GOTERM_BP_FAT | GO:0042177~negative regulation of protein catabolic process | 55 | 0.00637072 |  | 56 | 0.00225482 |
| GOTERM_BP_FAT | GO:0071897~DNA biosynthetic process | 55 | 0.02551271 |  | 57 | 0.00538051 |
| GOTERM_BP_FAT | GO:0043484~regulation of RNA splicing | 55 | 0.02551271 |  | 55 | 0.02096394 |
| GOTERM_BP_FAT | GO:0000956~nuclear-transcribed mRNA catabolic process | 54 | 7.1822E-06 |  | 52 | 8.2392E-05 |
| GOTERM_BP_FAT | GO:0010970~establishment of localization by movement along microtubule | 54 | 5.7781E-05 |  | 55 | 1.1051E-05 |
| GOTERM_BP_FAT | GO:0006470~protein dephosphorylation | 54 | 0.00032592 |  | 53 | 0.00070638 |
| GOTERM_BP_FAT | GO:0006997~nucleus organization | 54 | 0.03082098 |  | 54 | 0.02550785 |
| GOTERM_BP_FAT | GO:0007030~Golgi organization | 53 | 1.0228E-05 |  | 53 | 7.4397E-06 |
| GOTERM_BP_FAT | GO:0009451~RNA modification | 53 | 1.0228E-05 |  | 54 | 1.5297E-06 |
| GOTERM_BP_FAT | GO:0001952~regulation of cell-matrix adhesion | 53 | 0.00593135 |  | 52 | 0.01010199 |
| GOTERM_BP_FAT | GO:0032434~regulation of proteasomal ubiquitin-dependent protein catabolic process | 53 | 0.00998578 |  | 54 | 0.00370913 |
| GOTERM_BP_FAT | GO:0010508~positive regulation of autophagy | 52 | 0.00120566 |  | 50 | 0.00560727 |
| GOTERM_BP_FAT | GO:0090317~negative regulation of intracellular protein transport | 52 | 0.00429603 |  | 53 | 0.00140776 |
| GOTERM_BP_FAT | GO:0030218~erythrocyte differentiation | 52 | 0.01243123 |  | 50 | 0.03696829 |
| GOTERM_BP_FAT | GO:1901988~negative regulation of cell cycle phase transition | 52 | 0.01976757 |  | 54 | 0.00370913 |
| GOTERM_BP_FAT | GO:0050684~regulation of mRNA processing | 51 | 0.0015815 |  | 51 | 0.00123783 |
| GOTERM_BP_FAT | GO:0048284~organelle fusion | 51 | 0.00302704 |  | 50 | 0.00560727 |
| GOTERM_BP_FAT | GO:0031503~protein complex localization | 51 | 0.01541498 |  | 51 | 0.01261819 |
| GOTERM_BP_FAT | GO:0044839~cell cycle G2/M phase transition | 50 | 0.00103032 |  | 50 | 0.00080326 |
| GOTERM_BP_FAT | GO:0048524~positive regulation of viral process | 50 | 0.00695233 |  | 48 | 0.02398797 |
| GOTERM_BP_FAT | GO:0036503~ERAD pathway | 49 | 0.0002819 |  | 49 | 0.00021639 |
| GOTERM_BP_FAT | GO:1902806~regulation of cell cycle G1/S phase transition | 49 | 0.00269342 |  | 50 | 0.00080326 |
| GOTERM_BP_FAT | GO:1905269~positive regulation of chromatin organization | 49 | 0.03577495 |  | 49 | 0.03003238 |
| GOTERM_BP_FAT | GO:0042770~signal transduction in response to DNA damage | 48 | 0.00015666 |  | 47 | 0.00040597 |
| GOTERM_BP_FAT | GO:0051225~spindle assembly | 48 | 0.00015666 |  | 48 | 0.00011978 |
| GOTERM_BP_FAT | GO:0016072~rRNA metabolic process | 48 | 0.00349624 |  | 47 | 0.00664505 |
| GOTERM_BP_FAT | GO:1903364~positive regulation of cellular protein catabolic process | 48 | 0.01107916 |  | 49 | 0.00402099 |
| GOTERM_BP_FAT | GO:1901991~negative regulation of mitotic cell cycle phase transition | 48 | 0.01107916 |  | 50 | 0.00163036 |
| GOTERM_BP_FAT | GO:2000045~regulation of G1/S transition of mitotic cell cycle | 47 | 0.00236055 |  | 48 | 0.00067496 |
| GOTERM_BP_FAT | GO:0018022~peptidyl-lysine methylation | 47 | 0.00236055 |  | 47 | 0.00188212 |
| GOTERM_BP_FAT | GO:0006282~regulation of DNA repair | 47 | 0.0045215 |  | 48 | 0.00142199 |
| GOTERM_BP_FAT | GO:0031929~TOR signaling | 47 | 0.0045215 |  | 47 | 0.00364595 |
| GOTERM_BP_FAT | GO:0032091~negative regulation of protein binding | 47 | 0.01390146 |  | 47 | 0.01146115 |
| GOTERM_BP_FAT | GO:0000725~recombinational repair | 46 | 0.00582501 |  | 47 | 0.00188212 |
| GOTERM_BP_FAT | GO:0034446~substrate adhesion-dependent cell spreading | 46 | 0.02777333 |  | 45 | 0.04382604 |
| GOTERM_BP_FAT | GO:0007041~lysosomal transport | 45 | 5.5551E-05 |  | 46 | 8.7631E-06 |
| GOTERM_BP_FAT | GO:0034332~adherens junction organization | 45 | 0.00402949 |  | 43 | 0.01726663 |
| GOTERM_BP_FAT | GO:0035966~response to topologically incorrect protein | 45 | 0.00402949 |  | 43 | 0.01726663 |
| GOTERM_BP_FAT | GO:0000724~double-strand break repair via homologous recombination | 45 | 0.00747461 |  | 46 | 0.00248208 |
| GOTERM_BP_FAT | GO:0007569~cell aging | 45 | 0.03404332 |  | 45 | 0.02878545 |
| GOTERM_BP_FAT | GO:0008033~tRNA processing | 44 | 1.4102E-06 |  | 45 | 1.1126E-07 |
| GOTERM_BP_FAT | GO:0042787~protein ubiquitination involved in ubiquitin-dependent protein catabolic process | 44 | 0.00055899 |  | 44 | 0.00044051 |
| GOTERM_BP_FAT | GO:1903902~positive regulation of viral life cycle | 44 | 0.00268424 |  | 41 | 0.02708804 |
| GOTERM_BP_FAT | GO:1903321~negative regulation of protein modification by small protein conjugation or removal | 44 | 0.00268424 |  | 43 | 0.00556254 |
| GOTERM_BP_FAT | GO:0051983~regulation of chromosome segregation | 44 | 0.02676222 |  | 45 | 0.01080468 |
| GOTERM_BP_FAT | GO:0031058~positive regulation of histone modification | 44 | 0.04153607 |  | 44 | 0.03537223 |
| GOTERM_BP_FAT | GO:0000086~G2/M transition of mitotic cell cycle | 43 | 0.00076235 |  | 43 | 0.00060532 |
| GOTERM_BP_FAT | GO:0043331~response to dsRNA | 43 | 0.00171135 |  | 43 | 0.00137441 |
| GOTERM_BP_FAT | GO:0034968~histone lysine methylation | 43 | 0.00353273 |  | 43 | 0.00286939 |
| GOTERM_BP_FAT | GO:0051236~establishment of RNA localization | 43 | 0.00353273 |  | 43 | 0.00286939 |
| GOTERM_BP_FAT | GO:0046823~negative regulation of nucleocytoplasmic transport | 43 | 0.00353273 |  | 43 | 0.00286939 |
| GOTERM_BP_FAT | GO:0006364~rRNA processing | 43 | 0.00677242 |  | 42 | 0.01288339 |
| GOTERM_BP_FAT | GO:2000278~regulation of DNA biosynthetic process | 43 | 0.00677242 |  | 44 | 0.00216404 |
| GOTERM_BP_FAT | GO:0015931~nucleobase-containing compound transport | 43 | 0.0329924 |  | 43 | 0.02799872 |
| GOTERM_BP_FAT | GO:0019079~viral genome replication | 43 | 0.0329924 |  | 43 | 0.02799872 |
| GOTERM_BP_FAT | GO:2001022~positive regulation of response to DNA damage stimulus | 42 | 0.00463129 |  | 43 | 0.00137441 |
| GOTERM_BP_FAT | GO:0032204~regulation of telomere maintenance | 42 | 0.00463129 |  | 42 | 0.00378973 |
| GOTERM_BP_FAT | GO:0007098~centrosome cycle | 42 | 0.01540046 |  | 43 | 0.00556254 |
| GOTERM_BP_FAT | GO:0032006~regulation of TOR signaling | 42 | 0.02563493 |  | 41 | 0.04254385 |
| GOTERM_BP_FAT | GO:1903052~positive regulation of proteolysis involved in cellular protein catabolic process | 41 | 0.01942352 |  | 42 | 0.00722009 |
| GOTERM_BP_FAT | GO:0031397~negative regulation of protein ubiquitination | 40 | 0.0040213 |  | 40 | 0.00330273 |
| GOTERM_BP_FAT | GO:0050658~RNA transport | 40 | 0.00786208 |  | 40 | 0.00652936 |
| GOTERM_BP_FAT | GO:0050657~nucleic acid transport | 40 | 0.00786208 |  | 40 | 0.00652936 |
| GOTERM_BP_FAT | GO:0035967~cellular response to topologically incorrect protein | 40 | 0.01429769 |  | 40 | 0.01200512 |
| GOTERM_BP_FAT | GO:0033143~regulation of intracellular steroid hormone receptor signaling pathway | 40 | 0.02438271 |  | 41 | 0.00933104 |
| GOTERM_BP_FAT | GO:0007044~cell-substrate junction assembly | 40 | 0.02438271 |  | 40 | 0.02069585 |
| GOTERM_BP_FAT | GO:0043038~amino acid activation | 39 | 4.4233E-05 |  | 39 | 3.4572E-05 |
| GOTERM_BP_FAT | GO:0006986~response to unfolded protein | 39 | 0.01017757 |  | 37 | 0.04059819 |
| GOTERM_BP_FAT | GO:0006413~translational initiation | 39 | 0.01817654 |  | 39 | 0.0153737 |
| GOTERM_BP_FAT | GO:0043039~tRNA aminoacylation | 38 | 6.4491E-05 |  | 38 | 5.0792E-05 |
| GOTERM_BP_FAT | GO:0030330~DNA damage response, signal transduction by p53 class mediator | 38 | 0.0002144 |  | 37 | 0.00068831 |
| GOTERM_BP_FAT | GO:1905037~autophagosome organization | 38 | 0.00060935 |  | 38 | 0.00049109 |
| GOTERM_BP_FAT | GO:0030705~cytoskeleton-dependent intracellular transport | 38 | 0.00341181 |  | 39 | 0.00090113 |
| GOTERM_BP_FAT | GO:0000018~regulation of DNA recombination | 38 | 0.02299691 |  | 38 | 0.0195925 |
| GOTERM_BP_FAT | GO:0006888~ER to Golgi vesicle-mediated transport | 37 | 2.3715E-05 |  | 38 | 2.3309E-06 |
| GOTERM_BP_FAT | GO:0006513~protein monoubiquitination | 37 | 0.00455113 |  | 35 | 0.02348324 |
| GOTERM_BP_FAT | GO:2001244~positive regulation of intrinsic apoptotic signaling pathway | 37 | 0.00455113 |  | 36 | 0.01002916 |
| GOTERM_BP_FAT | GO:0060324~face development | 37 | 0.00455113 |  | 36 | 0.01002916 |
| GOTERM_BP_FAT | GO:0042147~retrograde transport, endosome to Golgi | 37 | 0.00910841 |  | 35 | 0.0393184 |
| GOTERM_BP_FAT | GO:0006446~regulation of translational initiation | 37 | 0.02895085 |  | 37 | 0.02484392 |
| GOTERM_BP_FAT | GO:0034644~cellular response to UV | 37 | 0.0468127 |  | 38 | 0.0195925 |
| GOTERM_BP_FAT | GO:0045069~regulation of viral genome replication | 37 | 0.0468127 |  | 37 | 0.04059819 |
| GOTERM_BP_FAT | GO:0006418~tRNA aminoacylation for protein translation | 36 | 0.0001361 |  | 36 | 0.00010884 |
| GOTERM_BP_FAT | GO:0000045~autophagosome assembly | 36 | 0.00117503 |  | 36 | 0.0009614 |
| GOTERM_BP_FAT | GO:0044033~multi-organism metabolic process | 35 | 0.0079941 |  | 33 | 0.03779629 |
| GOTERM_BP_FAT | GO:1904590~negative regulation of protein import | 35 | 0.02726186 |  | 35 | 0.02348324 |
| GOTERM_BP_FAT | GO:0034502~protein localization to chromosome | 35 | 0.02726186 |  | 35 | 0.02348324 |
| GOTERM_BP_FAT | GO:0042308~negative regulation of protein import into nucleus | 35 | 0.02726186 |  | 35 | 0.02348324 |
| GOTERM_BP_FAT | GO:0034620~cellular response to unfolded protein | 35 | 0.02726186 |  | 35 | 0.02348324 |
| GOTERM_BP_FAT | GO:0031047~gene silencing by RNA | 35 | 0.04516588 |  | 35 | 0.0393184 |
| GOTERM_BP_FAT | GO:2000573~positive regulation of DNA biosynthetic process | 34 | 0.00086046 |  | 34 | 0.00070668 |
| GOTERM_BP_FAT | GO:0031123~RNA 3'-end processing | 34 | 0.00086046 |  | 34 | 0.00070668 |
| GOTERM_BP_FAT | GO:0030433~ER-associated ubiquitin-dependent protein catabolic process | 34 | 0.01052249 |  | 34 | 0.00893555 |
| GOTERM_BP_FAT | GO:0098732~macromolecule deacylation | 34 | 0.01979324 |  | 34 | 0.01699123 |
| GOTERM_BP_FAT | GO:0032465~regulation of cytokinesis | 34 | 0.01979324 |  | 35 | 0.00673855 |
| GOTERM_BP_FAT | GO:0035601~protein deacylation | 34 | 0.01979324 |  | 34 | 0.01699123 |
| GOTERM_BP_FAT | GO:0030968~endoplasmic reticulum unfolded protein response | 34 | 0.03443354 |  | 34 | 0.02987551 |
| GOTERM_BP_FAT | GO:0071230~cellular response to amino acid stimulus | 33 | 0.01378358 |  | 32 | 0.02818746 |
| GOTERM_BP_FAT | GO:0090329~regulation of DNA-dependent DNA replication | 32 | 0.00058695 |  | 32 | 0.00048388 |
| GOTERM_BP_FAT | GO:0032206~positive regulation of telomere maintenance | 32 | 0.00168966 |  | 32 | 0.00140878 |
| GOTERM_BP_FAT | GO:0016441~posttranscriptional gene silencing | 32 | 0.00417847 |  | 32 | 0.00352297 |
| GOTERM_BP_FAT | GO:0035194~posttranscriptional gene silencing by RNA | 32 | 0.00417847 |  | 32 | 0.00352297 |
| GOTERM_BP_FAT | GO:0071479~cellular response to ionizing radiation | 32 | 0.00417847 |  | 33 | 0.00099959 |
| GOTERM_BP_FAT | GO:0006289~nucleotide-excision repair | 32 | 0.01796397 |  | 32 | 0.01548039 |
| GOTERM_BP_FAT | GO:0006476~protein deacetylation | 32 | 0.03236308 |  | 32 | 0.02818746 |
| GOTERM_BP_FAT | GO:0010833~telomere maintenance via telomere lengthening | 31 | 0.00235448 |  | 31 | 0.00197792 |
| GOTERM_BP_FAT | GO:0035195~gene silencing by miRNA | 31 | 0.00567859 |  | 31 | 0.0048236 |
| GOTERM_BP_FAT | GO:0072384~organelle transport along microtubule | 31 | 0.01210767 |  | 32 | 0.00352297 |
| GOTERM_BP_FAT | GO:0033619~membrane protein proteolysis | 31 | 0.0410354 |  | 32 | 0.01548039 |
| GOTERM_BP_FAT | GO:0035304~regulation of protein dephosphorylation | 31 | 0.0410354 |  | 31 | 0.03599885 |
| GOTERM_BP_FAT | GO:0006611~protein export from nucleus | 30 | 0.00036227 |  | 29 | 0.00143327 |
| GOTERM_BP_FAT | GO:0044774~mitotic DNA integrity checkpoint | 30 | 0.00119655 |  | 30 | 0.00100151 |
| GOTERM_BP_FAT | GO:0006405~RNA export from nucleus | 30 | 0.00768187 |  | 30 | 0.00657397 |
| GOTERM_BP_FAT | GO:0071166~ribonucleoprotein complex localization | 30 | 0.01598118 |  | 31 | 0.0048236 |
| GOTERM_BP_FAT | GO:0016239~positive regulation of macroautophagy | 30 | 0.03002262 |  | 30 | 0.0262512 |
| GOTERM_BP_FAT | GO:1903363~negative regulation of cellular protein catabolic process | 30 | 0.03002262 |  | 31 | 0.01039774 |
| GOTERM_BP_FAT | GO:0007004~telomere maintenance via telomerase | 29 | 0.00169951 |  | 29 | 0.00143327 |
| GOTERM_BP_FAT | GO:0006278~RNA-dependent DNA biosynthetic process | 29 | 0.00169951 |  | 29 | 0.00143327 |
| GOTERM_BP_FAT | GO:1904356~regulation of telomere maintenance via telomere lengthening | 29 | 0.01034164 |  | 29 | 0.008916 |
| GOTERM_BP_FAT | GO:2001021~negative regulation of response to DNA damage stimulus | 29 | 0.03847908 |  | 29 | 0.03388917 |
| GOTERM_BP_FAT | GO:0032210~regulation of telomere maintenance via telomerase | 28 | 0.00240481 |  | 28 | 0.00204343 |
| GOTERM_BP_FAT | GO:0061640~cytoskeleton-dependent cytokinesis | 28 | 0.00240481 |  | 27 | 0.00736451 |
| GOTERM_BP_FAT | GO:0071359~cellular response to dsRNA | 28 | 0.01385134 |  | 28 | 0.01203042 |
| GOTERM_BP_FAT | GO:0043044~ATP-dependent chromatin remodeling | 28 | 0.02738874 |  | 28 | 0.02404317 |
| GOTERM_BP_FAT | GO:0044088~regulation of vacuole organization | 28 | 0.02738874 |  | 28 | 0.02404317 |
| GOTERM_BP_FAT | GO:0033627~cell adhesion mediated by integrin | 28 | 0.02738874 |  | 28 | 0.02404317 |
| GOTERM_BP_FAT | GO:0043967~histone H4 acetylation | 28 | 0.04901585 |  | 28 | 0.04347999 |
| GOTERM_BP_FAT | GO:0001954~positive regulation of cell-matrix adhesion | 27 | 0.00850818 |  | 27 | 0.00736451 |
| GOTERM_BP_FAT | GO:0051298~centrosome duplication | 27 | 0.00850818 |  | 27 | 0.00736451 |
| GOTERM_BP_FAT | GO:0070972~protein localization to endoplasmic reticulum | 27 | 0.00850818 |  | 27 | 0.00736451 |
| GOTERM_BP_FAT | GO:2000779~regulation of double-strand break repair | 27 | 0.01845222 |  | 27 | 0.01614482 |
| GOTERM_BP_FAT | GO:0044773~mitotic DNA damage checkpoint | 26 | 0.00475635 |  | 26 | 0.00410282 |
| GOTERM_BP_FAT | GO:0046580~negative regulation of Ras protein signal transduction | 26 | 0.00475635 |  | 26 | 0.00410282 |
| GOTERM_BP_FAT | GO:0006024~glycosaminoglycan biosynthetic process | 26 | 0.00475635 |  | 25 | 0.01380078 |
| GOTERM_BP_FAT | GO:0008333~endosome to lysosome transport | 26 | 0.01159201 |  | 26 | 0.01010858 |
| GOTERM_BP_FAT | GO:0051058~negative regulation of small GTPase mediated signal transduction | 26 | 0.01159201 |  | 27 | 0.00290167 |
| GOTERM_BP_FAT | GO:0035058~nonmotile primary cilium assembly | 26 | 0.02444131 |  | 26 | 0.02154221 |
| GOTERM_BP_FAT | GO:0000245~spliceosomal complex assembly | 25 | 0.00064332 |  | 25 | 0.00054686 |
| GOTERM_BP_FAT | GO:0031124~mRNA 3'-end processing | 25 | 0.00233951 |  | 25 | 0.00201102 |
| GOTERM_BP_FAT | GO:0008156~negative regulation of DNA replication | 25 | 0.00233951 |  | 24 | 0.00808903 |
| GOTERM_BP_FAT | GO:0032784~regulation of DNA-templated transcription, elongation | 25 | 0.00233951 |  | 25 | 0.00201102 |
| GOTERM_BP_FAT | GO:1903312~negative regulation of mRNA metabolic process | 25 | 0.00664481 |  | 25 | 0.00577488 |
| GOTERM_BP_FAT | GO:0051653~spindle localization | 25 | 0.00664481 |  | 24 | 0.01873414 |
| GOTERM_BP_FAT | GO:0060968~regulation of gene silencing | 25 | 0.01570941 |  | 25 | 0.01380078 |
| GOTERM_BP_FAT | GO:0045739~positive regulation of DNA repair | 25 | 0.01570941 |  | 26 | 0.00410282 |
| GOTERM_BP_FAT | GO:0006284~base-excision repair | 25 | 0.01570941 |  | 25 | 0.01380078 |
| GOTERM_BP_FAT | GO:1902807~negative regulation of cell cycle G1/S phase transition | 25 | 0.032179 |  | 26 | 0.01010858 |
| GOTERM_BP_FAT | GO:2000134~negative regulation of G1/S transition of mitotic cell cycle | 25 | 0.032179 |  | 26 | 0.01010858 |
| GOTERM_BP_FAT | GO:0006892~post-Golgi vesicle-mediated transport | 24 | 0.00095696 |  | 25 | 0.00010333 |
| GOTERM_BP_FAT | GO:0065002~intracellular protein transmembrane transport | 24 | 0.00336156 |  | 23 | 0.01127189 |
| GOTERM_BP_FAT | GO:0045070~positive regulation of viral genome replication | 24 | 0.00336156 |  | 24 | 0.00291147 |
| GOTERM_BP_FAT | GO:0071806~protein transmembrane transport | 24 | 0.00336156 |  | 23 | 0.01127189 |
| GOTERM_BP_FAT | GO:0007032~endosome organization | 24 | 0.00336156 |  | 23 | 0.01127189 |
| GOTERM_BP_FAT | GO:0032007~negative regulation of TOR signaling | 24 | 0.00923834 |  | 23 | 0.02527645 |
| GOTERM_BP_FAT | GO:0030521~androgen receptor signaling pathway | 24 | 0.02116855 |  | 24 | 0.01873414 |
| GOTERM_BP_FAT | GO:2000785~regulation of autophagosome assembly | 24 | 0.02116855 |  | 24 | 0.01873414 |
| GOTERM_BP_FAT | GO:1903573~negative regulation of response to endoplasmic reticulum stress | 24 | 0.02116855 |  | 24 | 0.01873414 |
| GOTERM_BP_FAT | GO:0060976~coronary vasculature development | 24 | 0.02116855 |  | 24 | 0.01873414 |
| GOTERM_BP_FAT | GO:0007029~endoplasmic reticulum organization | 24 | 0.02116855 |  | 23 | 0.04926496 |
| GOTERM_BP_FAT | GO:0022406~membrane docking | 24 | 0.02116855 |  | 23 | 0.04926496 |
| GOTERM_BP_FAT | GO:0000281~mitotic cytokinesis | 23 | 0.00141936 |  | 22 | 0.00602526 |
| GOTERM_BP_FAT | GO:0099518~vesicle cytoskeletal trafficking | 23 | 0.00481024 |  | 23 | 0.00419769 |
| GOTERM_BP_FAT | GO:0006998~nuclear envelope organization | 23 | 0.00481024 |  | 23 | 0.00419769 |
| GOTERM_BP_FAT | GO:0051291~protein heterooligomerization | 23 | 0.02835239 |  | 24 | 0.00808903 |
| GOTERM_BP_FAT | GO:0032212~positive regulation of telomere maintenance via telomerase | 22 | 0.00209854 |  | 22 | 0.00182509 |
| GOTERM_BP_FAT | GO:0043631~RNA polyadenylation | 22 | 0.00209854 |  | 22 | 0.00182509 |
| GOTERM_BP_FAT | GO:1904358~positive regulation of telomere maintenance via telomere lengthening | 22 | 0.00209854 |  | 22 | 0.00182509 |
| GOTERM_BP_FAT | GO:0018023~peptidyl-lysine trimethylation | 22 | 0.0068528 |  | 22 | 0.00602526 |
| GOTERM_BP_FAT | GO:0006354~DNA-templated transcription, elongation | 22 | 0.03772916 |  | 22 | 0.03388211 |
| GOTERM_BP_FAT | GO:0044743~intracellular protein transmembrane import | 21 | 0.00309214 |  | 20 | 0.01223174 |
| GOTERM_BP_FAT | GO:0044380~protein localization to cytoskeleton | 21 | 0.00971619 |  | 21 | 0.00860713 |
| GOTERM_BP_FAT | GO:0006400~tRNA modification | 21 | 0.00971619 |  | 22 | 0.00182509 |
| GOTERM_BP_FAT | GO:0051972~regulation of telomerase activity | 21 | 0.00971619 |  | 21 | 0.00860713 |
| GOTERM_BP_FAT | GO:0090311~regulation of protein deacetylation | 21 | 0.02403314 |  | 21 | 0.02151646 |
| GOTERM_BP_FAT | GO:0000387~spliceosomal snRNP assembly | 21 | 0.02403314 |  | 21 | 0.02151646 |
| GOTERM_BP_FAT | GO:0043330~response to exogenous dsRNA | 21 | 0.0498606 |  | 21 | 0.04510225 |
| GOTERM_BP_FAT | GO:0072698~protein localization to microtubule cytoskeleton | 20 | 0.00105613 |  | 20 | 0.00092239 |
| GOTERM_BP_FAT | GO:0045005~DNA-dependent DNA replication maintenance of fidelity | 20 | 0.00453937 |  | 20 | 0.00400816 |
| GOTERM_BP_FAT | GO:0042073~intraciliary transport | 20 | 0.00453937 |  | 20 | 0.00400816 |
| GOTERM_BP_FAT | GO:0007052~mitotic spindle organization | 20 | 0.00453937 |  | 19 | 0.01728524 |
| GOTERM_BP_FAT | GO:0000184~nuclear-transcribed mRNA catabolic process, nonsense-mediated decay | 20 | 0.00453937 |  | 20 | 0.00400816 |
| GOTERM_BP_FAT | GO:0006378~mRNA polyadenylation | 20 | 0.00453937 |  | 20 | 0.00400816 |
| GOTERM_BP_FAT | GO:0047496~vesicle transport along microtubule | 20 | 0.0137051 |  | 20 | 0.01223174 |
| GOTERM_BP_FAT | GO:0051568~histone H3-K4 methylation | 20 | 0.0326524 |  | 20 | 0.02944865 |
| GOTERM_BP_FAT | GO:1903513~endoplasmic reticulum to cytosol transport | 20 | 0.0326524 |  | 20 | 0.02944865 |
| GOTERM_BP_FAT | GO:0030970~retrograde protein transport, ER to cytosol | 20 | 0.0326524 |  | 20 | 0.02944865 |
| GOTERM_BP_FAT | GO:0050686~negative regulation of mRNA processing | 20 | 0.0326524 |  | 20 | 0.02944865 |
| GOTERM_BP_FAT | GO:0072665~protein localization to vacuole | 20 | 0.0326524 |  | 21 | 0.00860713 |
| GOTERM_BP_FAT | GO:1904292~regulation of ERAD pathway | 19 | 0.00663713 |  | 19 | 0.00590489 |
| GOTERM_BP_FAT | GO:0016233~telomere capping | 19 | 0.04405751 |  | 19 | 0.04002601 |
| GOTERM_BP_FAT | GO:0016073~snRNA metabolic process | 19 | 0.04405751 |  | 19 | 0.04002601 |
| GOTERM_BP_FAT | GO:0031297~replication fork processing | 18 | 0.00246133 |  | 18 | 0.00218276 |
| GOTERM_BP_FAT | GO:0045022~early endosome to late endosome transport | 18 | 0.00966169 |  | 17 | 0.03387129 |
| GOTERM_BP_FAT | GO:0042149~cellular response to glucose starvation | 18 | 0.00966169 |  | 17 | 0.03387129 |
| GOTERM_BP_FAT | GO:0007064~mitotic sister chromatid cohesion | 18 | 0.02680085 |  | 18 | 0.02427755 |
| GOTERM_BP_FAT | GO:0070198~protein localization to chromosome, telomeric region | 18 | 0.02680085 |  | 18 | 0.02427755 |
| GOTERM_BP_FAT | GO:0044786~cell cycle DNA replication | 18 | 0.02680085 |  | 18 | 0.02427755 |
| GOTERM_BP_FAT | GO:0007099~centriole replication | 18 | 0.02680085 |  | 18 | 0.02427755 |
| GOTERM_BP_FAT | GO:0051973~positive regulation of telomerase activity | 18 | 0.02680085 |  | 18 | 0.02427755 |
| GOTERM_BP_FAT | GO:0061028~establishment of endothelial barrier | 17 | 0.03711703 |  | 17 | 0.03387129 |
| GOTERM_BP_FAT | GO:1902235~regulation of endoplasmic reticulum stress-induced intrinsic apoptotic signaling pathway | 17 | 0.03711703 |  | 17 | 0.03387129 |
| GOTERM_BP_FAT | GO:0051642~centrosome localization | 16 | 0.00567553 |  | 15 | 0.02648777 |
| GOTERM_BP_FAT | GO:0001522~pseudouridine synthesis | 16 | 0.02016915 |  | 16 | 0.01835304 |
| GOTERM_BP_FAT | GO:0030511~positive regulation of transforming growth factor beta receptor signaling pathway | 15 | 0.00857765 |  | 14 | 0.03797631 |
| GOTERM_BP_FAT | GO:1903846~positive regulation of cellular response to transforming growth factor beta stimulus | 15 | 0.00857765 |  | 14 | 0.03797631 |
| GOTERM_BP_FAT | GO:0006376~mRNA splice site selection | 15 | 0.02889251 |  | 15 | 0.02648777 |
| GOTERM_BP_FAT | GO:0034508~centromere complex assembly | 15 | 0.02889251 |  | 15 | 0.02648777 |
| GOTERM_BP_FAT | GO:0032786~positive regulation of DNA-templated transcription, elongation | 15 | 0.02889251 |  | 15 | 0.02648777 |
| GOTERM_BP_FAT | GO:0032878~regulation of establishment or maintenance of cell polarity | 15 | 0.02889251 |  | 15 | 0.02648777 |
| GOTERM_BP_FAT | GO:1902750~negative regulation of cell cycle G2/M phase transition | 15 | 0.02889251 |  | 15 | 0.02648777 |
| GOTERM_BP_FAT | GO:0002181~cytoplasmic translation | 15 | 0.02889251 |  | 15 | 0.02648777 |
| GOTERM_BP_FAT | GO:0006891~intra-Golgi vesicle-mediated transport | 15 | 0.02889251 |  | 16 | 0.0051105 |
| GOTERM_BP_FAT | GO:0035024~negative regulation of Rho protein signal transduction | 14 | 0.01291632 |  | 14 | 0.01180848 |
| GOTERM_BP_FAT | GO:0006896~Golgi to vacuole transport | 14 | 0.01291632 |  | 14 | 0.01180848 |
| GOTERM_BP_FAT | GO:0006890~retrograde vesicle-mediated transport, Golgi to ER | 14 | 0.01291632 |  | 14 | 0.01180848 |
| GOTERM_BP_FAT | GO:0071108~protein K48-linked deubiquitination | 14 | 0.04111765 |  | 14 | 0.03797631 |
| GOTERM_BP_FAT | GO:0070536~protein K63-linked deubiquitination | 14 | 0.04111765 |  | 14 | 0.03797631 |
| GOTERM_BP_FAT | GO:0051984~positive regulation of chromosome segregation | 14 | 0.04111765 |  | 14 | 0.03797631 |
| GOTERM_BP_FAT | GO:0010972~negative regulation of G2/M transition of mitotic cell cycle | 14 | 0.04111765 |  | 14 | 0.03797631 |
| GOTERM_BP_FAT | GO:0090670~RNA localization to Cajal body | 13 | 0.01936915 |  | 13 | 0.0178424 |
| GOTERM_BP_FAT | GO:2000104~negative regulation of DNA-dependent DNA replication | 13 | 0.01936915 |  | 13 | 0.0178424 |
| GOTERM_BP_FAT | GO:0090671~telomerase RNA localization to Cajal body | 13 | 0.01936915 |  | 13 | 0.0178424 |
| GOTERM_BP_FAT | GO:0090672~telomerase RNA localization | 13 | 0.01936915 |  | 13 | 0.0178424 |
| GOTERM_BP_FAT | GO:1904874~positive regulation of telomerase RNA localization to Cajal body | 12 | 0.02890901 |  | 12 | 0.02683202 |
| GOTERM_BP_FAT | GO:0071539~protein localization to centrosome | 12 | 0.02890901 |  | 12 | 0.02683202 |
| GOTERM_BP_FAT | GO:0031440~regulation of mRNA 3'-end processing | 12 | 0.02890901 |  | 12 | 0.02683202 |
| GOTERM_BP_FAT | GO:1904872~regulation of telomerase RNA localization to Cajal body | 12 | 0.02890901 |  | 12 | 0.02683202 |
| GOTERM_BP_FAT | GO:0070734~histone H3-K27 methylation | 11 | 0.04291397 |  | 11 | 0.04013141 |
| GOTERM_BP_FAT | GO:0045579~positive regulation of B cell differentiation | 11 | 0.04291397 |  | 11 | 0.04013141 |
| GOTERM_BP_FAT | GO:0060831~smoothened signaling pathway involved in dorsal/ventral neural tube patterning | 11 | 0.04291397 |  | 11 | 0.04013141 |
| GOTERM_BP_FAT | GO:0080182~histone H3-K4 trimethylation | 11 | 0.04291397 |  | 11 | 0.04013141 |
| GOTERM_BP_FAT | GO:0044087~regulation of cellular component biogenesis | 337 | 0.01591789 |  |  |  |
| GOTERM_BP_FAT | GO:0044089~positive regulation of cellular component biogenesis | 193 | 0.03246915 |  |  |  |
| GOTERM_BP_FAT | GO:0022411~cellular component disassembly | 180 | 0.01409893 |  |  |  |
| GOTERM_BP_FAT | GO:0051090~regulation of sequence-specific DNA binding transcription factor activity | 162 | 0.02982961 |  |  |  |
| GOTERM_BP_FAT | GO:0009100~glycoprotein metabolic process | 148 | 0.04899544 |  |  |  |
| GOTERM_BP_FAT | GO:0031589~cell-substrate adhesion | 146 | 0.0264244 |  |  |  |
| GOTERM_BP_FAT | GO:0060249~anatomical structure homeostasis | 145 | 0.04529227 |  |  |  |
| GOTERM_BP_FAT | GO:0019439~aromatic compound catabolic process | 115 | 0.03479176 |  |  |  |
| GOTERM_BP_FAT | GO:1901342~regulation of vasculature development | 108 | 0.0316229 |  |  |  |
| GOTERM_BP_FAT | GO:0051091~positive regulation of sequence-specific DNA binding transcription factor activity | 104 | 0.04140648 |  |  |  |
| GOTERM_BP_FAT | GO:0070085~glycosylation | 102 | 0.03229496 |  |  |  |
| GOTERM_BP_FAT | GO:0043413~macromolecule glycosylation | 99 | 0.02843995 |  |  |  |
| GOTERM_BP_FAT | GO:0006486~protein glycosylation | 99 | 0.02843995 |  |  |  |
| GOTERM_BP_FAT | GO:0071559~response to transforming growth factor beta | 92 | 0.02488578 |  |  |  |
| GOTERM_BP_FAT | GO:0071560~cellular response to transforming growth factor beta stimulus | 91 | 0.02139051 |  |  |  |
| GOTERM_BP_FAT | GO:0010810~regulation of cell-substrate adhesion | 86 | 0.03357129 |  |  |  |
| GOTERM_BP_FAT | GO:0051495~positive regulation of cytoskeleton organization | 81 | 0.0289601 |  |  |  |
| GOTERM_BP_FAT | GO:0043902~positive regulation of multi-organism process | 75 | 0.02051219 |  |  |  |
| GOTERM_BP_FAT | GO:0007179~transforming growth factor beta receptor signaling pathway | 70 | 0.04599995 |  |  |  |
| GOTERM_BP_FAT | GO:1904018~positive regulation of vasculature development | 65 | 0.02771396 |  |  |  |
| GOTERM_BP_FAT | GO:0006479~protein methylation | 63 | 0.0389448 |  |  |  |
| GOTERM_BP_FAT | GO:0008213~protein alkylation | 63 | 0.0389448 |  |  |  |
| GOTERM_BP_FAT | GO:0045766~positive regulation of angiogenesis | 59 | 0.02656403 |  |  |  |
| GOTERM_BP_FAT | GO:0034101~erythrocyte homeostasis | 56 | 0.04525548 |  |  |  |
| GOTERM_BP_FAT | GO:0008360~regulation of cell shape | 55 | 0.01670781 |  |  |  |
| GOTERM_BP_FAT | GO:0008654~phospholipid biosynthetic process | 48 | 0.04322318 |  |  |  |
| GOTERM_BP_FAT | GO:0030203~glycosaminoglycan metabolic process | 45 | 0.03404332 |  |  |  |
| GOTERM_BP_FAT | GO:0051492~regulation of stress fiber assembly | 38 | 0.02299691 |  |  |  |
| GOTERM_BP_FAT | GO:0043149~stress fiber assembly | 37 | 0.02895085 |  |  |  |
| GOTERM_BP_FAT | GO:0030038~contractile actin filament bundle assembly | 37 | 0.02895085 |  |  |  |
| GOTERM_BP_FAT | GO:0043200~response to amino acid | 35 | 0.04516588 |  |  |  |
| GOTERM_BP_FAT | GO:0061515~myeloid cell development | 33 | 0.04325159 |  |  |  |
| GOTERM_BP_FAT | GO:0019080~viral gene expression | 29 | 0.03847908 |  |  |  |
| GOTERM_BP_FAT | GO:0090174~organelle membrane fusion | 26 | 0.04583094 |  |  |  |
| GOTERM_BP_FAT | GO:0000288~nuclear-transcribed mRNA catabolic process, deadenylation-dependent decay | 24 | 0.02116855 |  |  |  |
| GOTERM_BP_FAT | GO:0019076~viral release from host cell | 23 | 0.01277797 |  |  |  |
| GOTERM_BP_FAT | GO:0007031~peroxisome organization | 22 | 0.03772916 |  |  |  |
| GOTERM_BP_FAT | GO:0043616~keratinocyte proliferation | 21 | 0.02403314 |  |  |  |
| GOTERM_BP_FAT | GO:0031572~G2 DNA damage checkpoint | 21 | 0.02403314 |  |  |  |
| GOTERM_BP_FAT | GO:0098751~bone cell development | 21 | 0.0498606 |  |  |  |
| GOTERM_BP_FAT | GO:0031648~protein destabilization | 21 | 0.0498606 |  |  |  |
| GOTERM_BP_FAT | GO:0098781~ncRNA transcription | 20 | 0.0326524 |  |  |  |
| GOTERM_BP_FAT | GO:0051293~establishment of spindle localization | 20 | 0.0326524 |  |  |  |
| GOTERM_BP_FAT | GO:0098927~vesicle-mediated transport between endosomal compartments | 19 | 0.01922372 |  |  |  |
| GOTERM_BP_FAT | GO:0006301~postreplication repair | 14 | 0.04111765 |  |  |  |
| GOTERM_BP_FAT | GO:0009303~rRNA transcription | 14 | 0.04111765 |  |  |  |
| GOTERM_BP_FAT | GO:0000289~nuclear-transcribed mRNA poly(A) tail shortening | 13 | 0.01936915 |  |  |  |
| GOTERM_BP_FAT | GO:0035561~regulation of chromatin binding | 12 | 0.02890901 |  |  |  |
| GOTERM_BP_FAT | GO:0023051~regulation of signaling |  |  |  | 1145 | 0.03843861 |
| GOTERM_BP_FAT | GO:0010646~regulation of cell communication |  |  |  | 1134 | 0.04068328 |
| GOTERM_BP_FAT | GO:0048584~positive regulation of response to stimulus |  |  |  | 748 | 0.02213339 |
| GOTERM_BP_FAT | GO:0043067~regulation of programmed cell death |  |  |  | 545 | 0.02657635 |
| GOTERM_BP_FAT | GO:0042981~regulation of apoptotic process |  |  |  | 535 | 0.03979827 |
| GOTERM_BP_FAT | GO:0023057~negative regulation of signaling |  |  |  | 471 | 0.02238132 |
| GOTERM_BP_FAT | GO:0010648~negative regulation of cell communication |  |  |  | 469 | 0.02277823 |
| GOTERM_BP_FAT | GO:0043085~positive regulation of catalytic activity |  |  |  | 326 | 0.03547113 |
| GOTERM_BP_FAT | GO:0040012~regulation of locomotion |  |  |  | 320 | 0.04280281 |
| GOTERM_BP_FAT | GO:0044092~negative regulation of molecular function |  |  |  | 314 | 0.00704874 |
| GOTERM_BP_FAT | GO:0033993~response to lipid |  |  |  | 237 | 0.0358989 |
| GOTERM_BP_FAT | GO:0045936~negative regulation of phosphate metabolic process |  |  |  | 223 | 0.00110888 |
| GOTERM_BP_FAT | GO:0010563~negative regulation of phosphorus metabolic process |  |  |  | 223 | 0.00110888 |
| GOTERM_BP_FAT | GO:0009725~response to hormone |  |  |  | 209 | 0.01292825 |
| GOTERM_BP_FAT | GO:0043086~negative regulation of catalytic activity |  |  |  | 193 | 0.02208108 |
| GOTERM_BP_FAT | GO:0000280~nuclear division |  |  |  | 189 | 0.01224488 |
| GOTERM_BP_FAT | GO:0032870~cellular response to hormone stimulus |  |  |  | 175 | 0.01081486 |
| GOTERM_BP_FAT | GO:0071900~regulation of protein serine/threonine kinase activity |  |  |  | 162 | 0.01045507 |
| GOTERM_BP_FAT | GO:0043903~regulation of symbiosis, encompassing mutualism through parasitism |  |  |  | 156 | 0.04094208 |
| GOTERM_BP_FAT | GO:0050792~regulation of viral process |  |  |  | 146 | 0.01880557 |
| GOTERM_BP_FAT | GO:0097191~extrinsic apoptotic signaling pathway |  |  |  | 120 | 0.00187591 |
| GOTERM_BP_FAT | GO:0043405~regulation of MAP kinase activity |  |  |  | 109 | 0.04449061 |
| GOTERM_BP_FAT | GO:0071214~cellular response to abiotic stimulus |  |  |  | 106 | 0.0136513 |
| GOTERM_BP_FAT | GO:2001236~regulation of extrinsic apoptotic signaling pathway |  |  |  | 88 | 0.00463535 |
| GOTERM_BP_FAT | GO:0033673~negative regulation of kinase activity |  |  |  | 86 | 0.00299586 |
| GOTERM_BP_FAT | GO:0071216~cellular response to biotic stimulus |  |  |  | 81 | 0.03108214 |
| GOTERM_BP_FAT | GO:0045861~negative regulation of proteolysis |  |  |  | 73 | 0.04320231 |
| GOTERM_BP_FAT | GO:0031668~cellular response to extracellular stimulus |  |  |  | 70 | 0.00868136 |
| GOTERM_BP_FAT | GO:0035023~regulation of Rho protein signal transduction |  |  |  | 70 | 0.00868136 |
| GOTERM_BP_FAT | GO:0043409~negative regulation of MAPK cascade |  |  |  | 70 | 0.0269795 |
| GOTERM_BP_FAT | GO:0042594~response to starvation |  |  |  | 57 | 0.02134667 |
| GOTERM_BP_FAT | GO:2001251~negative regulation of chromosome organization |  |  |  | 57 | 0.04500444 |
| GOTERM_BP_FAT | GO:0009267~cellular response to starvation |  |  |  | 52 | 0.02507674 |
| GOTERM_BP_FAT | GO:0051302~regulation of cell division |  |  |  | 51 | 0.04481857 |
| GOTERM_BP_FAT | GO:0006352~DNA-templated transcription, initiation |  |  |  | 51 | 0.04481857 |
| GOTERM_BP_FAT | GO:0071901~negative regulation of protein serine/threonine kinase activity |  |  |  | 50 | 0.0156973 |
| GOTERM_BP_FAT | GO:0031330~negative regulation of cellular catabolic process |  |  |  | 42 | 0.01288339 |
| GOTERM_BP_FAT | GO:0008625~extrinsic apoptotic signaling pathway via death domain receptors |  |  |  | 40 | 0.01200512 |
| GOTERM_BP_FAT | GO:2001238~positive regulation of extrinsic apoptotic signaling pathway |  |  |  | 36 | 0.01002916 |
| GOTERM_BP_FAT | GO:0007266~Rho protein signal transduction |  |  |  | 36 | 0.01836009 |
| GOTERM_BP_FAT | GO:0033045~regulation of sister chromatid segregation |  |  |  | 34 | 0.02987551 |
| GOTERM_BP_FAT | GO:1901800~positive regulation of proteasomal protein catabolic process |  |  |  | 34 | 0.02987551 |
| GOTERM_BP_FAT | GO:1902117~positive regulation of organelle assembly |  |  |  | 34 | 0.04905233 |
| GOTERM_BP_FAT | GO:0036473~cell death in response to oxidative stress |  |  |  | 34 | 0.04905233 |
| GOTERM_BP_FAT | GO:0043966~histone H3 acetylation |  |  |  | 33 | 0.02194482 |
| GOTERM_BP_FAT | GO:0001738~morphogenesis of a polarized epithelium |  |  |  | 32 | 0.02818746 |
| GOTERM_BP_FAT | GO:0032436~positive regulation of proteasomal ubiquitin-dependent protein catabolic process |  |  |  | 31 | 0.02021542 |
| GOTERM_BP_FAT | GO:0043407~negative regulation of MAP kinase activity |  |  |  | 31 | 0.03599885 |
| GOTERM_BP_FAT | GO:1902041~regulation of extrinsic apoptotic signaling pathway via death domain receptors |  |  |  | 30 | 0.01382552 |
| GOTERM_BP_FAT | GO:0033047~regulation of mitotic sister chromatid segregation |  |  |  | 30 | 0.0262512 |
| GOTERM_BP_FAT | GO:0071426~ribonucleoprotein complex export from nucleus |  |  |  | 26 | 0.02154221 |
| GOTERM_BP_FAT | GO:0006509~membrane protein ectodomain proteolysis |  |  |  | 26 | 0.02154221 |
| GOTERM_BP_FAT | GO:1903051~negative regulation of proteolysis involved in cellular protein catabolic process |  |  |  | 26 | 0.04081863 |
| GOTERM_BP_FAT | GO:0010332~response to gamma radiation |  |  |  | 24 | 0.01873414 |
| GOTERM_BP_FAT | GO:0002753~cytoplasmic pattern recognition receptor signaling pathway |  |  |  | 20 | 0.02944865 |
| GOTERM_BP_FAT | GO:0090307~mitotic spindle assembly |  |  |  | 18 | 0.00218276 |
| GOTERM_BP_FAT | GO:1902850~microtubule cytoskeleton organization involved in mitosis |  |  |  | 18 | 0.00218276 |
| GOTERM_BP_FAT | GO:0034067~protein localization to Golgi apparatus |  |  |  | 18 | 0.02427755 |
| GOTERM_BP_FAT | GO:0034453~microtubule anchoring |  |  |  | 16 | 0.01835304 |
| GOTERM_BP_FAT | GO:0031122~cytoplasmic microtubule organization |  |  |  | 16 | 0.04691129 |
| GOTERM_BP_FAT | GO:0006298~mismatch repair |  |  |  | 16 | 0.04691129 |
| GOTERM_BP_FAT | GO:0032543~mitochondrial translation |  |  |  | 16 | 0.04691129 |
| GOTERM_BP_FAT | GO:0006359~regulation of transcription from RNA polymerase III promoter |  |  |  | 15 | 0.02648777 |
| GOTERM_BP_FAT | GO:0050687~negative regulation of defense response to virus |  |  |  | 12 | 0.02683202 |
| GOTERM_BP_FAT | GO:0061462~protein localization to lysosome |  |  |  | 12 | 0.02683202 |
| GOTERM_BP_FAT | GO:0072666~establishment of protein localization to vacuole |  |  |  | 11 | 0.04013141 |
| GOTERM_BP_FAT | GO:0072600~establishment of protein localization to Golgi |  |  |  | 11 | 0.04013141 |
| GOTERM_CC_FAT | GO:0043230~extracellular organelle | 1345 | 0.00023934 |  | 1314 | 0.00994738 |
| GOTERM_CC_FAT | GO:1903561~extracellular vesicle | 1344 | 0.00025779 |  | 1314 | 0.009095 |
| GOTERM_CC_FAT | GO:0070062~extracellular exosome | 1340 | 0.0001185 |  | 1309 | 0.00619276 |
| GOTERM_CC_FAT | GO:0005654~nucleoplasm | 1302 | 1.9679E-49 |  | 1308 | 1.6483E-54 |
| GOTERM_CC_FAT | GO:0005739~mitochondrion | 808 | 2.0268E-06 |  | 799 | 6.8322E-06 |
| GOTERM_CC_FAT | GO:0005829~cytosol | 580 | 1.5975E-09 |  | 572 | 1.4298E-08 |
| GOTERM_CC_FAT | GO:0015630~microtubule cytoskeleton | 542 | 1.4693E-14 |  | 537 | 5.836E-14 |
| GOTERM_CC_FAT | GO:0005794~Golgi apparatus | 528 | 1.6518E-09 |  | 528 | 3.3161E-10 |
| GOTERM_CC_FAT | GO:0005783~endoplasmic reticulum | 505 | 0.0008171 |  | 507 | 0.00017226 |
| GOTERM_CC_FAT | GO:0005694~chromosome | 441 | 5.5725E-08 |  | 444 | 2.9429E-09 |
| GOTERM_CC_FAT | GO:0005773~vacuole | 437 | 1.2482E-08 |  | 438 | 1.8189E-09 |
| GOTERM_CC_FAT | GO:0005730~nucleolus | 416 | 1.3954E-18 |  | 416 | 2.1596E-19 |
| GOTERM_CC_FAT | GO:0044427~chromosomal part | 403 | 3.4887E-06 |  | 406 | 2.7667E-07 |
| GOTERM_CC_FAT | GO:0030529~intracellular ribonucleoprotein complex | 392 | 2.7192E-09 |  | 391 | 1.3385E-09 |
| GOTERM_CC_FAT | GO:1990904~ribonucleoprotein complex | 392 | 2.7192E-09 |  | 391 | 1.3385E-09 |
| GOTERM_CC_FAT | GO:0031975~envelope | 390 | 4.9208E-06 |  | 391 | 1.0674E-06 |
| GOTERM_CC_FAT | GO:0044451~nucleoplasm part | 387 | 6.5783E-23 |  | 393 | 7.0345E-27 |
| GOTERM_CC_FAT | GO:0031967~organelle envelope | 385 | 9.9815E-06 |  | 386 | 2.278E-06 |
| GOTERM_CC_FAT | GO:0005815~microtubule organizing center | 348 | 4.987E-16 |  | 345 | 1.6072E-15 |
| GOTERM_CC_FAT | GO:0005813~centrosome | 307 | 4.507E-15 |  | 305 | 7.6988E-15 |
| GOTERM_CC_FAT | GO:0044429~mitochondrial part | 286 | 0.00566148 |  | 286 | 0.00318828 |
| GOTERM_CC_FAT | GO:0005768~endosome | 283 | 6.2234E-05 |  | 282 | 4.5704E-05 |
| GOTERM_CC_FAT | GO:0070161~anchoring junction | 251 | 2.0019E-06 |  | 248 | 5.1117E-06 |
| GOTERM_CC_FAT | GO:0000228~nuclear chromosome | 250 | 6.6753E-07 |  | 252 | 7.3536E-08 |
| GOTERM_CC_FAT | GO:0005912~adherens junction | 243 | 1.0444E-06 |  | 240 | 2.9051E-06 |
| GOTERM_CC_FAT | GO:0044432~endoplasmic reticulum part | 235 | 0.03085982 |  | 238 | 0.00823537 |
| GOTERM_CC_FAT | GO:0044454~nuclear chromosome part | 230 | 1.1223E-05 |  | 232 | 1.5669E-06 |
| GOTERM_CC_FAT | GO:0030055~cell-substrate junction | 223 | 1.9805E-07 |  | 220 | 7.0046E-07 |
| GOTERM_CC_FAT | GO:0005740~mitochondrial envelope | 221 | 0.03806578 |  | 220 | 0.03393158 |
| GOTERM_CC_FAT | GO:0005925~focal adhesion | 220 | 7.9513E-08 |  | 217 | 3.0571E-07 |
| GOTERM_CC_FAT | GO:0005924~cell-substrate adherens junction | 220 | 3.6876E-07 |  | 217 | 1.28E-06 |
| GOTERM_CC_FAT | GO:0044431~Golgi apparatus part | 214 | 3.7799E-05 |  | 213 | 3.3713E-05 |
| GOTERM_CC_FAT | GO:0031966~mitochondrial membrane | 210 | 0.02024024 |  | 211 | 0.00963159 |
| GOTERM_CC_FAT | GO:0044437~vacuolar part | 206 | 1.9688E-07 |  | 205 | 1.8753E-07 |
| GOTERM_CC_FAT | GO:0000323~lytic vacuole | 195 | 1.2694E-05 |  | 195 | 6.4613E-06 |
| GOTERM_CC_FAT | GO:0005774~vacuolar membrane | 194 | 9.0193E-07 |  | 192 | 1.8134E-06 |
| GOTERM_CC_FAT | GO:0005764~lysosome | 194 | 1.5321E-05 |  | 194 | 7.8557E-06 |
| GOTERM_CC_FAT | GO:0019866~organelle inner membrane | 172 | 0.00597509 |  | 172 | 0.00389722 |
| GOTERM_CC_FAT | GO:0005635~nuclear envelope | 169 | 4.887E-06 |  | 171 | 5.545E-07 |
| GOTERM_CC_FAT | GO:0098687~chromosomal region | 165 | 4.0741E-10 |  | 166 | 5.838E-11 |
| GOTERM_CC_FAT | GO:0005743~mitochondrial inner membrane | 158 | 0.00729295 |  | 158 | 0.00489355 |
| GOTERM_CC_FAT | GO:0016604~nuclear body | 157 | 1.3129E-13 |  | 158 | 1.205E-14 |
| GOTERM_CC_FAT | GO:0061695~transferase complex, transferring phosphorus-containing groups | 132 | 1.5933E-07 |  | 133 | 3.027E-08 |
| GOTERM_CC_FAT | GO:0005819~spindle | 131 | 3.1441E-05 |  | 128 | 0.00016341 |
| GOTERM_CC_FAT | GO:0000790~nuclear chromatin | 130 | 0.00299934 |  | 132 | 0.00066908 |
| GOTERM_CC_FAT | GO:0005874~microtubule | 125 | 0.00070603 |  | 128 | 6.1542E-05 |
| GOTERM_CC_FAT | GO:0000151~ubiquitin ligase complex | 122 | 7.7872E-06 |  | 123 | 1.8852E-06 |
| GOTERM_CC_FAT | GO:0005765~lysosomal membrane | 118 | 2.9969E-06 |  | 116 | 1.0798E-05 |
| GOTERM_CC_FAT | GO:0098852~lytic vacuole membrane | 118 | 2.9969E-06 |  | 116 | 1.0798E-05 |
| GOTERM_CC_FAT | GO:0031965~nuclear membrane | 115 | 7.0182E-05 |  | 115 | 4.4415E-05 |
| GOTERM_CC_FAT | GO:0000139~Golgi membrane | 113 | 0.00787603 |  | 110 | 0.02262668 |
| GOTERM_CC_FAT | GO:0005769~early endosome | 102 | 0.0025526 |  | 101 | 0.00329344 |
| GOTERM_CC_FAT | GO:0044440~endosomal part | 99 | 0.00436634 |  | 98 | 0.00559518 |
| GOTERM_CC_FAT | GO:0031984~organelle subcompartment | 98 | 0.01512503 |  | 99 | 0.00683122 |
| GOTERM_CC_FAT | GO:0005681~spliceosomal complex | 94 | 2.1618E-09 |  | 94 | 1.2527E-09 |
| GOTERM_CC_FAT | GO:0000775~chromosome, centromeric region | 92 | 1.1632E-07 |  | 93 | 1.789E-08 |
| GOTERM_CC_FAT | GO:0098791~Golgi subcompartment | 92 | 0.02690322 |  | 93 | 0.01290292 |
| GOTERM_CC_FAT | GO:0005759~mitochondrial matrix | 89 | 0.00341377 |  | 90 | 0.00128946 |
| GOTERM_CC_FAT | GO:0010008~endosome membrane | 86 | 0.03485327 |  | 85 | 0.04298002 |
| GOTERM_CC_FAT | GO:0005770~late endosome | 83 | 0.00104726 |  | 83 | 0.00075783 |
| GOTERM_CC_FAT | GO:0000428~DNA-directed RNA polymerase complex | 78 | 1.9166E-05 |  | 80 | 1.2137E-06 |
| GOTERM_CC_FAT | GO:0030880~RNA polymerase complex | 78 | 1.9166E-05 |  | 80 | 1.2137E-06 |
| GOTERM_CC_FAT | GO:0055029~nuclear DNA-directed RNA polymerase complex | 78 | 1.9166E-05 |  | 80 | 1.2137E-06 |
| GOTERM_CC_FAT | GO:0000781~chromosome, telomeric region | 77 | 0.00011338 |  | 76 | 0.00020547 |
| GOTERM_CC_FAT | GO:0031461~cullin-RING ubiquitin ligase complex | 77 | 0.00042153 |  | 77 | 0.00030385 |
| GOTERM_CC_FAT | GO:0044450~microtubule organizing center part | 77 | 0.00133569 |  | 75 | 0.00418637 |
| GOTERM_CC_FAT | GO:0030139~endocytic vesicle | 72 | 0.00388903 |  | 70 | 0.01113707 |
| GOTERM_CC_FAT | GO:0030135~coated vesicle | 71 | 0.00026715 |  | 68 | 0.00252731 |
| GOTERM_CC_FAT | GO:0098798~mitochondrial protein complex | 69 | 0.01682775 |  | 69 | 0.01342229 |
| GOTERM_CC_FAT | GO:0030496~midbody | 68 | 0.00191345 |  | 67 | 0.00315441 |
| GOTERM_CC_FAT | GO:0000776~kinetochore | 66 | 2.6227E-07 |  | 68 | 4.6135E-09 |
| GOTERM_CC_FAT | GO:0048475~coated membrane | 66 | 2.0541E-05 |  | 66 | 1.4586E-05 |
| GOTERM_CC_FAT | GO:0030117~membrane coat | 66 | 2.0541E-05 |  | 66 | 1.4586E-05 |
| GOTERM_CC_FAT | GO:0031248~protein acetyltransferase complex | 65 | 6.6505E-05 |  | 66 | 1.4586E-05 |
| GOTERM_CC_FAT | GO:1902493~acetyltransferase complex | 65 | 6.6505E-05 |  | 66 | 1.4586E-05 |
| GOTERM_CC_FAT | GO:0071013~catalytic step 2 spliceosome | 64 | 5.3656E-06 |  | 64 | 3.7753E-06 |
| GOTERM_CC_FAT | GO:0016591~DNA-directed RNA polymerase II, holoenzyme | 64 | 0.00019618 |  | 65 | 4.813E-05 |
| GOTERM_CC_FAT | GO:0000784~nuclear chromosome, telomeric region | 63 | 0.00103347 |  | 62 | 0.00187289 |
| GOTERM_CC_FAT | GO:0000123~histone acetyltransferase complex | 61 | 1.4121E-05 |  | 62 | 2.463E-06 |
| GOTERM_CC_FAT | GO:0000922~spindle pole | 60 | 0.00114063 |  | 58 | 0.00475826 |
| GOTERM_CC_FAT | GO:0016607~nuclear speck | 59 | 0.00074834 |  | 59 | 0.00057184 |
| GOTERM_CC_FAT | GO:0034708~methyltransferase complex | 58 | 0.00021707 |  | 58 | 0.00016325 |
| GOTERM_CC_FAT | GO:0005814~centriole | 55 | 0.00207702 |  | 53 | 0.00856604 |
| GOTERM_CC_FAT | GO:0070603~SWI/SNF superfamily-type complex | 50 | 0.00378583 |  | 49 | 0.00715012 |
| GOTERM_CC_FAT | GO:0016605~PML body | 49 | 8.6245E-05 |  | 49 | 6.6038E-05 |
| GOTERM_CC_FAT | GO:0036064~ciliary basal body | 49 | 0.03736938 |  | 50 | 0.01640484 |
| GOTERM_CC_FAT | GO:0035097~histone methyltransferase complex | 48 | 0.00011908 |  | 48 | 9.1836E-05 |
| GOTERM_CC_FAT | GO:0034399~nuclear periphery | 48 | 0.00161527 |  | 48 | 0.00128927 |
| GOTERM_CC_FAT | GO:1902911~protein kinase complex | 45 | 0.01243813 |  | 44 | 0.02209197 |
| GOTERM_CC_FAT | GO:0005657~replication fork | 41 | 4.0628E-05 |  | 42 | 5.0635E-06 |
| GOTERM_CC_FAT | GO:0000502~proteasome complex | 41 | 0.00013974 |  | 41 | 0.00011073 |
| GOTERM_CC_FAT | GO:1902554~serine/threonine protein kinase complex | 39 | 0.01602946 |  | 38 | 0.02986421 |
| GOTERM_CC_FAT | GO:0016363~nuclear matrix | 38 | 0.0055935 |  | 38 | 0.00468378 |
| GOTERM_CC_FAT | GO:0015935~small ribosomal subunit | 35 | 0.00266618 |  | 36 | 0.00061293 |
| GOTERM_CC_FAT | GO:0030118~clathrin coat | 34 | 0.00013655 |  | 33 | 0.00056968 |
| GOTERM_CC_FAT | GO:0005798~Golgi-associated vesicle | 34 | 0.01597758 |  | 35 | 0.00512234 |
| GOTERM_CC_FAT | GO:0030119~AP-type membrane coat adaptor complex | 33 | 0.00068667 |  | 33 | 0.00056968 |
| GOTERM_CC_FAT | GO:0005776~autophagosome | 33 | 0.02053502 |  | 33 | 0.01780248 |
| GOTERM_CC_FAT | GO:0008023~transcription elongation factor complex | 32 | 0.006476 |  | 32 | 0.00553253 |
| GOTERM_CC_FAT | GO:0030662~coated vesicle membrane | 30 | 0.00189362 |  | 29 | 0.0058711 |
| GOTERM_CC_FAT | GO:0005876~spindle microtubule | 30 | 0.00501891 |  | 30 | 0.00430105 |
| GOTERM_CC_FAT | GO:0000152~nuclear ubiquitin ligase complex | 29 | 0.02982146 |  | 30 | 0.0099243 |
| GOTERM_CC_FAT | GO:0005643~nuclear pore | 28 | 0.00031105 |  | 28 | 0.00026151 |
| GOTERM_CC_FAT | GO:0030120~vesicle coat | 28 | 0.00031105 |  | 27 | 0.00147532 |
| GOTERM_CC_FAT | GO:0015030~Cajal body | 28 | 0.00120975 |  | 28 | 0.00102854 |
| GOTERM_CC_FAT | GO:0008287~protein serine/threonine phosphatase complex | 28 | 0.03820247 |  | 28 | 0.03391261 |
| GOTERM_CC_FAT | GO:1903293~phosphatase complex | 28 | 0.03820247 |  | 28 | 0.03391261 |
| GOTERM_CC_FAT | GO:0044452~nucleolar part | 26 | 0.00696202 |  | 26 | 0.0060702 |
| GOTERM_CC_FAT | GO:0005881~cytoplasmic microtubule | 26 | 0.01648027 |  | 27 | 0.0043794 |
| GOTERM_CC_FAT | GO:0005788~endoplasmic reticulum lumen | 25 | 0.00345893 |  | 23 | 0.03425004 |
| GOTERM_CC_FAT | GO:0005801~cis-Golgi network | 25 | 0.00345893 |  | 24 | 0.01150239 |
| GOTERM_CC_FAT | GO:0030532~small nuclear ribonucleoprotein complex | 25 | 0.00953927 |  | 25 | 0.00837601 |
| GOTERM_CC_FAT | GO:0008180~COP9 signalosome | 25 | 0.02191009 |  | 25 | 0.01944392 |
| GOTERM_CC_FAT | GO:0035861~site of double-strand break | 24 | 0.00029194 |  | 24 | 0.0002498 |
| GOTERM_CC_FAT | GO:0030990~intraciliary transport particle | 24 | 0.00142928 |  | 25 | 0.00016399 |
| GOTERM_CC_FAT | GO:0043596~nuclear replication fork | 22 | 0.00301302 |  | 23 | 0.00037992 |
| GOTERM_CC_FAT | GO:0030131~clathrin adaptor complex | 22 | 0.00955502 |  | 21 | 0.02884231 |
| GOTERM_CC_FAT | GO:0000779~condensed chromosome, centromeric region | 22 | 0.04970779 |  | 23 | 0.01571492 |
| GOTERM_CC_FAT | GO:0030660~Golgi-associated vesicle membrane | 21 | 0.00435307 |  | 21 | 0.0038481 |
| GOTERM_CC_FAT | GO:0031463~Cul3-RING ubiquitin ligase complex | 21 | 0.01328668 |  | 21 | 0.01187165 |
| GOTERM_CC_FAT | GO:0097525~spliceosomal snRNP complex | 21 | 0.03194499 |  | 21 | 0.02884231 |
| GOTERM_CC_FAT | GO:0022627~cytosolic small ribosomal subunit | 21 | 0.03194499 |  | 21 | 0.02884231 |
| GOTERM_CC_FAT | GO:0030894~replisome | 18 | 0.00336273 |  | 18 | 0.00300458 |
| GOTERM_CC_FAT | GO:0071339~MLL1 complex | 17 | 0.01821931 |  | 17 | 0.01656935 |
| GOTERM_CC_FAT | GO:0005839~proteasome core complex | 17 | 0.01821931 |  | 17 | 0.01656935 |
| GOTERM_CC_FAT | GO:0044665~MLL1/2 complex | 17 | 0.01821931 |  | 17 | 0.01656935 |
| GOTERM_CC_FAT | GO:0080008~Cul4-RING E3 ubiquitin ligase complex | 17 | 0.04699265 |  | 17 | 0.04317471 |
| GOTERM_CC_FAT | GO:0043601~nuclear replisome | 16 | 0.00745282 |  | 16 | 0.00675528 |
| GOTERM_CC_FAT | GO:1990752~microtubule end | 15 | 0.03617645 |  | 15 | 0.03336556 |
| GOTERM_CC_FAT | GO:0030992~intraciliary transport particle B | 14 | 0.01630419 |  | 14 | 0.01499097 |
| GOTERM_CC_FAT | GO:0005732~small nucleolar ribonucleoprotein complex | 13 | 0.02397296 |  | 13 | 0.02219956 |
| GOTERM_CC_FAT | GO:0005671~Ada2/Gcn5/Ada3 transcription activator complex | 12 | 0.0350848 |  | 12 | 0.0327208 |
| GOTERM_CC_FAT | GO:0005662~DNA replication factor A complex | 12 | 0.0350848 |  | 12 | 0.0327208 |
| GOTERM_CC_FAT | GO:0031988~membrane-bounded vesicle | 1527 | 0.0066643 |  |  |  |
| GOTERM_CC_FAT | GO:0015629~actin cytoskeleton | 198 | 0.04409689 |  |  |  |
| GOTERM_CC_FAT | GO:0043209~myelin sheath | 96 | 0.04820933 |  |  |  |
| GOTERM_CC_FAT | GO:0030136~clathrin-coated vesicle | 46 | 0.01702056 |  |  |  |
| GOTERM_CC_FAT | GO:0000792~heterochromatin | 44 | 0.04122993 |  |  |  |
| GOTERM_CC_FAT | GO:0005905~clathrin-coated pit | 25 | 0.04362937 |  |  |  |
| GOTERM_CC_FAT | GO:0090544~BAF-type complex | 18 | 0.03458714 |  |  |  |
| GOTERM_CC_FAT | GO:0030125~clathrin vesicle coat | 12 | 0.0350848 |  |  |  |
| GOTERM_CC_FAT | GO:0042175~nuclear outer membrane-endoplasmic reticulum membrane network |  |  |  | 211 | 0.02214028 |
| GOTERM_CC_FAT | GO:0005789~endoplasmic reticulum membrane |  |  |  | 201 | 0.04781315 |
| GOTERM_CC_FAT | GO:0098589~membrane region |  |  |  | 123 | 0.01157631 |
| GOTERM_CC_FAT | GO:0098857~membrane microdomain |  |  |  | 97 | 0.04363645 |
| GOTERM_CC_FAT | GO:0045121~membrane raft |  |  |  | 97 | 0.04363645 |
| GOTERM_CC_FAT | GO:0000793~condensed chromosome |  |  |  | 75 | 0.04157802 |
| GOTERM_CC_FAT | GO:0042641~actomyosin |  |  |  | 37 | 0.01212332 |
| GOTERM_CC_FAT | GO:0097517~contractile actin filament bundle |  |  |  | 31 | 0.0293616 |
| GOTERM_CC_FAT | GO:0005795~Golgi stack |  |  |  | 31 | 0.0293616 |
| GOTERM_CC_FAT | GO:0001725~stress fiber |  |  |  | 31 | 0.0293616 |
| GOTERM_CC_FAT | GO:0016592~mediator complex |  |  |  | 28 | 0.00797868 |
| GOTERM_CC_FAT | GO:0000777~condensed chromosome kinetochore |  |  |  | 19 | 0.02291554 |
| GOTERM_CC_FAT | GO:0030137~COPI-coated vesicle |  |  |  | 12 | 0.0327208 |
| GOTERM_CC_FAT | GO:0000145~exocyst |  |  |  | 12 | 0.0327208 |
| GOTERM_CC_FAT | GO:0032797~SMN complex |  |  |  | 11 | 0.04796939 |
| GOTERM_CC_FAT | GO:0030897~HOPS complex |  |  |  | 11 | 0.04796939 |
| GOTERM_MF_FAT | GO:0097159~organic cyclic compound binding | 2831 | 1.3349E-51 |  | 2811 | 1.4471E-52 |
| GOTERM_MF_FAT | GO:1901363~heterocyclic compound binding | 2814 | 2.5625E-52 |  | 2792 | 1.0805E-52 |
| GOTERM_MF_FAT | GO:0003676~nucleic acid binding | 1863 | 1.2363E-37 |  | 1851 | 2.0942E-38 |
| GOTERM_MF_FAT | GO:0043167~ion binding | 1563 | 7.6182E-07 |  | 1552 | 3.4365E-07 |
| GOTERM_MF_FAT | GO:0043169~cation binding | 1503 | 8.049E-07 |  | 1494 | 2.6429E-07 |
| GOTERM_MF_FAT | GO:0046872~metal ion binding | 1497 | 3.0336E-07 |  | 1490 | 5.6581E-08 |
| GOTERM_MF_FAT | GO:0036094~small molecule binding | 1373 | 9.1267E-35 |  | 1353 | 3.3178E-32 |
| GOTERM_MF_FAT | GO:0000166~nucleotide binding | 1312 | 1.225E-36 |  | 1293 | 4.8679E-34 |
| GOTERM_MF_FAT | GO:1901265~nucleoside phosphate binding | 1312 | 1.225E-36 |  | 1293 | 4.8679E-34 |
| GOTERM_MF_FAT | GO:0097367~carbohydrate derivative binding | 1145 | 4.8339E-24 |  | 1126 | 1.062E-21 |
| GOTERM_MF_FAT | GO:0032553~ribonucleotide binding | 1029 | 2.8625E-25 |  | 1016 | 5.889E-24 |
| GOTERM_MF_FAT | GO:0017076~purine nucleotide binding | 1028 | 3.4429E-26 |  | 1016 | 4.2875E-25 |
| GOTERM_MF_FAT | GO:0032555~purine ribonucleotide binding | 1024 | 8.5583E-26 |  | 1012 | 1.0564E-24 |
| GOTERM_MF_FAT | GO:0001882~nucleoside binding | 1022 | 8.3582E-27 |  | 1010 | 1.1455E-25 |
| GOTERM_MF_FAT | GO:0032549~ribonucleoside binding | 1019 | 1.119E-26 |  | 1007 | 1.5475E-25 |
| GOTERM_MF_FAT | GO:0001883~purine nucleoside binding | 1017 | 1.7763E-26 |  | 1005 | 2.4451E-25 |
| GOTERM_MF_FAT | GO:0032550~purine ribonucleoside binding | 1016 | 2.2367E-26 |  | 1004 | 3.0717E-25 |
| GOTERM_MF_FAT | GO:0035639~purine ribonucleoside triphosphate binding | 1012 | 2.523E-26 |  | 1000 | 3.5434E-25 |
| GOTERM_MF_FAT | GO:0003723~RNA binding | 908 | 6.8189E-59 |  | 909 | 3.1841E-62 |
| GOTERM_MF_FAT | GO:0030554~adenyl nucleotide binding | 844 | 3.4466E-29 |  | 831 | 4.9671E-27 |
| GOTERM_MF_FAT | GO:0032559~adenyl ribonucleotide binding | 841 | 7.5317E-29 |  | 828 | 1.0643E-26 |
| GOTERM_MF_FAT | GO:0005524~ATP binding | 830 | 1.9331E-28 |  | 817 | 2.8566E-26 |
| GOTERM_MF_FAT | GO:0044822~poly(A) RNA binding | 722 | 4.9818E-59 |  | 724 | 1.2794E-62 |
| GOTERM_MF_FAT | GO:0046914~transition metal ion binding | 687 | 5.1627E-05 |  | 684 | 2.2038E-05 |
| GOTERM_MF_FAT | GO:0008270~zinc ion binding | 589 | 1.0976E-08 |  | 584 | 1.1682E-08 |
| GOTERM_MF_FAT | GO:0098772~molecular function regulator | 477 | 1.4987E-06 |  | 470 | 4.7384E-06 |
| GOTERM_MF_FAT | GO:0016301~kinase activity | 466 | 2.6041E-11 |  | 446 | 1.0719E-07 |
| GOTERM_MF_FAT | GO:0016773~phosphotransferase activity, alcohol group as acceptor | 439 | 2.3259E-13 |  | 421 | 1.4704E-09 |
| GOTERM_MF_FAT | GO:0016817~hydrolase activity, acting on acid anhydrides | 383 | 3.3692E-06 |  | 384 | 5.4882E-07 |
| GOTERM_MF_FAT | GO:0004672~protein kinase activity | 380 | 4.9758E-13 |  | 364 | 2.2203E-09 |
| GOTERM_MF_FAT | GO:0016818~hydrolase activity, acting on acid anhydrides, in phosphorus-containing anhydrides | 378 | 4.401E-06 |  | 378 | 1.1531E-06 |
| GOTERM_MF_FAT | GO:0016462~pyrophosphatase activity | 377 | 3.0392E-06 |  | 377 | 7.8295E-07 |
| GOTERM_MF_FAT | GO:0017111~nucleoside-triphosphatase activity | 359 | 6.8557E-07 |  | 360 | 1.0384E-07 |
| GOTERM_MF_FAT | GO:0030234~enzyme regulator activity | 333 | 0.00018905 |  | 328 | 0.00040598 |
| GOTERM_MF_FAT | GO:0044877~macromolecular complex binding | 273 | 2.4984E-10 |  | 272 | 1.2477E-10 |
| GOTERM_MF_FAT | GO:0004674~protein serine/threonine kinase activity | 230 | 2.4305E-12 |  | 223 | 2.7568E-10 |
| GOTERM_MF_FAT | GO:0000988~transcription factor activity, protein binding | 217 | 6.5118E-06 |  | 216 | 4.4738E-06 |
| GOTERM_MF_FAT | GO:0003682~chromatin binding | 214 | 4.1247E-08 |  | 211 | 1.1347E-07 |
| GOTERM_MF_FAT | GO:0000989~transcription factor activity, transcription factor binding | 214 | 1.1834E-05 |  | 213 | 8.2633E-06 |
| GOTERM_MF_FAT | GO:0016887~ATPase activity | 200 | 3.2637E-05 |  | 199 | 2.4238E-05 |
| GOTERM_MF_FAT | GO:0042578~phosphoric ester hydrolase activity | 195 | 0.00011591 |  | 191 | 0.00037364 |
| GOTERM_MF_FAT | GO:0019787~ubiquitin-like protein transferase activity | 193 | 1.5887E-09 |  | 193 | 5.1846E-10 |
| GOTERM_MF_FAT | GO:0003712~transcription cofactor activity | 192 | 6.341E-06 |  | 192 | 2.6191E-06 |
| GOTERM_MF_FAT | GO:0004842~ubiquitin-protein transferase activity | 184 | 6.2486E-09 |  | 184 | 2.1748E-09 |
| GOTERM_MF_FAT | GO:0008047~enzyme activator activity | 167 | 2.2449E-06 |  | 161 | 4.6907E-05 |
| GOTERM_MF_FAT | GO:0042623~ATPase activity, coupled | 149 | 1.6822E-05 |  | 150 | 4.0467E-06 |
| GOTERM_MF_FAT | GO:0016791~phosphatase activity | 147 | 0.00018615 |  | 143 | 0.0008546 |
| GOTERM_MF_FAT | GO:0016746~transferase activity, transferring acyl groups | 130 | 0.00326922 |  | 129 | 0.00320051 |
| GOTERM_MF_FAT | GO:0016874~ligase activity | 124 | 1.1626E-07 |  | 122 | 3.5414E-07 |
| GOTERM_MF_FAT | GO:0060589~nucleoside-triphosphatase regulator activity | 117 | 3.0039E-06 |  | 115 | 8.0776E-06 |
| GOTERM_MF_FAT | GO:0005085~guanyl-nucleotide exchange factor activity | 117 | 4.0077E-05 |  | 117 | 2.1779E-05 |
| GOTERM_MF_FAT | GO:0016741~transferase activity, transferring one-carbon groups | 114 | 5.2332E-05 |  | 114 | 2.8916E-05 |
| GOTERM_MF_FAT | GO:0003713~transcription coactivator activity | 113 | 8.9791E-06 |  | 111 | 2.346E-05 |
| GOTERM_MF_FAT | GO:0016747~transferase activity, transferring acyl groups other than amino-acyl groups | 109 | 0.00489959 |  | 108 | 0.00522258 |
| GOTERM_MF_FAT | GO:0008168~methyltransferase activity | 107 | 7.1074E-05 |  | 107 | 4.0476E-05 |
| GOTERM_MF_FAT | GO:0035091~phosphatidylinositol binding | 107 | 7.1074E-05 |  | 101 | 0.00204742 |
| GOTERM_MF_FAT | GO:0030695~GTPase regulator activity | 106 | 3.396E-05 |  | 104 | 8.7211E-05 |
| GOTERM_MF_FAT | GO:0004721~phosphoprotein phosphatase activity | 104 | 6.5382E-06 |  | 104 | 3.5498E-06 |
| GOTERM_MF_FAT | GO:0000287~magnesium ion binding | 104 | 0.00081589 |  | 103 | 0.00093029 |
| GOTERM_MF_FAT | GO:0008234~cysteine-type peptidase activity | 101 | 0.00012147 |  | 99 | 0.00029831 |
| GOTERM_MF_FAT | GO:0004518~nuclease activity | 97 | 1.1798E-06 |  | 98 | 2.2246E-07 |
| GOTERM_MF_FAT | GO:0003924~GTPase activity | 96 | 0.00805116 |  | 96 | 0.00549717 |
| GOTERM_MF_FAT | GO:0005096~GTPase activator activity | 92 | 3.4058E-05 |  | 90 | 0.00010431 |
| GOTERM_MF_FAT | GO:0005088~Ras guanyl-nucleotide exchange factor activity | 77 | 0.00104356 |  | 78 | 0.00032952 |
| GOTERM_MF_FAT | GO:0016853~isomerase activity | 76 | 0.001331 |  | 74 | 0.00347814 |
| GOTERM_MF_FAT | GO:0003729~mRNA binding | 75 | 3.615E-07 |  | 75 | 2.0991E-07 |
| GOTERM_MF_FAT | GO:0004713~protein tyrosine kinase activity | 75 | 0.00022971 |  | 72 | 0.00154105 |
| GOTERM_MF_FAT | GO:0004386~helicase activity | 72 | 4.3603E-07 |  | 72 | 2.5748E-07 |
| GOTERM_MF_FAT | GO:0016779~nucleotidyltransferase activity | 71 | 8.8559E-08 |  | 71 | 5.1468E-08 |
| GOTERM_MF_FAT | GO:0008757~S-adenosylmethionine-dependent methyltransferase activity | 71 | 3.4099E-05 |  | 71 | 2.1684E-05 |
| GOTERM_MF_FAT | GO:0019783~ubiquitin-like protein-specific protease activity | 67 | 0.00021902 |  | 67 | 0.00014668 |
| GOTERM_MF_FAT | GO:0019207~kinase regulator activity | 66 | 0.00089641 |  | 64 | 0.00283682 |
| GOTERM_MF_FAT | GO:0061659~ubiquitin-like protein ligase activity | 66 | 0.01780904 |  | 67 | 0.0075293 |
| GOTERM_MF_FAT | GO:0061630~ubiquitin protein ligase activity | 63 | 0.03149414 |  | 64 | 0.01426809 |
| GOTERM_MF_FAT | GO:0004725~protein tyrosine phosphatase activity | 62 | 0.00153874 |  | 63 | 0.00047733 |
| GOTERM_MF_FAT | GO:0101005~ubiquitinyl hydrolase activity | 61 | 0.00118789 |  | 61 | 0.0008432 |
| GOTERM_MF_FAT | GO:0036459~thiol-dependent ubiquitinyl hydrolase activity | 61 | 0.00118789 |  | 61 | 0.0008432 |
| GOTERM_MF_FAT | GO:0016410~N-acyltransferase activity | 54 | 0.00046288 |  | 54 | 0.00033045 |
| GOTERM_MF_FAT | GO:0019887~protein kinase regulator activity | 54 | 0.01114671 |  | 52 | 0.02922572 |
| GOTERM_MF_FAT | GO:1901981~phosphatidylinositol phosphate binding | 54 | 0.02435827 |  | 53 | 0.03360521 |
| GOTERM_MF_FAT | GO:0004843~thiol-dependent ubiquitin-specific protease activity | 53 | 0.0006287 |  | 54 | 0.00016507 |
| GOTERM_MF_FAT | GO:0008094~DNA-dependent ATPase activity | 52 | 2.0535E-07 |  | 52 | 1.334E-07 |
| GOTERM_MF_FAT | GO:0016407~acetyltransferase activity | 52 | 1.8144E-05 |  | 52 | 1.2405E-05 |
| GOTERM_MF_FAT | GO:0004519~endonuclease activity | 51 | 0.00357819 |  | 53 | 0.0004532 |
| GOTERM_MF_FAT | GO:0008026~ATP-dependent helicase activity | 50 | 4.9223E-07 |  | 50 | 3.2619E-07 |
| GOTERM_MF_FAT | GO:0070035~purine NTP-dependent helicase activity | 50 | 4.9223E-07 |  | 50 | 3.2619E-07 |
| GOTERM_MF_FAT | GO:0043566~structure-specific DNA binding | 48 | 0.02840261 |  | 48 | 0.02280205 |
| GOTERM_MF_FAT | GO:0005057~receptor signaling protein activity | 48 | 0.02840261 |  | 49 | 0.01197136 |
| GOTERM_MF_FAT | GO:0008170~N-methyltransferase activity | 47 | 0.00025682 |  | 47 | 0.00018674 |
| GOTERM_MF_FAT | GO:0043021~ribonucleoprotein complex binding | 47 | 0.00054307 |  | 48 | 0.00013116 |
| GOTERM_MF_FAT | GO:0008276~protein methyltransferase activity | 47 | 0.00202971 |  | 47 | 0.00153145 |
| GOTERM_MF_FAT | GO:0008080~N-acetyltransferase activity | 46 | 8.9164E-06 |  | 46 | 6.2263E-06 |
| GOTERM_MF_FAT | GO:0004540~ribonuclease activity | 45 | 9.7633E-05 |  | 45 | 7.0607E-05 |
| GOTERM_MF_FAT | GO:0008565~protein transporter activity | 45 | 9.7633E-05 |  | 44 | 0.00024171 |
| GOTERM_MF_FAT | GO:0004527~exonuclease activity | 43 | 8.1677E-05 |  | 42 | 0.00021609 |
| GOTERM_MF_FAT | GO:0003697~single-stranded DNA binding | 41 | 0.000414 |  | 40 | 0.00097487 |
| GOTERM_MF_FAT | GO:0004812~aminoacyl-tRNA ligase activity | 37 | 1.033E-05 |  | 37 | 7.5916E-06 |
| GOTERM_MF_FAT | GO:0016876~ligase activity, forming aminoacyl-tRNA and related compounds | 37 | 1.033E-05 |  | 37 | 7.5916E-06 |
| GOTERM_MF_FAT | GO:0016875~ligase activity, forming carbon-oxygen bonds | 37 | 1.033E-05 |  | 37 | 7.5916E-06 |
| GOTERM_MF_FAT | GO:0003725~double-stranded RNA binding | 37 | 0.00074574 |  | 38 | 0.00015915 |
| GOTERM_MF_FAT | GO:0034212~peptide N-acetyltransferase activity | 36 | 0.00016977 |  | 36 | 0.00012918 |
| GOTERM_MF_FAT | GO:0016811~hydrolase activity, acting on carbon-nitrogen (but not peptide) bonds, in linear amides | 36 | 0.03807053 |  | 36 | 0.03186965 |
| GOTERM_MF_FAT | GO:0061733~peptide-lysine-N-acetyltransferase activity | 35 | 2.5044E-05 |  | 35 | 1.8772E-05 |
| GOTERM_MF_FAT | GO:0004402~histone acetyltransferase activity | 34 | 3.8833E-05 |  | 34 | 2.9396E-05 |
| GOTERM_MF_FAT | GO:0003684~damaged DNA binding | 33 | 0.00054721 |  | 33 | 0.00042879 |
| GOTERM_MF_FAT | GO:0019003~GDP binding | 33 | 0.00134846 |  | 33 | 0.00106959 |
| GOTERM_MF_FAT | GO:0004722~protein serine/threonine phosphatase activity | 33 | 0.00298856 |  | 32 | 0.00677728 |
| GOTERM_MF_FAT | GO:0016278~lysine N-methyltransferase activity | 31 | 0.00580054 |  | 31 | 0.00474713 |
| GOTERM_MF_FAT | GO:0016279~protein-lysine N-methyltransferase activity | 31 | 0.00580054 |  | 31 | 0.00474713 |
| GOTERM_MF_FAT | GO:0042054~histone methyltransferase activity | 31 | 0.01121839 |  | 31 | 0.00928981 |
| GOTERM_MF_FAT | GO:0003678~DNA helicase activity | 30 | 5.9688E-05 |  | 30 | 4.6422E-05 |
| GOTERM_MF_FAT | GO:0008408~3'-5' exonuclease activity | 30 | 0.00169664 |  | 30 | 0.00136886 |
| GOTERM_MF_FAT | GO:0004536~deoxyribonuclease activity | 30 | 0.04339824 |  | 32 | 0.00677728 |
| GOTERM_MF_FAT | GO:0008173~RNA methyltransferase activity | 29 | 0.00245004 |  | 29 | 0.00199595 |
| GOTERM_MF_FAT | GO:0003743~translation initiation factor activity | 29 | 0.02028674 |  | 30 | 0.00661961 |
| GOTERM_MF_FAT | GO:0016706~oxidoreductase activity, acting on paired donors, with incorporation or reduction of molecular oxygen, 2-oxoglutarate as one donor, and incorporation of one atom each of oxygen into both donors | 27 | 0.02031565 |  | 27 | 0.01727034 |
| GOTERM_MF_FAT | GO:0004177~aminopeptidase activity | 25 | 0.00055123 |  | 23 | 0.00792497 |
| GOTERM_MF_FAT | GO:0034062~RNA polymerase activity | 25 | 0.00172351 |  | 25 | 0.00142514 |
| GOTERM_MF_FAT | GO:0003899~DNA-directed RNA polymerase activity | 25 | 0.00172351 |  | 25 | 0.00142514 |
| GOTERM_MF_FAT | GO:0018024~histone-lysine N-methyltransferase activity | 25 | 0.00447891 |  | 25 | 0.0037478 |
| GOTERM_MF_FAT | GO:0003730~mRNA 3'-UTR binding | 24 | 0.00257131 |  | 24 | 0.00214695 |
| GOTERM_MF_FAT | GO:0016796~exonuclease activity, active with either ribo- or deoxyribonucleic acids and producing 5'-phosphomonoesters | 24 | 0.00257131 |  | 24 | 0.00214695 |
| GOTERM_MF_FAT | GO:0004520~endodeoxyribonuclease activity | 24 | 0.02738297 |  | 26 | 0.0025538 |
| GOTERM_MF_FAT | GO:0019213~deacetylase activity | 23 | 0.01962775 |  | 22 | 0.04360647 |
| GOTERM_MF_FAT | GO:0016409~palmitoyltransferase activity | 23 | 0.03697505 |  | 24 | 0.01205869 |
| GOTERM_MF_FAT | GO:0043022~ribosome binding | 23 | 0.03697505 |  | 23 | 0.0322665 |
| GOTERM_MF_FAT | GO:0004003~ATP-dependent DNA helicase activity | 22 | 0.00055622 |  | 22 | 0.00046241 |
| GOTERM_MF_FAT | GO:0016866~intramolecular transferase activity | 22 | 0.01324762 |  | 22 | 0.01140918 |
| GOTERM_MF_FAT | GO:0004521~endoribonuclease activity | 22 | 0.02709774 |  | 22 | 0.02360395 |
| GOTERM_MF_FAT | GO:0017112~Rab guanyl-nucleotide exchange factor activity | 21 | 0.00304095 |  | 20 | 0.01043329 |
| GOTERM_MF_FAT | GO:0042162~telomeric DNA binding | 20 | 0.02633901 |  | 20 | 0.02311902 |
| GOTERM_MF_FAT | GO:0034061~DNA polymerase activity | 20 | 0.02633901 |  | 20 | 0.02311902 |
| GOTERM_MF_FAT | GO:0004532~exoribonuclease activity | 19 | 0.00692647 |  | 19 | 0.00600003 |
| GOTERM_MF_FAT | GO:0004298~threonine-type endopeptidase activity | 18 | 0.01035708 |  | 18 | 0.00905894 |
| GOTERM_MF_FAT | GO:0000175~3'-5'-exoribonuclease activity | 18 | 0.01035708 |  | 18 | 0.00905894 |
| GOTERM_MF_FAT | GO:0017069~snRNA binding | 18 | 0.01035708 |  | 18 | 0.00905894 |
| GOTERM_MF_FAT | GO:0070003~threonine-type peptidase activity | 18 | 0.01035708 |  | 18 | 0.00905894 |
| GOTERM_MF_FAT | GO:0016896~exoribonuclease activity, producing 5'-phosphomonoesters | 18 | 0.01035708 |  | 18 | 0.00905894 |
| GOTERM_MF_FAT | GO:0032451~demethylase activity | 16 | 0.02266351 |  | 16 | 0.02020756 |
| GOTERM_MF_FAT | GO:0003887~DNA-directed DNA polymerase activity | 16 | 0.04997855 |  | 16 | 0.04505332 |
| GOTERM_MF_FAT | GO:0016893~endonuclease activity, active with either ribo- or deoxyribonucleic acids and producing 5'-phosphomonoesters | 16 | 0.04997855 |  | 17 | 0.01358203 |
| GOTERM_MF_FAT | GO:0052866~phosphatidylinositol phosphate phosphatase activity | 15 | 0.03311673 |  | 15 | 0.02981101 |
| GOTERM_MF_FAT | GO:0010485~H4 histone acetyltransferase activity | 14 | 0.00552055 |  | 14 | 0.00490702 |
| GOTERM_MF_FAT | GO:0043531~ADP binding | 14 | 0.04794054 |  | 14 | 0.04356606 |
| GOTERM_MF_FAT | GO:0032452~histone demethylase activity | 13 | 0.0088905 |  | 13 | 0.00798038 |
| GOTERM_MF_FAT | GO:0009982~pseudouridine synthase activity | 13 | 0.02911018 |  | 13 | 0.0264205 |
| GOTERM_MF_FAT | GO:0042800~histone methyltransferase activity (H3-K4 specific) | 12 | 0.04371046 |  | 12 | 0.04005355 |
| GOTERM_MF_FAT | GO:0005487~nucleocytoplasmic transporter activity | 11 | 0.02270312 |  | 11 | 0.02078131 |
| GOTERM_MF_FAT | GO:0015923~mannosidase activity | 10 | 0.03593891 |  | 10 | 0.03321814 |
| GOTERM_MF_FAT | GO:0003906~DNA-(apurinic or apyrimidinic site) lyase activity | 10 | 0.03593891 |  | 10 | 0.03321814 |
| GOTERM_MF_FAT | GO:0004559~alpha-mannosidase activity | 10 | 0.03593891 |  | 10 | 0.03321814 |
| GOTERM_MF_FAT | GO:0005543~phospholipid binding | 145 | 0.01164023 |  |  |  |
| GOTERM_MF_FAT | GO:0008238~exopeptidase activity | 53 | 0.00915674 |  |  |  |
| GOTERM_MF_FAT | GO:0004714~transmembrane receptor protein tyrosine kinase activity | 33 | 0.03244875 |  |  |  |
| GOTERM_MF_FAT | GO:0030374~ligand-dependent nuclear receptor transcription coactivator activity | 25 | 0.03645992 |  |  |  |
| GOTERM_MF_FAT | GO:0031418~L-ascorbic acid binding | 15 | 0.03311673 |  |  |  |
| GOTERM_MF_FAT | GO:0035173~histone kinase activity | 14 | 0.0192179 |  |  |  |
| GOTERM_MF_FAT | GO:0030545~receptor regulator activity | 12 | 0.04371046 |  |  |  |
| GOTERM_MF_FAT | GO:0005525~GTP binding |  |  |  |  | 0.04648675 |
| GOTERM_MF_FAT | GO:0005089~Rho guanyl-nucleotide exchange factor activity |  |  |  |  | 0.02842931 |
| GOTERM_MF_FAT | GO:0001104~RNA polymerase II transcription cofactor activity |  |  |  |  | 0.03995344 |
| GOTERM_MF_FAT | GO:0016879~ligase activity, forming carbon-nitrogen bonds |  |  |  |  | 0.04055572 |
| GOTERM_MF_FAT | GO:0003727~single-stranded RNA binding |  |  |  |  | 0.02170937 |
| GOTERM_MF_FAT | GO:0016620~oxidoreductase activity, acting on the aldehyde or oxo group of donors, NAD or NADP as acceptor |  |  |  |  | 0.03262521 |
| GOTERM_MF_FAT | GO:0016417~S-acyltransferase activity |  |  |  |  | 0.04469615 |
| GOTERM_MF_FAT | GO:0033558~protein deacetylase activity |  |  |  |  | 0.04527575 |
| GOTERM_MF_FAT | GO:0004629~phospholipase C activity |  |  |  |  | 0.02981101 |
| GOTERM_MF_FAT | GO:0016799~hydrolase activity, hydrolyzing N-glycosyl compounds |  |  |  |  | 0.02981101 |
| GOTERM_MF_FAT | GO:0004435~phosphatidylinositol phospholipase C activity |  |  |  |  | 0.02981101 |
| GOTERM_MF_FAT | GO:0015095~magnesium ion transmembrane transporter activity |  |  |  |  | 0.04005355 |
