# Supplementary material for: Transcriptome Analysis Reveals That Alfalfa Promotes Rumen Development Through Enhanced Metabolic Processes and Calcium Transduction in Hu Lambs
Source: Front Genet. 2019 Oct 3;10:929. doi: 10.3389/fgene.2019.00929 (PMC6785638; doi:10.3389/fgene.2019.00929)
Supplement: Supplementary file 5 [file Table_5.docx]

**TABLE S5|** All GO terms enriched among the 181 genes shared by the U-N pattern of Hu lambs fed with milk or the starter diet with (S-ALF) or without alfalfa supplementation (STA).

| Category | Term | Number of genes | *P*-value |
| --- | --- | --- | --- |
| GOTERM_BP_FAT | GO:0044248~cellular catabolic process | 16 | 0.001354 |
| GOTERM_BP_FAT | GO:0051186~cofactor metabolic process | 14 | 5.05E-09 |
| GOTERM_BP_FAT | GO:0044712~single-organism catabolic process | 14 | 1.43E-05 |
| GOTERM_BP_FAT | GO:0019637~organophosphate metabolic process | 13 | 0.001299 |
| GOTERM_BP_FAT | GO:0009117~nucleotide metabolic process | 12 | 5.99E-05 |
| GOTERM_BP_FAT | GO:0006753~nucleoside phosphate metabolic process | 12 | 7.85E-05 |
| GOTERM_BP_FAT | GO:0055086~nucleobase-containing small molecule metabolic process | 12 | 0.000155 |
| GOTERM_BP_FAT | GO:0006629~lipid metabolic process | 12 | 0.018475 |
| GOTERM_BP_FAT | GO:0006732~coenzyme metabolic process | 11 | 6.35E-07 |
| GOTERM_BP_FAT | GO:0009259~ribonucleotide metabolic process | 9 | 0.000772 |
| GOTERM_BP_FAT | GO:0019693~ribose phosphate metabolic process | 9 | 0.000952 |
| GOTERM_BP_FAT | GO:0008610~lipid biosynthetic process | 9 | 0.002076 |
| GOTERM_BP_FAT | GO:0019752~carboxylic acid metabolic process | 9 | 0.021036 |
| GOTERM_BP_FAT | GO:0043436~oxoacid metabolic process | 9 | 0.021566 |
| GOTERM_BP_FAT | GO:0006082~organic acid metabolic process | 9 | 0.038119 |
| GOTERM_BP_FAT | GO:0044255~cellular lipid metabolic process | 9 | 0.043974 |
| GOTERM_BP_FAT | GO:0051188~cofactor biosynthetic process | 8 | 1.14E-05 |
| GOTERM_BP_FAT | GO:0009119~ribonucleoside metabolic process | 8 | 0.000411 |
| GOTERM_BP_FAT | GO:0009116~nucleoside metabolic process | 8 | 0.00064 |
| GOTERM_BP_FAT | GO:1901657~glycosyl compound metabolic process | 8 | 0.0008 |
| GOTERM_BP_FAT | GO:0009150~purine ribonucleotide metabolic process | 8 | 0.003001 |
| GOTERM_BP_FAT | GO:0006163~purine nucleotide metabolic process | 8 | 0.004294 |
| GOTERM_BP_FAT | GO:0072521~purine-containing compound metabolic process | 8 | 0.006092 |
| GOTERM_BP_FAT | GO:0006790~sulfur compound metabolic process | 7 | 0.00143 |
| GOTERM_BP_FAT | GO:0046128~purine ribonucleoside metabolic process | 7 | 0.001855 |
| GOTERM_BP_FAT | GO:0042278~purine nucleoside metabolic process | 7 | 0.001974 |
| GOTERM_BP_FAT | GO:0032787~monocarboxylic acid metabolic process | 7 | 0.017306 |
| GOTERM_BP_FAT | GO:0016042~lipid catabolic process | 6 | 0.008838 |
| GOTERM_BP_FAT | GO:0019216~regulation of lipid metabolic process | 6 | 0.012452 |
| GOTERM_BP_FAT | GO:1901361~organic cyclic compound catabolic process | 6 | 0.013039 |
| GOTERM_BP_FAT | GO:0044283~small molecule biosynthetic process | 6 | 0.046711 |
| GOTERM_BP_FAT | GO:0090407~organophosphate biosynthetic process | 6 | 0.048081 |
| GOTERM_BP_FAT | GO:0009132~nucleoside diphosphate metabolic process | 5 | 0.001117 |
| GOTERM_BP_FAT | GO:0009108~coenzyme biosynthetic process | 5 | 0.002395 |
| GOTERM_BP_FAT | GO:1901565~organonitrogen compound catabolic process | 5 | 0.025651 |
| GOTERM_BP_FAT | GO:0009165~nucleotide biosynthetic process | 5 | 0.040732 |
| GOTERM_BP_FAT | GO:0051260~protein homooligomerization | 5 | 0.041516 |
| GOTERM_BP_FAT | GO:0019439~aromatic compound catabolic process | 5 | 0.043108 |
| GOTERM_BP_FAT | GO:1901293~nucleoside phosphate biosynthetic process | 5 | 0.045561 |
| GOTERM_BP_FAT | GO:0006730~one-carbon metabolic process | 4 | 0.000902 |
| GOTERM_BP_FAT | GO:0009185~ribonucleoside diphosphate metabolic process | 4 | 0.00794 |
| GOTERM_BP_FAT | GO:0019362~pyridine nucleotide metabolic process | 4 | 0.015369 |
| GOTERM_BP_FAT | GO:0046496~nicotinamide nucleotide metabolic process | 4 | 0.015369 |
| GOTERM_BP_FAT | GO:0072524~pyridine-containing compound metabolic process | 4 | 0.016009 |
| GOTERM_BP_FAT | GO:0006733~oxidoreduction coenzyme metabolic process | 4 | 0.022439 |
| GOTERM_BP_FAT | GO:0045834~positive regulation of lipid metabolic process | 4 | 0.024033 |
| GOTERM_BP_FAT | GO:0044242~cellular lipid catabolic process | 4 | 0.043244 |
| GOTERM_BP_FAT | GO:0045980~negative regulation of nucleotide metabolic process | 3 | 0.019735 |
| GOTERM_BP_FAT | GO:0006637~acyl-CoA metabolic process | 3 | 0.039163 |
| GOTERM_BP_FAT | GO:0035383~thioester metabolic process | 3 | 0.039163 |
| GOTERM_BP_FAT | GO:0006096~glycolytic process | 3 | 0.039163 |
| GOTERM_BP_FAT | GO:0046939~nucleotide phosphorylation | 3 | 0.041013 |
| GOTERM_BP_FAT | GO:0006757~ATP generation from ADP | 3 | 0.041013 |
| GOTERM_BP_FAT | GO:0043648~dicarboxylic acid metabolic process | 3 | 0.041013 |
| GOTERM_BP_FAT | GO:0006165~nucleoside diphosphate phosphorylation | 3 | 0.041013 |
| GOTERM_CC_FAT | GO:0005739~mitochondrion | 29 | 5.75E-07 |
| GOTERM_CC_FAT | GO:0044429~mitochondrial part | 13 | 0.000308 |
| GOTERM_CC_FAT | GO:0005740~mitochondrial envelope | 11 | 0.000638 |
| GOTERM_CC_FAT | GO:0031966~mitochondrial membrane | 10 | 0.001604 |
| GOTERM_CC_FAT | GO:0031967~organelle envelope | 12 | 0.008465 |
| GOTERM_CC_FAT | GO:0031975~envelope | 12 | 0.009053 |
| GOTERM_CC_FAT | GO:0042579~microbody | 4 | 0.038586 |
| GOTERM_CC_FAT | GO:0005777~peroxisome | 4 | 0.038586 |
| GOTERM_CC_FAT | GO:0005743~mitochondrial inner membrane | 6 | 0.049195 |
| GOTERM_MF_FAT | GO:0046914~transition metal ion binding | 15 | 0.034278 |
| GOTERM_MF_FAT | GO:0048037~cofactor binding | 8 | 0.000953 |
| GOTERM_MF_FAT | GO:0050662~coenzyme binding | 6 | 0.003897 |
| GOTERM_MF_FAT | GO:0016830~carbon-carbon lyase activity | 4 | 0.002822 |
| GOTERM_MF_FAT | GO:0016614~oxidoreductase activity, acting on CH-OH group of donors | 4 | 0.036623 |
| GOTERM_MF_FAT | GO:0016836~hydro-lyase activity | 3 | 0.031103 |
| GOTERM_MF_FAT | GO:0016627~oxidoreductase activity, acting on the CH-CH group of donors | 3 | 0.043076 |
