# Supplementary material for: Transcriptome Analysis Reveals That Alfalfa Promotes Rumen Development Through Enhanced Metabolic Processes and Calcium Transduction in Hu Lambs
Source: Front Genet. 2019 Oct 3;10:929. doi: 10.3389/fgene.2019.00929 (PMC6785638; doi:10.3389/fgene.2019.00929)
Supplement: Supplementary file 6 [file Table_6.docx]

**TABLE S6|** All GO terms enriched in the 399 genes shared by the D-N pattern of Hu lambs fed with milk or the starter diet with (S-ALF) or without alfalfa supplementation (STA).

| Category | Term | Number of genes | *P*-value |
| --- | --- | --- | --- |
| GOTERM_BP_FAT | GO:0007399~nervous system development | 61 | 1.30E-12 |
| GOTERM_BP_FAT | GO:0048468~cell development | 54 | 8.34E-09 |
| GOTERM_BP_FAT | GO:0022008~neurogenesis | 49 | 1.76E-11 |
| GOTERM_BP_FAT | GO:0023051~regulation of signaling | 47 | 0.0155145 |
| GOTERM_BP_FAT | GO:0048699~generation of neurons | 45 | 2.06E-10 |
| GOTERM_BP_FAT | GO:0010646~regulation of cell communication | 45 | 0.03110201 |
| GOTERM_BP_FAT | GO:0030182~neuron differentiation | 42 | 2.69E-10 |
| GOTERM_BP_FAT | GO:0003008~system process | 40 | 1.08E-07 |
| GOTERM_BP_FAT | GO:0030030~cell projection organization | 37 | 7.16E-07 |
| GOTERM_BP_FAT | GO:2000026~regulation of multicellular organismal development | 37 | 7.05E-04 |
| GOTERM_BP_FAT | GO:0048666~neuron development | 35 | 3.25E-09 |
| GOTERM_BP_FAT | GO:0031175~neuron projection development | 34 | 8.01E-11 |
| GOTERM_BP_FAT | GO:0045595~regulation of cell differentiation | 32 | 0.00303782 |
| GOTERM_BP_FAT | GO:0000902~cell morphogenesis | 30 | 5.31E-04 |
| GOTERM_BP_FAT | GO:0032989~cellular component morphogenesis | 30 | 0.00137696 |
| GOTERM_BP_FAT | GO:0051049~regulation of transport | 30 | 0.00979918 |
| GOTERM_BP_FAT | GO:0007267~cell-cell signaling | 29 | 1.76E-05 |
| GOTERM_BP_FAT | GO:0051240~positive regulation of multicellular organismal process | 29 | 0.0072075 |
| GOTERM_BP_FAT | GO:0050877~neurological system process | 27 | 9.34E-07 |
| GOTERM_BP_FAT | GO:0007610~behavior | 26 | 2.82E-08 |
| GOTERM_BP_FAT | GO:0048858~cell projection morphogenesis | 25 | 3.22E-05 |
| GOTERM_BP_FAT | GO:0032990~cell part morphogenesis | 25 | 5.28E-05 |
| GOTERM_BP_FAT | GO:0048812~neuron projection morphogenesis | 24 | 3.82E-08 |
| GOTERM_BP_FAT | GO:0051960~regulation of nervous system development | 24 | 3.26E-05 |
| GOTERM_BP_FAT | GO:0000904~cell morphogenesis involved in differentiation | 24 | 4.51E-05 |
| GOTERM_BP_FAT | GO:0060284~regulation of cell development | 23 | 2.75E-04 |
| GOTERM_BP_FAT | GO:0050767~regulation of neurogenesis | 22 | 2.64E-05 |
| GOTERM_BP_FAT | GO:0044708~single-organism behavior | 21 | 1.28E-07 |
| GOTERM_BP_FAT | GO:0051094~positive regulation of developmental process | 21 | 0.04686761 |
| GOTERM_BP_FAT | GO:0048667~cell morphogenesis involved in neuron differentiation | 20 | 4.01E-06 |
| GOTERM_BP_FAT | GO:0046903~secretion | 20 | 0.01077945 |
| GOTERM_BP_FAT | GO:0006811~ion transport | 19 | 0.00913935 |
| GOTERM_BP_FAT | GO:0061564~axon development | 18 | 5.79E-06 |
| GOTERM_BP_FAT | GO:0045664~regulation of neuron differentiation | 18 | 1.69E-04 |
| GOTERM_BP_FAT | GO:0007417~central nervous system development | 18 | 0.01518388 |
| GOTERM_BP_FAT | GO:0098916~anterograde trans-synaptic signaling | 17 | 7.08E-06 |
| GOTERM_BP_FAT | GO:0007268~chemical synaptic transmission | 17 | 7.08E-06 |
| GOTERM_BP_FAT | GO:0099537~trans-synaptic signaling | 17 | 7.08E-06 |
| GOTERM_BP_FAT | GO:0099536~synaptic signaling | 17 | 7.08E-06 |
| GOTERM_BP_FAT | GO:0031344~regulation of cell projection organization | 16 | 9.11E-04 |
| GOTERM_BP_FAT | GO:0007409~axonogenesis | 15 | 1.65E-04 |
| GOTERM_BP_FAT | GO:0010975~regulation of neuron projection development | 14 | 4.86E-04 |
| GOTERM_BP_FAT | GO:0071705~nitrogen compound transport | 14 | 0.00207301 |
| GOTERM_BP_FAT | GO:0042330~taxis | 14 | 0.00873017 |
| GOTERM_BP_FAT | GO:0006935~chemotaxis | 14 | 0.00873017 |
| GOTERM_BP_FAT | GO:0003012~muscle system process | 13 | 1.89E-04 |
| GOTERM_BP_FAT | GO:0007186~G-protein coupled receptor signaling pathway | 13 | 0.0019855 |
| GOTERM_BP_FAT | GO:0051962~positive regulation of nervous system development | 13 | 0.00346536 |
| GOTERM_BP_FAT | GO:0007423~sensory organ development | 13 | 0.04866829 |
| GOTERM_BP_FAT | GO:0006812~cation transport | 13 | 0.04866829 |
| GOTERM_BP_FAT | GO:0030534~adult behavior | 12 | 8.19E-06 |
| GOTERM_BP_FAT | GO:0050808~synapse organization | 12 | 9.78E-05 |
| GOTERM_BP_FAT | GO:0006936~muscle contraction | 12 | 1.32E-04 |
| GOTERM_BP_FAT | GO:0007626~locomotory behavior | 12 | 1.57E-04 |
| GOTERM_BP_FAT | GO:0044057~regulation of system process | 12 | 0.00425942 |
| GOTERM_BP_FAT | GO:0008015~blood circulation | 12 | 0.00438928 |
| GOTERM_BP_FAT | GO:0003013~circulatory system process | 12 | 0.0047949 |
| GOTERM_BP_FAT | GO:0007600~sensory perception | 12 | 0.01149773 |
| GOTERM_BP_FAT | GO:0022604~regulation of cell morphogenesis | 12 | 0.04490433 |
| GOTERM_BP_FAT | GO:0023061~signal release | 11 | 0.00480795 |
| GOTERM_BP_FAT | GO:0034220~ion transmembrane transport | 11 | 0.01009241 |
| GOTERM_BP_FAT | GO:0043269~regulation of ion transport | 11 | 0.01095323 |
| GOTERM_BP_FAT | GO:0007612~learning | 10 | 3.33E-05 |
| GOTERM_BP_FAT | GO:0060047~heart contraction | 10 | 7.99E-05 |
| GOTERM_BP_FAT | GO:0003015~heart process | 10 | 9.39E-05 |
| GOTERM_BP_FAT | GO:0007611~learning or memory | 10 | 7.40E-04 |
| GOTERM_BP_FAT | GO:0050890~cognition | 10 | 0.0018592 |
| GOTERM_BP_FAT | GO:0042391~regulation of membrane potential | 10 | 0.00685002 |
| GOTERM_BP_FAT | GO:0006941~striated muscle contraction | 9 | 1.65E-04 |
| GOTERM_BP_FAT | GO:0001505~regulation of neurotransmitter levels | 9 | 1.95E-04 |
| GOTERM_BP_FAT | GO:0010001~glial cell differentiation | 9 | 0.00147846 |
| GOTERM_BP_FAT | GO:0007411~axon guidance | 9 | 0.00226563 |
| GOTERM_BP_FAT | GO:0097485~neuron projection guidance | 9 | 0.00263385 |
| GOTERM_BP_FAT | GO:0050804~modulation of synaptic transmission | 9 | 0.00459643 |
| GOTERM_BP_FAT | GO:0042063~gliogenesis | 9 | 0.00617232 |
| GOTERM_BP_FAT | GO:0050803~regulation of synapse structure or activity | 9 | 0.00642732 |
| GOTERM_BP_FAT | GO:0050769~positive regulation of neurogenesis | 9 | 0.04340889 |
| GOTERM_BP_FAT | GO:0060048~cardiac muscle contraction | 8 | 7.25E-05 |
| GOTERM_BP_FAT | GO:0006836~neurotransmitter transport | 8 | 3.68E-04 |
| GOTERM_BP_FAT | GO:0008016~regulation of heart contraction | 8 | 0.00134311 |
| GOTERM_BP_FAT | GO:0032412~regulation of ion transmembrane transporter activity | 8 | 0.00185214 |
| GOTERM_BP_FAT | GO:0022898~regulation of transmembrane transporter activity | 8 | 0.00222254 |
| GOTERM_BP_FAT | GO:0032409~regulation of transporter activity | 8 | 0.00349212 |
| GOTERM_BP_FAT | GO:1903522~regulation of blood circulation | 8 | 0.00605913 |
| GOTERM_BP_FAT | GO:0016358~dendrite development | 8 | 0.01066445 |
| GOTERM_BP_FAT | GO:0034765~regulation of ion transmembrane transport | 8 | 0.02510093 |
| GOTERM_BP_FAT | GO:0045666~positive regulation of neuron differentiation | 8 | 0.03198115 |
| GOTERM_BP_FAT | GO:0034762~regulation of transmembrane transport | 8 | 0.03198115 |
| GOTERM_BP_FAT | GO:0098662~inorganic cation transmembrane transport | 8 | 0.04567901 |
| GOTERM_BP_FAT | GO:0098660~inorganic ion transmembrane transport | 8 | 0.04793021 |
| GOTERM_BP_FAT | GO:0010959~regulation of metal ion transport | 8 | 0.04793021 |
| GOTERM_BP_FAT | GO:0007416~synapse assembly | 7 | 0.00306741 |
| GOTERM_BP_FAT | GO:0007605~sensory perception of sound | 7 | 0.00781236 |
| GOTERM_BP_FAT | GO:0050954~sensory perception of mechanical stimulus | 7 | 0.01208894 |
| GOTERM_BP_FAT | GO:0030100~regulation of endocytosis | 7 | 0.03424442 |
| GOTERM_BP_FAT | GO:0010977~negative regulation of neuron projection development | 6 | 0.00909305 |
| GOTERM_BP_FAT | GO:0050807~regulation of synapse organization | 6 | 0.01666596 |
| GOTERM_BP_FAT | GO:0031345~negative regulation of cell projection organization | 6 | 0.01838774 |
| GOTERM_BP_FAT | GO:0090257~regulation of muscle system process | 6 | 0.02643481 |
| GOTERM_BP_FAT | GO:0060041~retina development in camera-type eye | 6 | 0.036441 |
| GOTERM_BP_FAT | GO:0007215~glutamate receptor signaling pathway | 5 | 0.00153953 |
| GOTERM_BP_FAT | GO:0048814~regulation of dendrite morphogenesis | 5 | 0.00928399 |
| GOTERM_BP_FAT | GO:0048708~astrocyte differentiation | 5 | 0.00928399 |
| GOTERM_BP_FAT | GO:0021782~glial cell development | 5 | 0.01255515 |
| GOTERM_BP_FAT | GO:0099643~signal release from synapse | 5 | 0.01543772 |
| GOTERM_BP_FAT | GO:0007269~neurotransmitter secretion | 5 | 0.01543772 |
| GOTERM_BP_FAT | GO:0001508~action potential | 5 | 0.01648358 |
| GOTERM_BP_FAT | GO:0099531~presynaptic process involved in chemical synaptic transmission | 5 | 0.01870598 |
| GOTERM_BP_FAT | GO:0050806~positive regulation of synaptic transmission | 5 | 0.02110573 |
| GOTERM_BP_FAT | GO:0051963~regulation of synapse assembly | 5 | 0.0264499 |
| GOTERM_BP_FAT | GO:0048813~dendrite morphogenesis | 5 | 0.02939937 |
| GOTERM_BP_FAT | GO:0050773~regulation of dendrite development | 5 | 0.03937745 |
| GOTERM_BP_FAT | GO:0008344~adult locomotory behavior | 5 | 0.03937745 |
| GOTERM_BP_FAT | GO:0006937~regulation of muscle contraction | 5 | 0.04500687 |
| GOTERM_BP_FAT | GO:0017014~protein nitrosylation | 4 | 3.46E-04 |
| GOTERM_BP_FAT | GO:0018119~peptidyl-cysteine S-nitrosylation | 4 | 3.46E-04 |
| GOTERM_BP_FAT | GO:0014002~astrocyte development | 4 | 0.00370885 |
| GOTERM_BP_FAT | GO:0007588~excretion | 4 | 0.00438931 |
| GOTERM_BP_FAT | GO:0018198~peptidyl-cysteine modification | 4 | 0.00514056 |
| GOTERM_BP_FAT | GO:0051966~regulation of synaptic transmission, glutamatergic | 4 | 0.00514056 |
| GOTERM_BP_FAT | GO:0051703~intraspecies interaction between organisms | 4 | 0.00888966 |
| GOTERM_BP_FAT | GO:0035176~social behavior | 4 | 0.00888966 |
| GOTERM_BP_FAT | GO:1903524~positive regulation of blood circulation | 4 | 0.02021305 |
| GOTERM_BP_FAT | GO:0015844~monoamine transport | 4 | 0.02021305 |
| GOTERM_BP_FAT | GO:0051705~multi-organism behavior | 4 | 0.02786569 |
| GOTERM_BP_FAT | GO:0019233~sensory perception of pain | 4 | 0.03219185 |
| GOTERM_BP_FAT | GO:0031110~regulation of microtubule polymerization or depolymerization | 4 | 0.03447885 |
| GOTERM_BP_FAT | GO:0099565~chemical synaptic transmission, postsynaptic | 4 | 0.03447885 |
| GOTERM_BP_FAT | GO:0010171~body morphogenesis | 4 | 0.03447885 |
| GOTERM_BP_FAT | GO:0060078~regulation of postsynaptic membrane potential | 4 | 0.03447885 |
| GOTERM_BP_FAT | GO:0006942~regulation of striated muscle contraction | 4 | 0.04182958 |
| GOTERM_BP_FAT | GO:0048268~clathrin coat assembly | 3 | 0.00699866 |
| GOTERM_BP_FAT | GO:2000311~regulation of alpha-amino-3-hydroxy-5-methyl-4-isoxazole propionate selective glutamate receptor activity | 3 | 0.01744168 |
| GOTERM_BP_FAT | GO:1903861~positive regulation of dendrite extension | 3 | 0.02780855 |
| GOTERM_BP_FAT | GO:1903859~regulation of dendrite extension | 3 | 0.02780855 |
| GOTERM_BP_FAT | GO:0097484~dendrite extension | 3 | 0.03577955 |
| GOTERM_BP_FAT | GO:0002026~regulation of the force of heart contraction | 3 | 0.03577955 |
| GOTERM_BP_FAT | GO:0098743~cell aggregation | 3 | 0.04005564 |
| GOTERM_BP_FAT | GO:0045823~positive regulation of heart contraction | 3 | 0.04005564 |
| GOTERM_BP_FAT | GO:0001502~cartilage condensation | 3 | 0.04005564 |
| GOTERM_BP_FAT | GO:0007274~neuromuscular synaptic transmission | 3 | 0.04005564 |
| GOTERM_BP_FAT | GO:0099601~regulation of neurotransmitter receptor activity | 3 | 0.04451393 |
| GOTERM_BP_FAT | GO:1900449~regulation of glutamate receptor signaling pathway | 3 | 0.04451393 |
| GOTERM_CC_FAT | GO:0005576~extracellular region | 65 | 0.03970393 |
| GOTERM_CC_FAT | GO:0097458~neuron part | 42 | 2.99E-15 |
| GOTERM_CC_FAT | GO:0045202~synapse | 26 | 3.24E-10 |
| GOTERM_CC_FAT | GO:0043005~neuron projection | 26 | 4.98E-09 |
| GOTERM_CC_FAT | GO:0031226~intrinsic component of plasma membrane | 24 | 2.59E-05 |
| GOTERM_CC_FAT | GO:0005887~integral component of plasma membrane | 22 | 8.01E-05 |
| GOTERM_CC_FAT | GO:0005615~extracellular space | 22 | 0.03539986 |
| GOTERM_CC_FAT | GO:0044456~synapse part | 20 | 2.47E-08 |
| GOTERM_CC_FAT | GO:0036477~somatodendritic compartment | 17 | 2.20E-06 |
| GOTERM_CC_FAT | GO:0098590~plasma membrane region | 17 | 0.00374282 |
| GOTERM_CC_FAT | GO:0016023~cytoplasmic, membrane-bounded vesicle | 16 | 0.01664648 |
| GOTERM_CC_FAT | GO:0043025~neuronal cell body | 13 | 1.96E-06 |
| GOTERM_CC_FAT | GO:0098794~postsynapse | 13 | 3.02E-06 |
| GOTERM_CC_FAT | GO:0044297~cell body | 13 | 1.51E-05 |
| GOTERM_CC_FAT | GO:0030424~axon | 13 | 5.76E-05 |
| GOTERM_CC_FAT | GO:1902495~transmembrane transporter complex | 11 | 0.00158292 |
| GOTERM_CC_FAT | GO:1990351~transporter complex | 11 | 0.00179514 |
| GOTERM_CC_FAT | GO:0043235~receptor complex | 11 | 0.0070128 |
| GOTERM_CC_FAT | GO:0098793~presynapse | 10 | 2.62E-04 |
| GOTERM_CC_FAT | GO:0034702~ion channel complex | 10 | 0.00289444 |
| GOTERM_CC_FAT | GO:0043209~myelin sheath | 9 | 0.00328819 |
| GOTERM_CC_FAT | GO:0034703~cation channel complex | 8 | 0.00503537 |
| GOTERM_CC_FAT | GO:0098589~membrane region | 8 | 0.03766466 |
| GOTERM_CC_FAT | GO:0030426~growth cone | 7 | 9.45E-05 |
| GOTERM_CC_FAT | GO:0030427~site of polarized growth | 7 | 1.24E-04 |
| GOTERM_CC_FAT | GO:0030133~transport vesicle | 7 | 0.02336634 |
| GOTERM_CC_FAT | GO:0045121~membrane raft | 7 | 0.03966922 |
| GOTERM_CC_FAT | GO:0098857~membrane microdomain | 7 | 0.03966922 |
| GOTERM_CC_FAT | GO:0097060~synaptic membrane | 6 | 0.01160825 |
| GOTERM_CC_FAT | GO:0005882~intermediate filament | 6 | 0.03452543 |
| GOTERM_CC_FAT | GO:0001518~voltage-gated sodium channel complex | 5 | 9.65E-05 |
| GOTERM_CC_FAT | GO:0034706~sodium channel complex | 5 | 2.20E-04 |
| GOTERM_CC_FAT | GO:0008021~synaptic vesicle | 5 | 0.01944837 |
| GOTERM_CC_FAT | GO:0045211~postsynaptic membrane | 5 | 0.0218423 |
| GOTERM_CC_FAT | GO:0070382~exocytic vesicle | 5 | 0.02575782 |
| GOTERM_CC_FAT | GO:0005883~neurofilament | 3 | 0.00482333 |
| GOTERM_MF_FAT | GO:0008324~cation transmembrane transporter activity | 24 | 4.94E-07 |
| GOTERM_MF_FAT | GO:0005216~ion channel activity | 21 | 1.53E-07 |
| GOTERM_MF_FAT | GO:0022838~substrate-specific channel activity | 21 | 2.55E-07 |
| GOTERM_MF_FAT | GO:0015267~channel activity | 21 | 3.98E-07 |
| GOTERM_MF_FAT | GO:0022803~passive transmembrane transporter activity | 21 | 3.98E-07 |
| GOTERM_MF_FAT | GO:0022836~gated channel activity | 18 | 5.96E-07 |
| GOTERM_MF_FAT | GO:0005509~calcium ion binding | 18 | 0.00278632 |
| GOTERM_MF_FAT | GO:0022890~inorganic cation transmembrane transporter activity | 17 | 1.89E-04 |
| GOTERM_MF_FAT | GO:0046873~metal ion transmembrane transporter activity | 16 | 7.11E-05 |
| GOTERM_MF_FAT | GO:0005261~cation channel activity | 15 | 1.24E-05 |
| GOTERM_MF_FAT | GO:0015077~monovalent inorganic cation transmembrane transporter activity | 15 | 3.48E-05 |
| GOTERM_MF_FAT | GO:0015081~sodium ion transmembrane transporter activity | 11 | 2.64E-06 |
| GOTERM_MF_FAT | GO:0022834~ligand-gated channel activity | 9 | 2.76E-04 |
| GOTERM_MF_FAT | GO:0015276~ligand-gated ion channel activity | 9 | 2.76E-04 |
| GOTERM_MF_FAT | GO:0005244~voltage-gated ion channel activity | 8 | 0.00521394 |
| GOTERM_MF_FAT | GO:0022832~voltage-gated channel activity | 8 | 0.00521394 |
| GOTERM_MF_FAT | GO:0005230~extracellular ligand-gated ion channel activity | 7 | 7.08E-04 |
| GOTERM_MF_FAT | GO:0005272~sodium channel activity | 6 | 9.54E-05 |
| GOTERM_MF_FAT | GO:0015291~secondary active transmembrane transporter activity | 6 | 0.04692086 |
| GOTERM_MF_FAT | GO:1905030~voltage-gated ion channel activity involved in regulation of postsynaptic membrane potential | 5 | 1.77E-04 |
| GOTERM_MF_FAT | GO:0005248~voltage-gated sodium channel activity | 5 | 1.77E-04 |
| GOTERM_MF_FAT | GO:0001948~glycoprotein binding | 5 | 0.01849061 |
| GOTERM_MF_FAT | GO:0016917~GABA receptor activity | 4 | 0.00460731 |
| GOTERM_MF_FAT | GO:0030594~neurotransmitter receptor activity | 4 | 0.02731267 |
| GOTERM_MF_FAT | GO:0005231~excitatory extracellular ligand-gated ion channel activity | 4 | 0.03293274 |
| GOTERM_MF_FAT | GO:0005254~chloride channel activity | 4 | 0.03492976 |
| GOTERM_MF_FAT | GO:0015645~fatty acid ligase activity | 3 | 0.02128429 |
| GOTERM_MF_FAT | GO:0017080~sodium channel regulator activity | 3 | 0.02747512 |
| GOTERM_MF_FAT | GO:0043394~proteoglycan binding | 3 | 0.03429381 |
| GOTERM_MF_FAT | GO:0004890~GABA-A receptor activity | 3 | 0.03429381 |
