# Supplementary figures and images for: Transcriptome Analysis Reveals That Alfalfa Promotes Rumen Development Through Enhanced Metabolic Processes and Calcium Transduction in Hu Lambs
Source: Front Genet. 2019 Oct 3;10:929. doi: 10.3389/fgene.2019.00929 (PMC6785638; doi:10.3389/fgene.2019.00929)

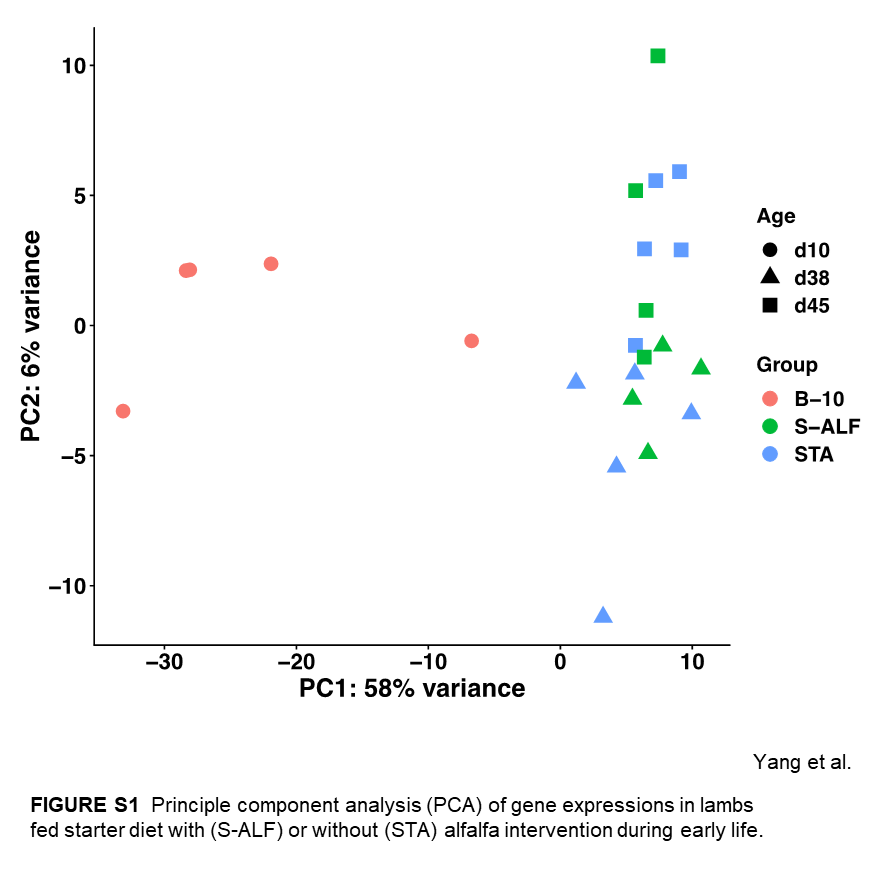

Supplement: Supplementary file 7 [file Image_1.tif]

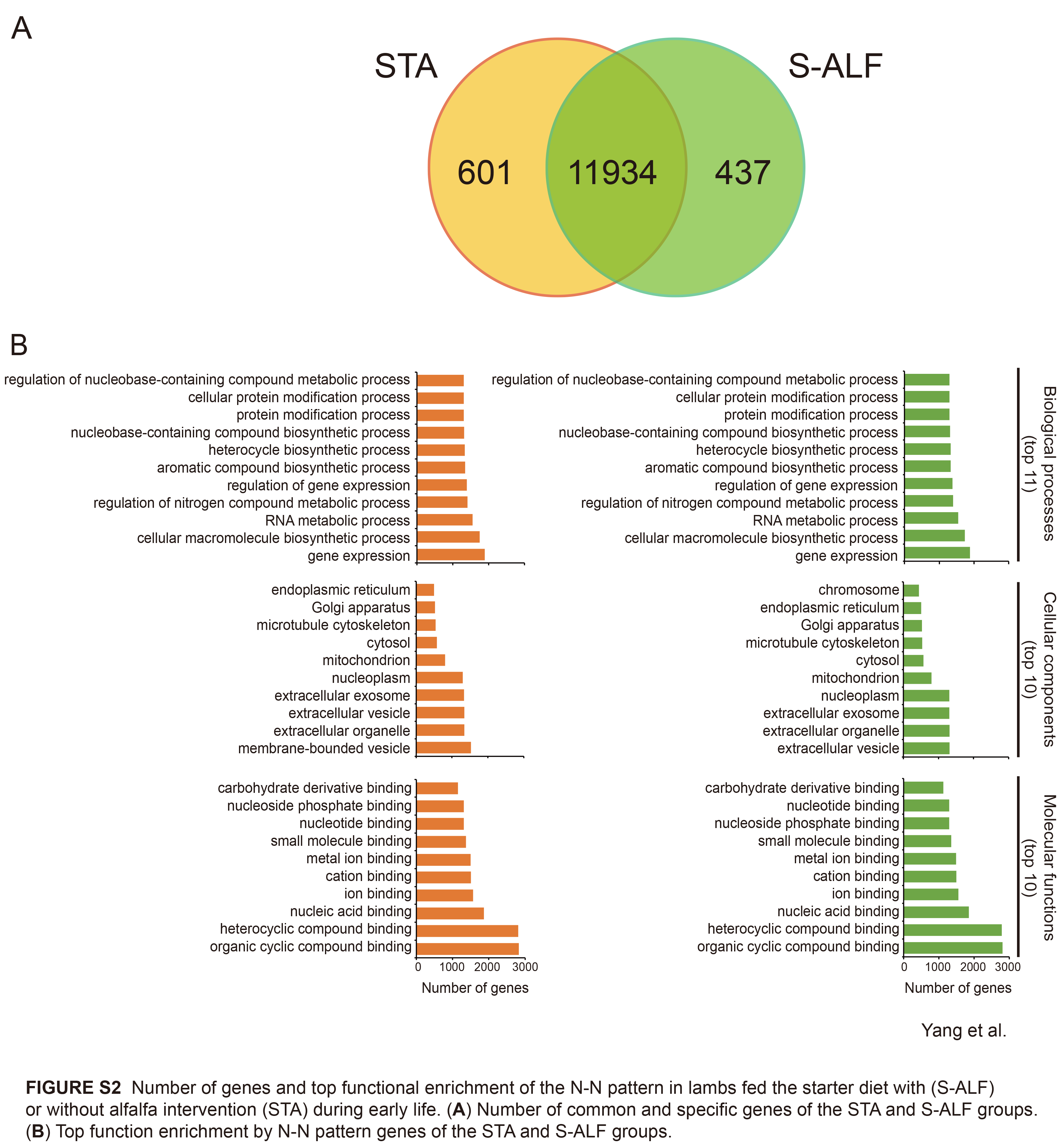

Supplement: Supplementary file 8 [file Image_2.tif]

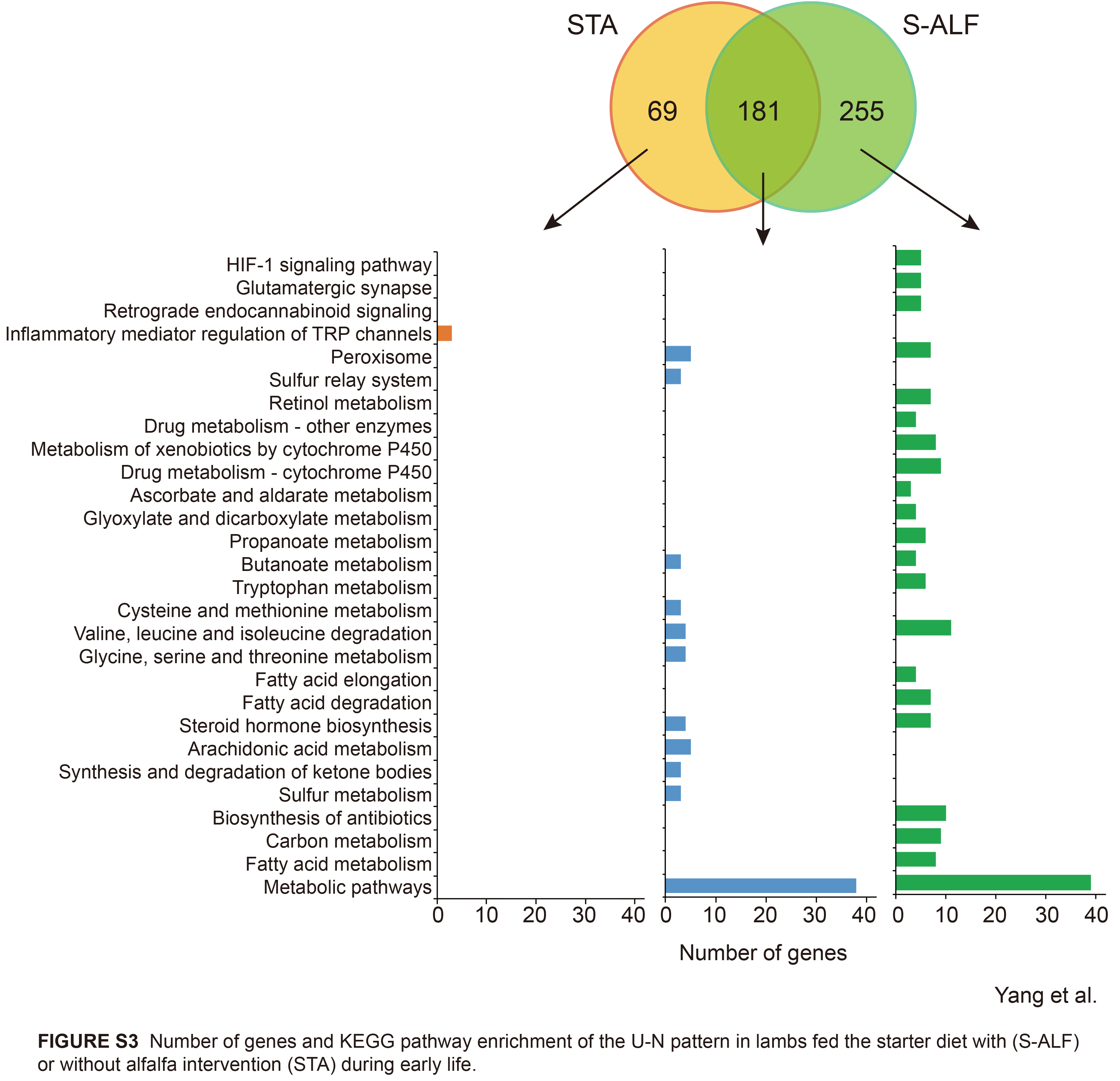

Supplement: Supplementary file 9 [file Image_3.tif]

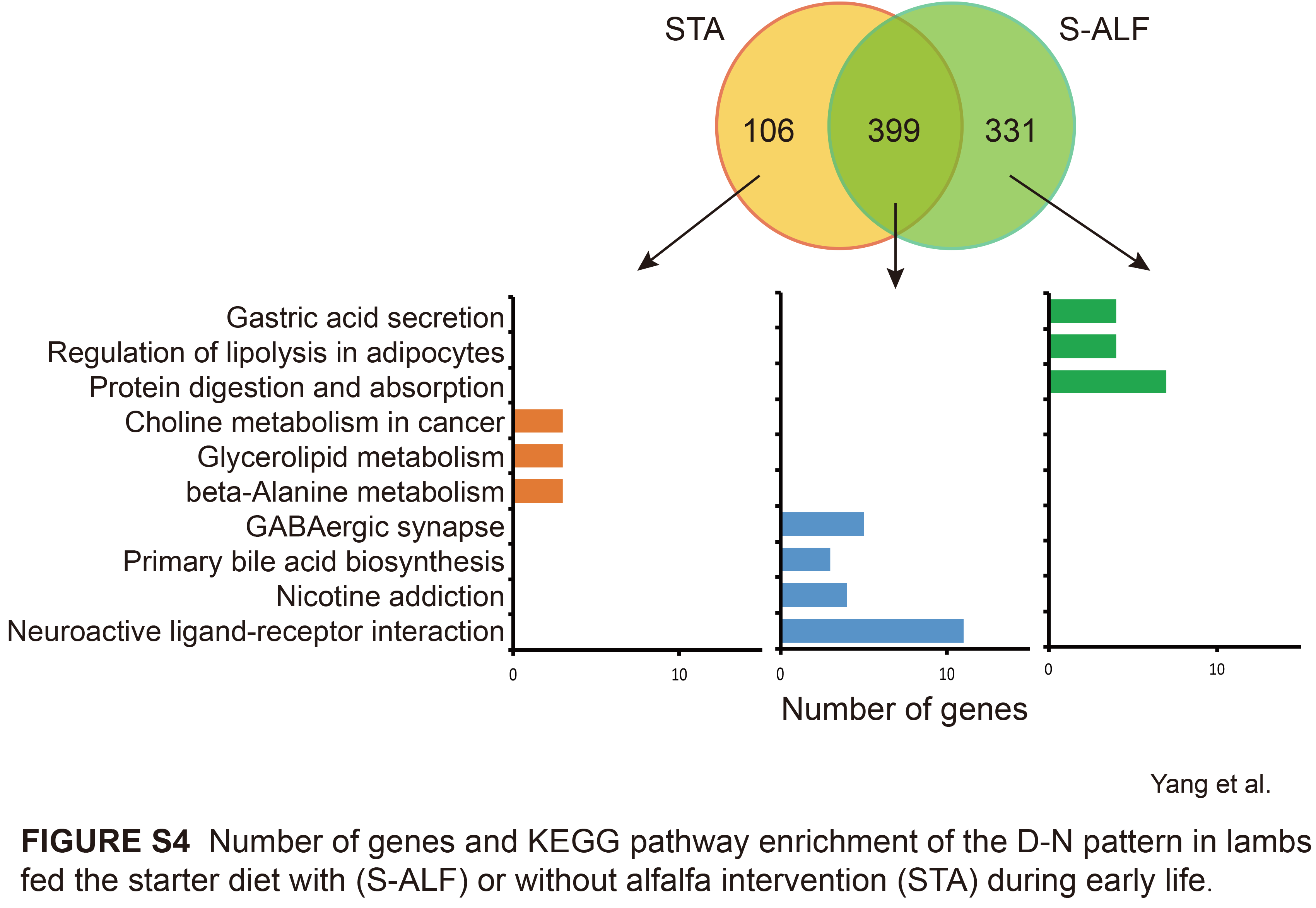

Supplement: Supplementary file 10 [file Image_4.tif]

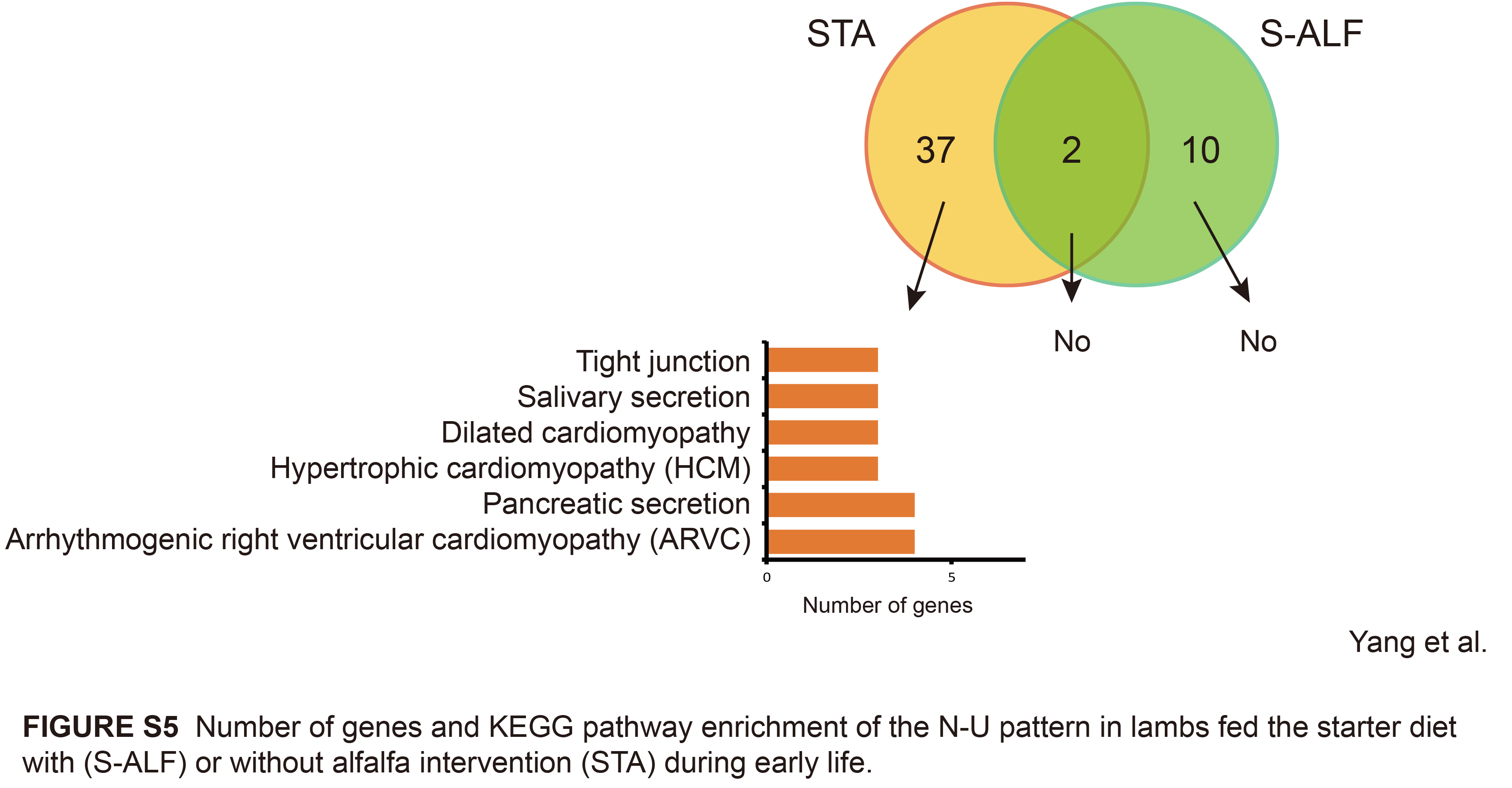

Supplement: Supplementary file 11 [file Image_5.tif]

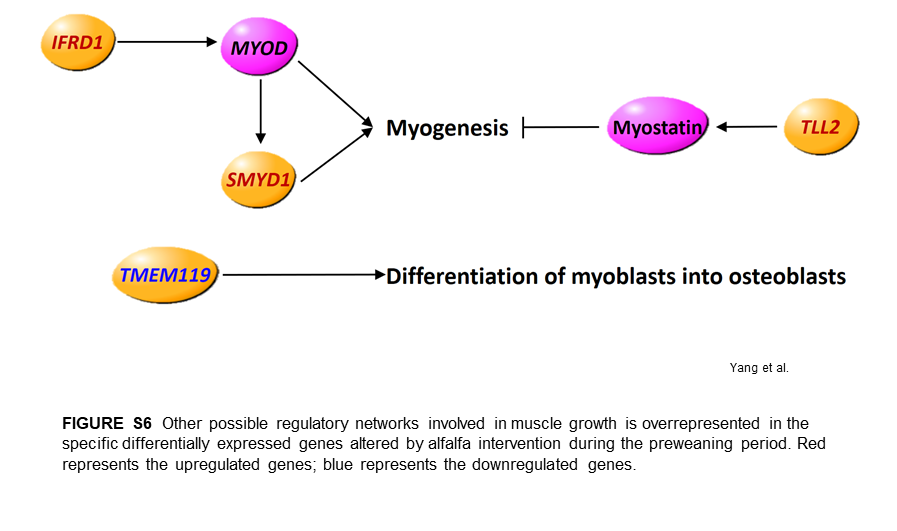

Supplement: Supplementary file 12 [file Image_6.tif]

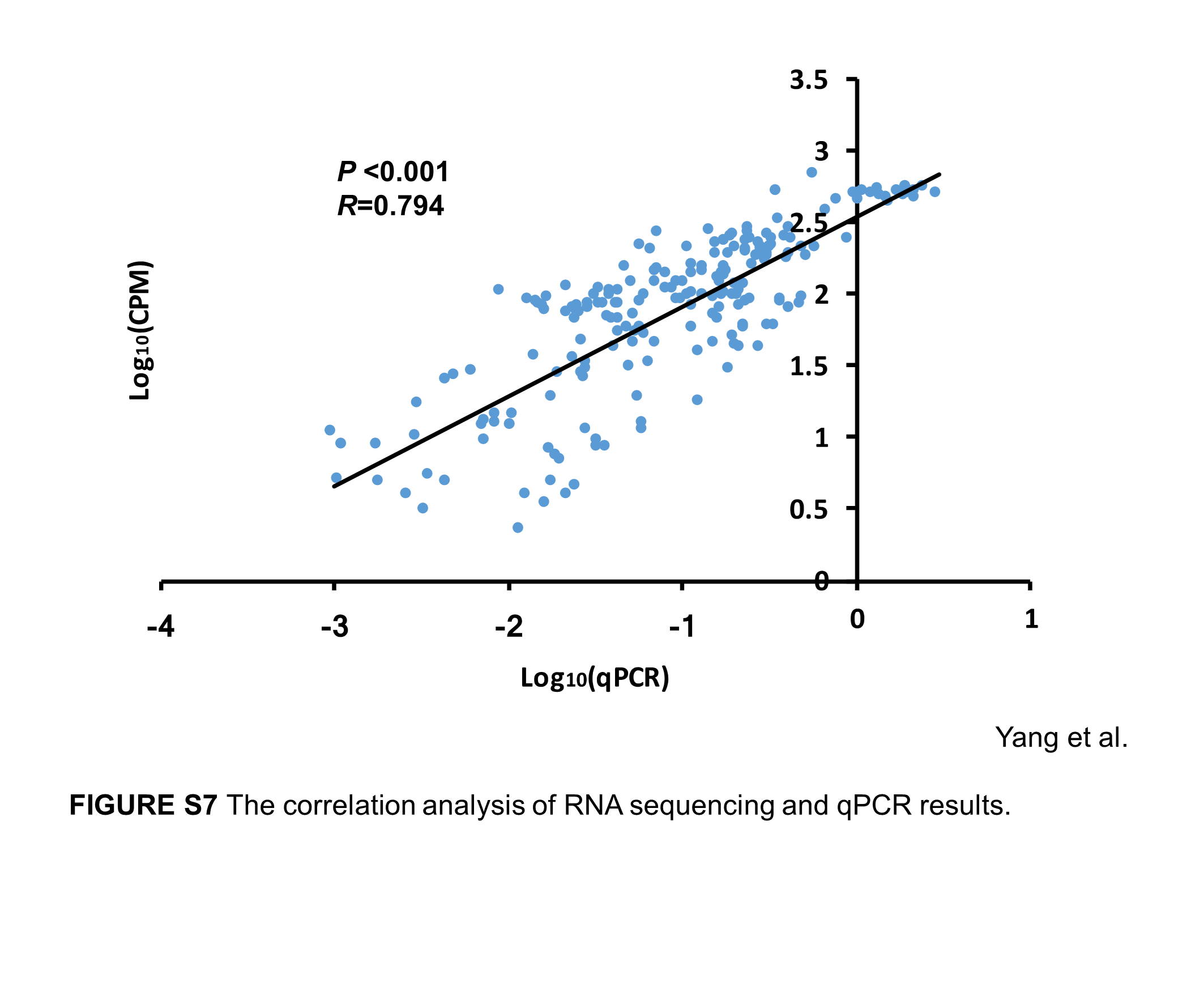

Supplement: Supplementary file 13 [file Image_7.tif]
